# Circulating long non-coding RNA *GAS5* (growth arrest-specific transcript 5) as a complement marker for the detection of malignant mesothelioma using liquid biopsies

**DOI:** 10.1186/s40364-020-00194-4

**Published:** 2020-05-13

**Authors:** Daniel G. Weber, Swaantje Casjens, Alexander Brik, Irina Raiko, Martin Lehnert, Dirk Taeger, Jan Gleichenhagen, Jens Kollmeier, Torsten T. Bauer, Thomas Brüning, Georg Johnen, the MoMar study group, Katarzyna Burek, Bettina Dumont, Olaf Hagemeyer, Heike Heimann, Monika Kobek, Claudia Lechtenfeld, Swetlana Meier, Simone Naumann, Christoph Nöllenheidt, Beate Pesch, Simone Putzke, Hans-Peter Rihs, Peter Rozynek, Sandra Schonefeld, Katja Szafranski, Carmen Töpfer, Katharina Wichert, Thorsten Wiethege, Sandra Zilch-Schöneweis

**Affiliations:** 1 Institute for Prevention and Occupational Medicine of the German Social Accident Insurance – Institute of the Ruhr University Bochum (IPA), Buerkle-de-la-Camp-Platz 1, 44789 Bochum, Germany; 2 Lungenklinik Heckeshorn, Helios Klinikum Emil von Behring, Walterhöferstraße 11, 14165 Berlin, Germany

**Keywords:** Biomarker, Blood, Cancer, Circulating, Early detection, Liquid biopsy, lncRNA, Marker, Mesothelioma, Plasma, qPCR, Screening

## Abstract

**Background:**

For the detection of malignant mesothelioma additional markers are needed besides the established panel consisting of calretinin and mesothelin. The aim of this study was the identification and verification of long non-coding RNAs (lncRNAs) as complementing circulating markers.

**Methods:**

Candidate lncRNAs were identified in silico using previously published RNA expression profiles and verified using quantitative PCR (qPCR) in mesothelioma cell lines as well as human plasma samples from mesothelioma patients and asbestos-exposed controls.

**Results:**

*GAS5* (growth arrest-specific transcript 5) as a single marker is marked by a low sensitivity of 14%, but the combination of *GAS5* with calretinin and mesothelin increased the panel’s sensitivity from 64 to 73% at a predefined specificity of 97%. Circulating *GAS5* is not affected by pleurectomy before blood collection, age, or smoking status.

**Conclusions:**

*GAS5* is verified as an appropriate circulating marker for the supplement of calretinin and mesothelin to detect malignant mesothelioma. Although the sensitivity of *GAS5* is too low for the use as a single marker, the addition of *GAS5* as a third marker improves the performance of the established marker panel. The benefit of *GAS5* for the detection of malignant mesothelioma at early stages needs to be validated in a prospective study.

## Background

Malignant mesothelioma is an aggressive cancer caused by exposure to asbestos. The incidence is increasing worldwide and the global annual number of mesothelioma deaths was extrapolated to be 38,400 [1]. Commonly, mesothelioma is characterized by a long latency of up to 50 years and is usually diagnosed at late stages of the disease, resulting in poor survival between nine and 13 months, depending on treatment [2]. For the detection of mesothelioma at early stages non- or minimally-invasive methods, i.e. liquid biopsies, are preferable. Up to date, only the combination of the proteins calretinin and mesothelin has been validated for the early detection of mesothelioma using plasma samples taken up to 15 months before the clinical diagnosis [3]. At a fixed high specificity of 98% the marker combination revealed a sensitivity of 46%. However, for the further improvement of the panel performance additional markers are needed. These marker candidates could either be additional proteins or derived from different molecular classes, i.e. DNA and RNA. In general, proper markers of all molecular classes need to fulfill four key characteristics, namely detectability, robustness, sufficient sensitivity, and high specificity, for the detection of malignant diseases [4]. In recent years, non-coding RNAs have been in the focus of marker research. Particularly, long non-coding RNAs (lncRNAs) represent a versatile and promising group of potential markers, playing a role as oncogenes as well as tumor suppressors, and showing an altered expression in cancer [5, 6]. However, to the best of our knowledge, *RP1-86D1.3* is the only known circulating lncRNA for the detection of malignant mesothelioma so far, marked by a sensitivity of 83% and a specificity of 95% [7].

The aims of this study were (*i*) the identification of circulating lncRNAs as candidate markers for the detection of malignant mesothelioma using previously published RNA expression profiles from Gene Expression Omnibus (GEO), (*ii*) the verification using mesothelioma cell lines as well as human plasma samples from mesothelioma patients and subjects formerly exposed to asbestos as controls, and (*iii*) to assess the possible benefit of adding new candidate markers to the established marker panel of calretinin and mesothelin for the detection of malignant mesothelioma in liquid biopsies.

## Methods

### In silico analysis

RNA expression data of nine human pleural mesotheliomas and four normal pleural specimens using Affymetrix HGU133A plus 2.0 microarrays were obtained from GEO database (http://ncbi.nlm.hih.gov/projects/geo) as GSE 12345 series [8]. Raw CEL files were quantile normalized and background adjusted using RMAExpress Version 1.1.0 (http://rmaexpress.bmbolstad.com/). Using the annotated lncRNA transcripts with corresponding probe IDs on the HGU133A plus 2.0 microarray generated by Zhang et al. [9] Significance Analysis of Microarrays (SAM) version 5.0 (https://github.com/MikeJSeo/SAM) [10] was used with a false discovery rate (FDR) of < 10% and a fold change ≥1.5 to determine the differently expressed lncRNAs between pleural mesothelioma and normal human pleura.

### Cell lines

Four mesothelioma cell lines NCI-H2452 (ATCC® (American Type Culture Collection) CRL-5946™; LGC Standards GmbH, Wesel, Germany), NCI-H28 (ATCC® CRL-5820™; LGC Standards GmbH), JL-1 (ACC 596; Deutsche Sammlung von Mikroorganismen und Zellkulturen (DSMZ), Braunschweig, Germany), and MSTO-211H (ACC 390; DSMZ) were cultivated according to the supplier’s instructions. As epithelioid and biphasic mesothelioma are the predominated histological subtypes [11], corresponding cell lines were selected for the initial analysis. Harvested cells were resuspended in 500 μl RNAlater (Thermo Fisher Scientific, Darmstadt, Germany) and frozen at − 80 °C until use.

### Study population

The Molecular Marker (MoMar) cohort consists of 2769 German workers formerly exposed to asbestos with a confirmed asbestos-related disease, like asbestosis and/or other (non-malignant) pleural diseases but no malignancies at the beginning of the study participation.

Mesothelioma patients were recruited in participating medical practices of the MoMar study and at the Lungenklinik Heckeshorn, Helios Klinikum Emil von Behring, Berlin, Germany. The study group consisted of 22 male mesothelioma patients, including 14 (64%) epithelioid, two biphasic (9%), and two sarcomatoid (9%) mesotheliomas. The histological subtype for four cases (18%) remained unknown. Six patients underwent partial pleurectomy before blood drawing (median: 56 days, range: 24–179 days). Asbestos-exposed controls were derived from cancer-free participants of the MoMar study. The matched group consisted of 44 men formerly exposed to asbestos. Criteria for matching were age, smoking status, and time of blood collection. Characteristics of the study group are presented in Table 1.

**Table 1:** Characteristics of the study groups

	Mesothelioma patients (N)	Asbestos-exposed controls (N)
Gender		
Male	22	44
Age (years)		
Median	71.5	72.5
Range	40–84	49–83
Smoking status		
Ever	9	22
Never	11	22
Unknown	2	0
Histological subtype		
Epitheliod	14	–
Biphasic	2	–
Sacromatoid	2	–
Unknown	4	–

Additionally, nine subjects (four mesothelioma patients and five asbestos-exposed subjects) were recruited in the context of the MoMar study for initial experiments, but were not used in the performance analyses.

### Blood collection and storage

Peripheral blood of the subjects was collected in 9.0 ml S-Monovette EDTA gel tubes (Sarstedt, Nümbrecht, Germany). Blood samples were centrifugated at 2000x g for 10 min at room temperature within 30 min after collection. Afterwards, plasma was separated and temporarily stored frozen in the collaborating medical practices. Samples were transported frozen to the central laboratory, thawed at room temperature, aliquoted using an automated liquid handling robot (Tecan Group Ltd., Männedorf, Switzerland), and stored at − 80 °C until use.

### Isolation of RNA

RNA from cell lines was isolated using the miRVana miRNA Isolation Kit (Thermo Fisher Scientific, Darmstadt, Germany) according to the manufacturer’s instructions. Concentration of the isolated RNA was determined using the NanoDrop ND-100 spectrophotometer (Thermo Fisher Scientific). Isolated RNA of the immortalized human mesothelial cell line MeT-5A (ATCC® CRL-9444™) was purchased from tgcBIOMICS GmbH (Bingen, Germany).

RNA from 0.5 ml plasma samples was isolated using the miRVana PARIS kit (Thermo Fisher Scientific) according to the manufacturer’s instructions, modified by adding 5 μl carrier RNA MS2 (Roche, Mannheim, Germany). Amount of free hemoglobin (Hb) in plasma was measured by spectral analysis using a NanoDrop ND-100 spectrophotometer (Thermo Fisher Scientific). Absorbance was measured at 415 nm (total Hb), 450 nm (bilirubin), and 700 nm (sample turbidity). Hemoglobin concentrations were quantified using the formula Hb (mg/dl) = 154.7 x A_415_–130.7 x A_450_–123.9 x A_700_ [12, 13]. Hemoglobin values (g/l) of the plasma samples used in the subsequent performance analyses are presented in Additional File 3.

### Expression analysis

Expression analysis of lncRNAs and mRNAs was performed using a MJ Research PTC-200 Thermal Cycler (Bio-Rad Laboratories, Hercules, CA, USA) for reverse transcription (RT) and preamplification and a 7900 HT Fast Real-Time PCR System (Thermo Fisher Scientific) for quantitative real-time PCR (qPCR) according to the manufacturer’s instructions. In brief, using RNA from cell lines, RT was carried out in 25 μl reaction volume with 40 ng RNA as template. The subsequent qPCR was carried out in 20 μl reaction volume with 5 μl cDNA as template with and reactions were performed in duplicate. Using RNA from plasma samples, RT was carried out in 20 μl reaction volume with 5 μl RNA as template. Intermediary pre-amplification with 14 cycles was carried out in 10 μl reaction volume with 2.5 μl cDNA as template. For pre-amplification (but not for subsequent PCR) primers were diluted 1:100 according to the manufacturer’s instructions. Lastly, qPCR was carried out in 20 μl reaction volume with 5 μl DNA of a 1:20 dilution as template and reactions were performed in duplicate. Non-template controls were included in all assays. Estimation of the cycle threshold (Ct) was performed as described elsewhere [14]. The IDs of the commercially available probe-based assays (Integrated DNA Technologies, Leuven, Belgium) are presented in Additional File 1.

The candidate references *B2M*, *GUSB*, *HPRT1*, *PPIA*, *RPLP0*, and *TBP* [15] as well as the geometric mean (GM) of various reference combinations were analyzed using RefFinder to evaluate the most stable reference [16, 17]. Different lncRNA expressions in mesothelioma cell lines in comparison to MeT-5A were calculated using the 2^-ΔΔCt^ method [18]. Altered expressions of lncRNAs were considered as significant for fold changes < 0.5 and > 2.0 [19]. Raw Ct values of lncRNAs and mRNAs in cell lines are presented in Additional File 2. Assays were analyzed to avoid hetero-dimers using the OligoAnalyzer tool (Integrated DNA Technologies). For group comparison between mesothelioma cases and asbestos-exposed controls marker values in plasma were normalized and expressed as 2^-ΔCt^. Raw Ct values in plasma ≥35 were considered to be under the detection limit and samples were excluded from further analyses. Raw Ct values of markers and references in the plasma samples used in the subsequent performance analyses are presented in Additional File 3.

### Determination of calretinin and mesothelin

Enzyme-linked immunosorbent assays (ELISA) were used for the determination of calretinin and mesothelin in plasma. For calretinin the Calretinin ELISA kit (DLD Diagnostika GmbH, Hamburg, Germany) was used according to the manufacturer’s instructions. For mesothelin the Mesomark ELISA Kit (Fujirebio Diagnostics, Inc., Malvern, PA, USA) was used according to the manufacturer’s instructions with modifications as described elsewhere [20]. All samples were determined in duplicate. Optical densities were measured using a SpectraMax 384 plus plate reader (Molecular Devices, Sunnyvale, CA, USA) and the standard curves were obtained by four-parameter curve fitting using the SoftMax Pro 5.4.1 software (Molecular Devices). Values of calretinin and mesothelin in the plasma samples used in the subsequent performance analyses are presented in Additional File 3.

### Statistical analyses

Box plots with median and inter-quartile range (IQR) were used to describe the distribution of marker concentrations. Whiskers depicted minimum and maximum. Mesothelioma cases and cancer-free controls were compared using the non-parametric Kruskal-Wallis test for continuous variables. Classification performances of *GAS5*, calretinin, and mesothelin were determined from receiver operating characteristic (ROC) curves. The accuracy of the marker performances was depicted by the area under the curve (AUC) and its 95% confidence interval (95% CI). The markers were combined in two different ways. For linear marker combination, ROC curves were calculated with the corresponding markers as independent variables in a multiple logistic regression model. Sequential combination was performed as described elsewhere [3]. In brief, the first marker was used for classification. Afterwards, only marker-negative subjects were examined with the next marker and so on. This procedure was repeated for all possible cut points to calculate the related sensitivities and specificities. The related AUC intervals depict minimum and maximum obtainable AUCs. Potential factors influencing *GAS5* concentration were evaluated using a multiple linear regression model with log-transformed marker values. Estimates were given as Exp(β) with 95% CI and *p*-values. Here, values of Exp(β) > 1 indicate a positive and Exp(β) < 1 indicate a negative association between analyzed factor and *GAS5*. Statistical analyses were performed using SAS/STAT and SAS/IML software, version 9.4 (SAS Institute Inc., Cary, NC, USA). GraphPad Prism version 7.04 (GraphPad Software, La Jolla California, USA) was used to prepare graphs.

## Results

### In silico lncRNA expression analysis

The Affymetrix HG-U133 Plus 2.0 arrays include 2448 probe sets representing 1988 lncRNAs (Additional File 4). Analysis of the lncRNA expression between pleural mesothelioma cases and normal pleural controls identified 40 altered lncRNAs, of which 28 lncRNAs were up-regulated and twelve lncRNAs were down-regulated in mesothelioma (Table 2).

**Table 2:** Differently expressed lncRNAs between pleural mesothelioma cases (*N* = 9) and normal pleural controls (*N* = 4)

Regulation	lncRNAs
up	*AFAP1-AS*^‡^, *C17orf69*^‡^, CRNDE*, *CTB-89H12.4*^§^, DLEU2*, *FLJ22536*^‡^, GAS5*, HCG18*, *JPX*^§^, MALAT1*, MEG3*, NEAT1*, *LOC84856*^‡^, *LOC84989*^‡^, *LOC388796*^‡^, *LOC401504*^‡^, *LOC440944*^‡^, *LOC642852*^‡^, *LOC100129196*^‡^, *LOC100130776*^‡^, *LOC1001302275*^‡^, NCRNA00201*, *NCRNA00230A/NCRNA00230B*^‡^, *NCRNA00275*^‡^, PVT1*, *RP1-224A6.6*^§^, *RP11-112 J3.16*^§^, *RP11-322 M19.1*^§^
down	*AC112217.2*^§^, *AL035610.1*^§^, C14orf139*, *CTD-2230D16.1*^§^, *EMX2OS*^‡^, *HYMAI*^‡^, *LOC387723*^‡^, *LOC400043*^‡^, *LOC572558*^‡^, *LOH3CR2A*^‡^, *MGC16275*^‡^, *RP1-28O10.1*^§^

*Annotation by Refseq and Ensembl databases. ^‡^Annotation by Refseq database. ^§^Annotation by Ensembl database

### Assessment of lncRNA detectability

Candidate references were determined in NCI-H2452, NCI-H28, JL-1, MSTO-211H, and MeT-5A. Using RefFinder the GM of *B2M*, *HPRT1*, and *RPLP0* was identified as the most stable reference for the normalization of lncRNAs in the analyzed cell lines.

Twenty-four lncRNAs initially identified in silico were determined in the cell lines as candidate markers. Using the 2^-ΔΔCt^ method to assess different expressions between mesothelioma cell lines and MeT-5A as control revealed an up-regulation of *AFAP1-AS1*, *GAS5*, and *LOC84856* in at least three of the mesothelioma cell lines. *LOC642852* and *LOC388796* showed a constant down-regulation in all cell lines, whereas *CRNDE* and *LOC100130776* showed no altered regulation in mesothelioma cell lines. The remaining lncRNAs showed sporadically up- and down-regulation in the various cell lines (Table 3). All other lncRNAs were constantly not detectable in the cell lines.

**Table 3:** Fold change of long non-coding (lncRNAs) in mesothelioma cell lines. Fold changes > 2.0 represent an up-regulation and fold changes < 1.5 a down-regulation

lncRNA	Cell line			
	NCI-H28	NCI-H2452	JL-1	MSTO-211H
*AFAP1-AS1*	0.88	54.32	1025.09	522.48
*GAS5*	4.08	1.19	8.52	2.64
*CRNDE*	1.53	1.12	0.89	1.87
*LOC642852*	0.09	0.16	0.13	0.07
*LOC84856*	7.71	5.44	3.77	2.69
*LOC388796*	0.07	0.06	0.17	0.05
*NCRNA00201*	8.89	1.61	1.63	0.97
*LOC100130776*	1.96	1.18	1.34	1.25
*LOC401504*	2.23	0.30	1.55	0.61
*PVT1*	1.57	1.07	1.68	12.41
*HCG18*	1.36	0.48	0.94	0.20

Afterwards, the general detectability of the lncRNAs in liquid biopsies was assessed using nine plasma samples from four mesothelioma patients and five subjects formerly exposed to asbestos (Additional file 5). *GAS5* was detectable in almost all plasma samples, in contrast to all other analyzed lncRNAs. Based on the obtained results, circulating GAS5 was selected as candidate marker for further analyses.

### Circulating *GAS5* as marker for mesothelioma

Potential references were measured in the plasma samples of the study group. Using raw Ct values statistically significant differences were revealed for *PPIA* (*p* < 0.001) and *B2M* (p < 0.001). No differences could be observed for *RPLP0* (*p* = 0.516) and *HPRT1* (*p* = 0.285), but *HPRT1* was detectable only in 32 of 53 samples (60.4%). Additionally, using RefFinder *RPLP0* was identified as the most stable reference. Thus, *RPLP0* was selected for the normalization of circulating *GAS5*.

**Fig. 1:**
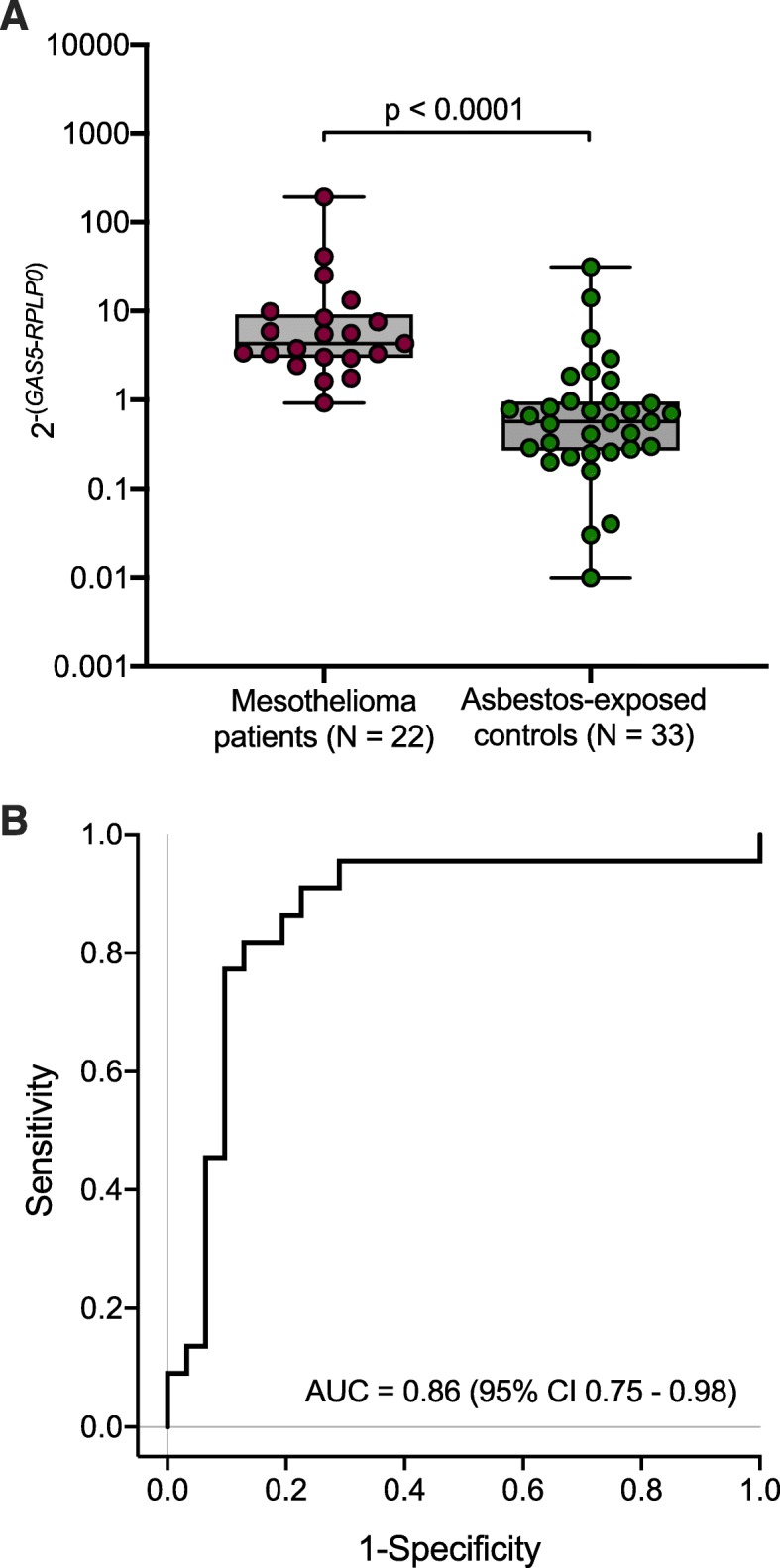
**a** Distribution of normalized *GAS5* in plasma of mesothelioma patients and asbestos-exposed controls. **b** Receiver operating characteristic (ROC) curve of circulating GAS5

Eleven asbestos-exposed controls were excluded from analyses because raw Ct values of *GAS5* or *RPLP0* in plasma were ≥ 35, resulted in 22 mesothelioma patients and 31 asbestos-exposed controls appropriate for analysis. The median plasma level of normalized *GAS5* was 4.05 (IQR 2.94–8.38) in mesothelioma patients and 0.62 (IQR 0.28–0.96) in asbestos-exposed controls (Fig. 1a). The difference of circulating *GAS5* in plasma between mesothelioma patients and asbestos-exposed controls was statistically significant (*p* < 0.0001). Using ROC analysis an AUC of 0.86 (95% CI 0.75–0.98) was calculated for circulating *GAS5* (Fig. 1b).

Using a predefined high specificity of 97%, allowing one false-positive test, resulted in 14% sensitivity for circulating *GAS5* in plasma (Table 4).

**Table 4:** Performance of *GAS5*, calretinin, mesothelin, linear combination, and sequential combination of the markers

	*GAS5*	Calretinin	Mesothelin	Calretinin and mesothelin	*GAS5*, calretinin, and mesothelin	Mesothelin, *GAS5*, and calretinin
Combination	–	–	–	Sequential	Linear	Sequential
N	53	53	52	52	52	52
AUC	0.86	0.84	0.75	0.88	0.88	0.96
	(95% CI 0.75–0.98)	(95% CI 0.74–0.96)	(95% CI 0.61–0.89)	(range 0.76–0.88)	(95% CI 0.78–0.99)	(range 0.85–0.96)
Cut-off	25.496	1.044	2.861	1.044 and 3.147	–	3.147, 25.496, and 1.305
Sensitivity (%)	14	55	41	64	73	68
Specificity (%)	97	97	97	97	97	97
True-positive tests (N)	3	12	9	14	16	15
True-negative tests (N)	30	30	29	29	29	29
False-positive tests (N)	1	1	1	1	1	1
False-negative tests (N)	19	10	13	8	6	7

The impact of influencing factors on circulating *GAS5* in plasma was analyzed in the study group. Pleurectomy before blood collection, age, and smoking status did not influence the *GAS5* levels in plasma, whereas the target disease leads to increased values (Table 5).

**Table 5:** Estimates of the influence of potential factors on *GAS5* in plasma

	*GAS5*			
	N	Exp (ß)	95% CI	*p*-value
Intercept		0.05	0.00–1.28	
Mesothelioma vs. asbestos-exposed controls	53	6.62	2.52–17.43	0.0003
Pleurectomy before blood collection	50	0.67	0.13–3.36	0.6220
Age (years)	53	1.05	1.00–1.09	0.0511
Ever vs. never smokers	51	0.44	0.18–1.06	0.0660

### Determination of calretinin and mesothelin

**Fig. 2:**
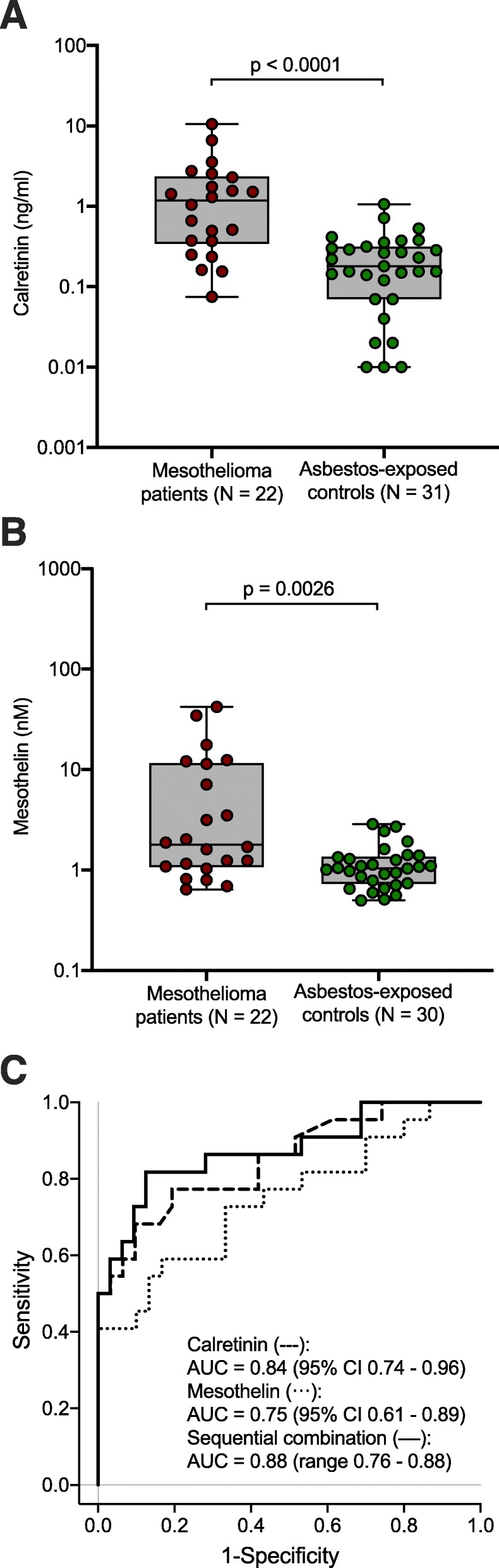
**a** Distribution of calretinin in plasma of mesothelioma patients and asbestos-exposed controls. **b** Distribution of mesothelin in plasma of mesothelioma patients and asbestos-exposed controls. **c** Receiver operating characteristics (ROC) curves of calretinin, mesothelin, and sequential combination of calretinin and mesothelin

The median calretinin value in mesothelioma patients was 1.17 (IQR 0.37–2.29) and in asbestos-exposed controls 0.18 (IQR 0.07–0.32). The median mesothelin level was 1.79 (IQR 1.08–11.41) in mesothelioma patients and 1.04 (IQR 0.74–1.34) in asbestos-exposed controls (Fig. 2a and b). Differences were statistically significant for calretinin (*p* < 0.0001) and mesothelin (*p* = 0.0026). Using ROC analyses, AUCs of 0.84 (95% CI 0.74–0.96), 0.75 (95% CI 0.61–0.89), and 0.88 (range 0.76–0.88) were calculated for calretinin, mesothelin, and the sequential combination of both markers, respectively (Fig. 2c). Using a predefined specificity of 97% revealed sensitivities of 55% for calretinin, 41% for mesothelin, and 64% for the sequential combination of calretinin and mesothelin (Table 4).

### Combination of *GAS5* with calretinin and mesothelin

**Fig. 3:**
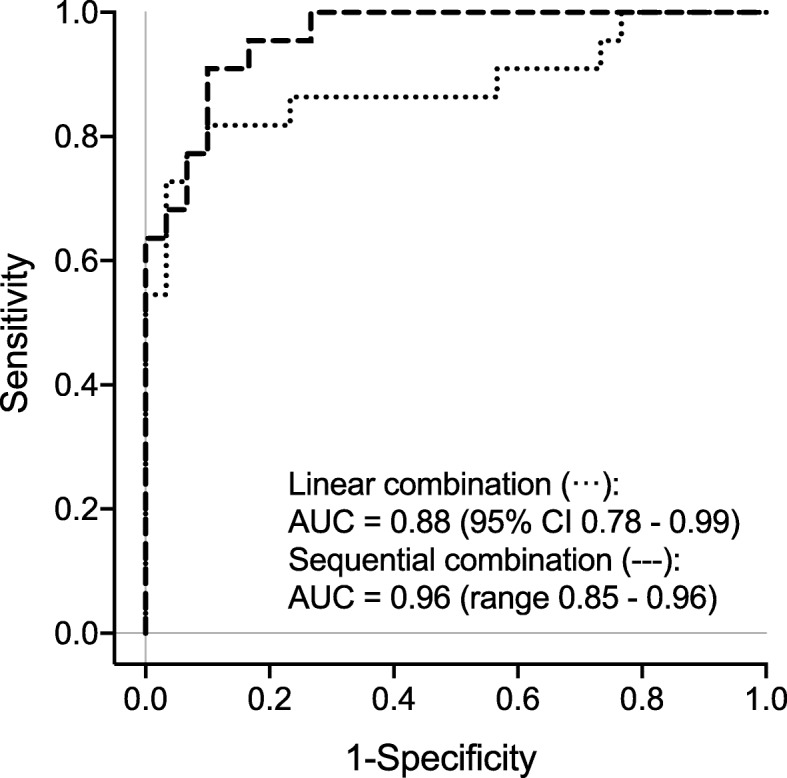
Receiver operating characteristics (ROC) curves of linear and sequential combination using *GAS5*, calretinin, and mesothelin

The combination of *GAS5*, calretinin, and mesothelin was evaluated using a linear and a sequential approach. The ROC curves revealed an AUC of 0.88 (95% CI 0.78–0.99) for the linear combination and 0.96 (range 0.85–0.96) for the sequential combination (Fig. 3). Using a predefined specificity of 97% resulted in 73% sensitivity for the linear combination and 68% sensitivity for the sequential combination (Table 4).

## Discussion

Altered expression of lncRNAs were shown in multiple human cancers [21] and the number of lncRNAs surpasses the number of protein coding genes, i.e. approximately 60,000 lncRNAs vs. 30,000 protein coding genes [22]. Thus, it is indicated that the large pool of lncRNAs includes a greater number of potential marker candidates for the detection of cancer.

The Affymetrix Gene Chip Human Genome U133 is one of the most frequently used microarray for human cancer profiling [9] and expression data are deposited in public gene expression repositories, e.g. GEO. The re-annotation and the classification pipeline according to Zhang et al. allows the identification and the expression analysis of lncRNAs using expression data sets from analyses primarily targeting mRNAs [9]. In this study, the in silico analysis of the lncRNA expression using pleural mesothelioma and normal human pleura revealed 40 altered lncRNAs. Notably, *RP1-86D1.3* [7] was not identified as differently expressed lncRNA. Additionally, Wright et al. detected 33 lncRNAs differentially expressed in mesothelioma cell lines compared to MeT-5A [23], but only *NEAT1* was identified in both studies. Such differences might rely on different microarrays and samples types as well as relatively small numbers of analyzed samples. However, in previous analysis using plasma samples we could not confirm *NEAT1* as circulating marker for mesothelioma (data not shown). Thus, *NEAT1* was not further analyzed in the current study.

In this study, only *GAS5* was reliably proven as a marker for the detection of mesothelioma using liquid biopsies. *GAS5* is located on chromosome 1q25, comprising twelve exons and playing an important role in carcinogenesis as a tumor suppressor [24]. We found a significant up-regulation of *GAS5* in plasma of mesothelioma patients and mesothelioma cell lines, confirming our initial in silico analysis of published expression data based on tissue samples of pleural mesothelioma. This agrees with the up-regulation of *GAS5* in mesothelioma tissues shown by Renganathan et al., although in the same study a down-regulation of *GAS5* was observed using primary mesothelioma cell cultures [25]. Such divergent results were also obtained for the lncRNA *MALAT1*, reflecting that in spite of its ubiquitous expression *MALAT1* could function in a cell type- or tissue-specific manner [26], and the same might be true for *GAS5*. Accordingly, despite the described predominant role as tumor suppressor, it appears that *GAS5* might also act as an oncogene, not only in mesothelioma but also in other malignancies, e.g. prostate and esophageal cancer [27, 28]. However, the results show unambiguously that *GAS5* fulfills the first key characteristic of a diagnostic marker: to be detectable in liquid biopsies. Additionally, the observed up-regulation of *GAS5* in mesothelioma tissues as well as in plasma of mesothelioma patients suggested that the presence of circulating *GAS5* in plasma might be a direct effect of the tumor, e.g., via the secretion of lncRNA containing extracellular vesicles [29]. To the best of our knowledge, this is the first study using circulating *GAS5* as a diagnostic marker for the detection of malignant mesothelioma using liquid biopsies. Kresoja-Rakic et al. determined *GAS5* as prognostic marker using plasma samples from mesothelioma patients before and after chemotherapy [30]. Otherwise, circulating *GAS5* is repeatedly suggested as a marker for the detection of lung cancer, showing a consistent down-regulation in cancer patients [31–33].

Regarding the key characteristics of a sufficient sensitivity and a high specificity, the sensitivity of candidate markers should be calculated at a fixed high specificity [34]. Generally, high specificity is needed to avoid false-positive tests, resulting in psychological stress, overdiagnosis, and needless interventions for the affected. Due to the relatively small number of analyzed subjects only a single false-positive test was allowed in this study, representing a predefined specificity of 97%. This resulted in a low sensitivity of 14% for *GAS5* as a single marker. However, lower sensitivity could be balanced by the use of various markers in a panel. In theory, in an optimal panel every marker is characterized by sufficient sensitivity and the necessary high specificity, complementing each other to obtain superior diagnostic performance [35]. The potential of calretinin and mesothelin to discriminate between mesothelioma cases and asbestos-exposed controls has been confirmed in various studies [36–39] and recently, both markers have also been validated for the early detection of malignant mesothelioma [3]. Using a linear approach *GAS5* was combined with calretinin and mesothelin. At a predefined specificity of 97%, the AUC of this combination decreased slightly, but the sensitivity increased from 64 to 73%, resulting in two additional true-positive tests in comparison to the combination of calretinin and mesothelin alone. Using a sequential combination of the three markers revealed a higher AUC, but at the predefined specificity the sensitivity is 68%, resulting in only one additional true-positive test. The results indicate that *GAS5* might be useful as a complementary marker for the established marker combination. Because the combination of calretinin and mesothelin already works well, the performance of the panel is improved only slightly by the additional marker. Further improvements will most likely require a larger number of markers. It should be noted, however, that the current as well as previous studies analyzed marker combinations with diagnosed cases at mostly late stages of tumor development. As has been shown before, using prediagnostic samples of mesothelioma cases can result in a better performance regarding marker complementation [3].

Considering the forth key characteristic, *GAS5* seems to be relatively robust regarding obvious influencing factors. However, this assumption needs to be verified in more detail using larger study groups. Additionally, it was shown that hemolysis influence microRNA levels in plasma [40] and the same might be true for lncRNAs [41]. Thus, free hemoglobin was determined in all plasma samples, showing that no hemoglobin value exceeding the clinically significant threshold (> 0.3 g/l) [42]. However, the real impact of the hemolysis grade on lncRNAs levels should be analyzed in more detail in appropriate studies, e.g. using artificial hemolysis [43].

The results of this study are based on small numbers. Thus, it might be meaningful to verify *GAS5* in a larger and independent study group. Additionally, it is known that earlier detection of cancer can improve survival, at least for some cancer types [44]. Thus, for early diagnosis it will also be necessary to validate *GAS5* as well as the previously identified *RP1-86D1.3* [7] in a prospective study regarding their potential to detect mesothelioma in prediagnostic plasma samples and to complement calretinin and mesothelin. This validation procedure is an obligatory step to select appropriate candidate markers for early detection. This is exemplified by some promising candidate markers, i.e. miR-103a-3p, miR-132-3p, and 126-3p, that were identified in common case-control studies but ultimately failed to detect mesothelioma in prediagnostic samples [45]. Therefore, more marker candidates - preferably of all molecular classes - need to be identified and validated for the completion of a useful and reliable marker panel to detect malignant mesothelioma at early stages.

## Conclusions

*GAS5* was identified in silico and verified in cell lines as well as human liquid biopsies as an appropriate circulating marker for the supplement of calretinin and mesothelin to detect malignant mesothelioma. Although the sensitivity of *GAS5* is too low for the use as a single marker, the addition of *GAS5* as a third marker improves the performance of the established marker panel. The benefit of *GAS5* for the detection of mesothelioma at early stages using plasma samples taken before clinical diagnosis still needs to be validated in a prospective study.

## Supplementary information

**Additional file 1.** IDs of the commercial probe-based assays purchased by Integrated DNA Technologies (IDT).

**Additional file 2.** Raw Ct values of the long non-coding RNAs (lncRNAs) and messenger RNAs (mRNAs) in the analyzed cell lines.

**Additional file 3 **Detailed characteristics of the individuals used in the performance analyses and corresponding levels of hemoglobin (ng/ml), *GAS5* (Ct), *RPLP0* (Ct), calretinin (ng/ml), and mesothelin (nM).

**Additional file 4.** Long non-coding RNAs (lncRNAs) represented on the Affymetrix HG-U133 Plus 2.0 arrays.

**Additional file 5.** Raw Ct values of long non-coding RNAs (lncRNAs) in plasma samples.

## Data Availability

All data generated or analyzed during this study are included within this published article and its supplementary information files.

## Abbreviations

ATCC: American Type Culture Collection
AUC: Are under curve
cDNA: Complementary DNA
95% CI: 95% confidence interval
Ct: Cycle threshold
DNA: Deoxyribonucleic acid
DSMZ: Deutsche Sammlung von Mikroorganismen und Zellkulturen
ELISA: Enzyme-linked immunosorbent assays
FDR: False discovery rate
*GAS5*: Growth arrest-specific transcript 5
GEO: Gene Expression Omnibus
GM: Geometric mean
Hb: Hemoglobin
IQR: Interquartile range
lncRNA: Long non-coding RNA
mRNA: Messenger RNA
PCR: Polymerase chain reaction
qPCR: Quantitative PCR
RNA: Ribonucleic acid
ROC: Receiver operating characteristics
RT: Reverse transcription
SAM: Significance Analysis of Microarrays

## References

[1] Odgerel CO, Takahashi K, Sorahan T, Driscoll T, Fitzmaurice C, Yoko OM (2017). Estimation of the global burden of mesothelioma deaths from incomplete national mortality data. Occup Environ Med.

[2] Hodgson JT, McElvenny DM, Darnton AJ, Price MJ, Peto J (2005). The expected burden of mesothelioma mortality in Great Britain from 2002 to 2050. Br J Cancer.

[3] Johnen G, Burek K, Raiko I, Wichert K, Pesch B, Weber DG (2018). Prediagnostic detection of mesothelioma by circulating calretinin and mesothelin - a case-control comparison nested into a prospective cohort of asbestos-exposed workers. Sci Rep.

[4] Pesch B, Bruning T, Johnen G, Casjens S, Bonberg N, Taeger D (2014). Biomarker research with prospective study designs for the early detection of cancer. Biochim Biophys Acta.

[5] Reis EM, Verjovski-Almeida S (2012). Perspectives of long non-coding RNAs in Cancer diagnostics. Front Genet.

[6] Pardini B, Sabo AA, Birolo G, Calin GA. Noncoding RNAs in extracellular fluids as cancer biomarkers: the new frontier of liquid biopsies. Cancers (Basel). 2019;11(8):E1170. 10.3390/cancers11081170 PMC6721601 31416190

[7] Matboli M, Shafei AE, Ali MA, Gaber AI, Galal A, Tarek O (2019). Clinical significance of serum DRAM1 mRNA, ARSA mRNA, hsa-miR-2053 and lncRNA-RP1-86D1.3 axis expression in malignant pleural mesothelioma. J Cell Biochem.

[8] Crispi S, Calogero RA, Santini M, Mellone P, Vincenzi B, Citro G (2009). Global gene expression profiling of human pleural mesotheliomas: identification of matrix metalloproteinase 14 (MMP-14) as potential tumour target. PLoS One.

[9] Zhang X, Sun S, Pu JK, Tsang AC, Lee D, Man VO (2012). Long non-coding RNA expression profiles predict clinical phenotypes in glioma. Neurobiol Dis.

[10] Tusher VG, Tibshirani R, Chu G (2001). Significance analysis of microarrays applied to the ionizing radiation response. Proc Natl Acad Sci U S A.

[11] Neumann V, Gunthe S, Mulle KM, Fischer M (2001). Malignant mesothelioma--German mesothelioma register 1987-1999. Int Arch Occup Environ Health.

[12] Tolan NV, Vidal-Folch N, Algeciras-Schimnich A, Singh RJ, Grebe SK (2013). Individualized correction of neuron-specific enolase (NSE) measurement in hemolyzed serum samples. Clinica chimica acta; international journal of clinical chemistry.

[13] Fairbanks VF, Ziesmer SC, O'Brien PC (1992). Methods for measuring plasma hemoglobin in micromolar concentration compared. Clin Chem.

[14] Weber DG, Johnen G, Casjens S, Bryk O, Pesch B, Jockel KH (2013). Evaluation of long noncoding RNA MALAT1 as a candidate blood-based biomarker for the diagnosis of non-small cell lung cancer. BMC Res Notes..

[15] Melaiu O, Melissari E, Mutti L, Bracci E, De Santi C, Iofrida C (2015). Expression status of candidate genes in mesothelioma tissues and cell lines. Mutat Res.

[16] Xie F, Xiao P, Chen D, Xu L, Zhang B (2012). miRDeepFinder: a miRNA analysis tool for deep sequencing of plant small RNAs. Plant Mol Biol.

[17] Vandesompele J, De Preter K, Pattyn F, Poppe B, Van Roy N, De Paepe A (2002). Accurate normalization of real-time quantitative RT-PCR data by geometric averaging of multiple internal control genes. Genome Biol.

[18] Livak KJ, Schmittgen TD (2001). Analysis of relative gene expression data using real-time quantitative PCR and the 2(−Delta Delta C(T)) method. Methods..

[19] Mattie MD, Benz CC, Bowers J, Sensinger K, Wong L, Scott GK (2006). Optimized high-throughput microRNA expression profiling provides novel biomarker assessment of clinical prostate and breast cancer biopsies. Mol Cancer.

[20] Weber DG, Taeger D, Pesch B, Kraus T, Bruning T, Johnen G (2007). Soluble mesothelin-related peptides (SMRP) - high stability of a potential tumor marker for mesothelioma. Cancer Biomark.

[21] Gibb EA, Vucic EA, Enfield KS, Stewart GL, Lonergan KM, Kennett JY (2011). Human cancer long non-coding RNA transcriptomes. PLoS One.

[22] Iyer MK, Niknafs YS, Malik R, Singhal U, Sahu A, Hosono Y (2015). The landscape of long noncoding RNAs in the human transcriptome. Nat Genet.

[23] Wright CM, Kirschner MB, Cheng YY, O'Byrne KJ, Gray SG, Schelch K (2013). Long non coding RNAs (lncRNAs) are dysregulated in malignant pleural mesothelioma (MPM). PLoS One.

[24] Ji J, Dai X, Yeung SJ, He X (2019). The role of long non-coding RNA GAS5 in cancers. Cancer Manag Res.

[25] Renganathan A, Kresoja-Rakic J, Echeverry N, Ziltener G, Vrugt B, Opitz I (2014). GAS5 long non-coding RNA in malignant pleural mesothelioma. Mol Cancer.

[26] Eissmann M, Gutschner T, Hammerle M, Gunther S, Caudron-Herger M, Gross M (2012). Loss of the abundant nuclear non-coding RNA MALAT1 is compatible with life and development. RNA Biol.

[27] Li W, Zhao W, Lu Z, Zhang W, Yang X (2018). Long noncoding RNA GAS5 promotes proliferation, migration, and invasion by regulation of miR-301a in esophageal Cancer. Oncol Res.

[28] Zhang Y, Su X, Kong Z, Fu F, Zhang P, Wang D (2017). An androgen reduced transcript of LncRNA GAS5 promoted prostate cancer proliferation. PLoS One.

[29] Kogure T, Yan IK, Lin WL, Patel T (2013). Extracellular vesicle-mediated transfer of a novel long noncoding RNA TUC339: a mechanism of intercellular signaling in human hepatocellular Cancer. Genes & cancer.

[30] Kresoja-Rakic J, Szpechcinski A, Kirschner MB, Ronner M, Minatel B, Martinez VD, et al. miR-625-3p and lncRNA GAS5 in liquid biopsies for predicting the outcome of malignant pleural mesothelioma patients treated with neo-adjuvant chemotherapy and surgery. Noncoding RNA. 2019;5(2). 10.3390/ncrna5020041 PMC6631473 31212997

[31] Liang W, Lv T, Shi X, Liu H, Zhu Q, Zeng J (2016). Circulating long noncoding RNA GAS5 is a novel biomarker for the diagnosis of nonsmall cell lung cancer. Medicine (Baltimore).

[32] Kamel LM, Atef DM, Mackawy AMH, Shalaby SM, Abdelraheim N (2019). Circulating long non-coding RNA GAS5 and SOX2OT as potential biomarkers for diagnosis and prognosis of non-small cell lung cancer. Biotechnol Appl Biochem.

[33] Li C, Lv Y, Shao C, Chen C, Zhang T, Wei Y (2019). Tumor-derived exosomal lncRNA GAS5 as a biomarker for early-stage non-small-cell lung cancer diagnosis. J Cell Physiol.

[34] Zhu CS, Pinsky PF, Cramer DW, Ransohoff DF, Hartge P, Pfeiffer RM (2011). A framework for evaluating biomarkers for early detection: validation of biomarker panels for ovarian cancer. Cancer Prev Res.

[35] Mai PL, Wentzensen N, Greene MH (2011). Challenges related to developing serum-based biomarkers for early ovarian cancer detection. Cancer Prev Res.

[36] Johnen G, Gawrych K, Raiko I, Casjens S, Pesch B, Weber DG (2017). Calretinin as a blood-based biomarker for mesothelioma. BMC Cancer.

[37] Aguilar-Madrid G, Pesch B, Calderon-Aranda ES, Burek K, Jimenez-Ramirez C, Juarez-Perez CA (2018). Biomarkers for predicting malignant pleural mesothelioma in a Mexican population. Int J Med Sci.

[38] Jimenez-Ramirez C, Casjens S, Juarez-Perez CA, Raiko I, Del Razo LM, Taeger D (2019). Mesothelin, Calretinin, and megakaryocyte potentiating factor as biomarkers of malignant pleural mesothelioma. Lung..

[39] Cui A, Jin XG, Zhai K, Tong ZH, Shi HZ (2014). Diagnostic values of soluble mesothelin-related peptides for malignant pleural mesothelioma: updated meta-analysis. BMJ Open.

[40] Kirschner MB, Kao SC, Edelman JJ, Armstrong NJ, Vallely MP, van Zandwijk N (2011). Haemolysis during sample preparation alters microRNA content of plasma. PLoS One.

[41] Alvarez-Dominguez JR, Hu W, Yuan B, Shi J, Park SS, Gromatzky AA (2014). Global discovery of erythroid long noncoding RNAs reveals novel regulators of red cell maturation. Blood..

[42] Lippi G, Plebani M, Di Somma S, Cervellin G (2011). Hemolyzed specimens: a major challenge for emergency departments and clinical laboratories. Crit Rev Clin Lab Sci.

[43] Weber DG, Gawrych K, Casjens S, Brik A, Lehnert M, Taeger D (2017). Circulating miR-132-3p as a candidate diagnostic biomarker for malignant mesothelioma. Dis Markers.

[44] Early detection: a long road ahead. Nat Rev Cancer. 2018;18(7):401. 10.1038/s41568-018-0021-8 29769632

[45] Weber DG, Brik A, Casjens S, Burek K, Lehnert M, Pesch B (2019). Are circulating microRNAs suitable for the early detection of malignant mesothelioma? Results from a nested case-control study. BMC Res Notes.

