# Supplementary material for: Circulating long non-coding RNA GAS5 (growth arrest-specific transcript 5) as a complement marker for the detection of malignant mesothelioma using liquid biopsies
Source: Biomark Res. 2020 May 13;8:15. doi: 10.1186/s40364-020-00194-4 (PMC7222324; doi:10.1186/s40364-020-00194-4)
Supplement: Supplementary file 1 — Additional file 1. IDs of the commercial probe-based assays purchased by Integrated DNA Technologies (IDT). [file 40364_2020_194_MOESM1_ESM.docx]

**Additional file 1.** IDs of the commercial probe-based assays purchased by Integrated DNA Technologies (IDT).

| **lncRNA/mRNA** | **Assay ID (IDT)** |
| --- | --- |
| *AFAP1-AS* | Hs.PT.58.24705644 |
| *MEG3* | Hs.PT.58.40467125 |
| *GAS5* | Hs.PT.58.24767969 |
| *CRNDE* | Hs.PT.58.25672118 |
| *DLEU2* | Hs.PT.58.148382 |
| *LOC642852* | Hs.PT.58.15063168 |
| *LOC84856* | Hs.PT.58.15519221 |
| *LOC388796* | Hs.PT.58.524291 |
| *NCRNA00201* | Hs.PT.58.3736590 |
| *LOC440944* | Hs.PT.58.15426896 |
| *LOC100130776* | Hs.PT.58.3300843 |
| *LOC401504* | Hs.PT.58.24603146 |
| *PVT1* | Hs.PT.58.24584277 |
| *C17orf69 (1-2b)* | Hs.PT.58.40235058 |
| *C17orf69 (2a-3)* | Hs.PT.58.15037653 |
| *NCRNA00183* | Hs.PT.58.27247965 |
| *LOC100130275* | Hs.PT.58.15731817 |
| *LOC100129196* | Hs.PT.58.15026759 |
| *FLJ22536* | Hs.PT.58.3137542 |
| *HCG18* | Hs.PT.58.15260735 |
| *MGC16275* | Hs.PT.58.4202978 |
| *LOC387723* | Hs.PT.58.14945393 |
| *EMX2OS* | Hs.PT.58.24621090 |
| *LOC400043* | Hs.PT.58.3346389 |
| *HYMAI* | Hs.PT.58.25312231 |
| *B2M* | Hs.PT.58v.18759587 |
| *GUSB* | Hs.PT.58.38438455.gs |
| *HPRT1* | Hs.PT.39a.22214821 |
| *PPIA* | Hs.PT.39a.2214581 |
| *RPLP0* | Hs.PT.39a.22214824 |
| *TBP* | Hs.PT.58.22532795 |
