# Supplementary material for: Circulating long non-coding RNA GAS5 (growth arrest-specific transcript 5) as a complement marker for the detection of malignant mesothelioma using liquid biopsies
Source: Biomark Res. 2020 May 13;8:15. doi: 10.1186/s40364-020-00194-4 (PMC7222324; doi:10.1186/s40364-020-00194-4)
Supplement: Supplementary file 2 — Additional file 2. Raw Ct values of the long non-coding RNAs (lncRNAs) and messenger RNAs (mRNAs) in the analyzed cell lines. [file 40364_2020_194_MOESM2_ESM.docx]

**Additional file 2.** Raw Ct values of the long non-coding RNAs (lncRNAs) and messenger RNAs (mRNAs) in the analyzed cell lines.

| **lncRNA/mRNA** | **Cell lines** | | | | |
| --- | --- | --- | --- | --- | --- |
|  | **NCI-H2452** | **NCI-H28** | **JL-1** | **MSTO-211H** | **Met-5A** |
| *AFAP1-AS1* | 34,81 | 40,00 | 28,57 | 29,54 | 40,00 |
| *MEG3* | 40,00 | 40,00 | 32,85 | 34,03 | 39,58 |
| *GAS5* | 28,13 | 25,60 | 23,30 | 24,99 | 27,82 |
| *CRNDE* | 31,96 | 30,75 | 30,30 | 29,23 | 31,55 |
| *DLEU2* | 35,19 | 34,91 | 34,99 | 35,74 | 35,25 |
| *LOC642852* | 30,05 | 30,15 | 28,36 | 29,24 | 26,88 |
| *LOC84856* | 30,01 | 28,74 | 28,54 | 29,03 | 31,88 |
| *LOC388796* | 32,00 | 31,10 | 28,57 | 30,28 | 27,47 |
| *NCRNA00201* | 33,84 | 30,62 | 31,83 | 32,57 | 33,96 |
| *LOC440944* | 39,13 | 40,00 | 40,00 | 37,03 | 35,79 |
| *LOC100130776* | 29,99 | 28,49 | 27,81 | 27,90 | 29,65 |
| *LOC401504* | 30,23 | 26,57 | 25,86 | 27,19 | 27,92 |
| *PVT1* | 32,02 | 30,70 | 29,37 | 26,48 | 31,54 |
| *C17orf69 (1-2b)* | 38,59 | 33,05 | 37,42 | 34,86 | 38,90 |
| *C17orf69 (2a-3)* | 40,00 | 39,87 | 39,33 | 37,78 | 40,00 |
| *NCRNA00183* | 39,27 | 37,71 | 37,04 | 38,24 | 35,59 |
| *LOC100130275* | 40,00 | 36,41 | 39,79 | 37,24 | 37,83 |
| *LOC100129196* | 35,37 | 37,04 | 35,82 | 36,09 | 34,25 |
| *FLJ22536* | 36,66 | 34,43 | 34,80 | 38,43 | 38,63 |
| *HCG18* | 31,19 | 28,92 | 28,22 | 30,42 | 29,55 |
| *MGC16275* | 38,98 | 40,00 | 35,66 | 37,39 | 37,53 |
| *LOC387723* | 35,71 | 34,76 | 35,65 | 36,07 | 38,16 |
| *EMX20S* | 40,00 | 36,31 | 39,66 | 40,00 | 40,00 |
| *LOC400043* | 37,58 | 40,00 | 34,99 | 29,22 | 39,50 |
| *HYMAI* | 40,00 | 40,00 | 40,00 | 40,00 | 40,00 |
| *B2M* | 23,19 | 25,06 | 21,84 | 23,45 | 24,85 |
| *GUSB* | 27,59 | 27,80 | 26,41 | 26,90 | 25,10 |
| *HPRT1* | 29,59 | 27,04 | 27,58 | 25,97 | 27,38 |
| *PPIA* | 24,99 | 23,48 | 23,05 | 23,06 | 24,56 |
| *RPLP0* | 25,71 | 23,81 | 23,05 | 22,79 | 24,26 |
| *TBP* | 31,26 | 29,62 | 28,57 | 29,38 | 29,35 |
