# Supplementary material for: Circulating long non-coding RNA GAS5 (growth arrest-specific transcript 5) as a complement marker for the detection of malignant mesothelioma using liquid biopsies
Source: Biomark Res. 2020 May 13;8:15. doi: 10.1186/s40364-020-00194-4 (PMC7222324; doi:10.1186/s40364-020-00194-4)
Supplement: Supplementary file 3 — Additional file 3 Detailed characteristics of the individuals used in the performance analyses and corresponding levels of hemoglobin (ng/ml), GAS5 (Ct), RPLP0 (Ct), calretinin (ng/ml), and mesothelin (nM). [file 40364_2020_194_MOESM3_ESM.docx]

**Additional file 3.** Detailed characteristics of the individuals used in the performance analyses and corresponding levels of hemoglobin (g/l), GAS5 (Ct), RPLP0 (Ct), calretinin (ng/ml), and mesothelin (nM).

| **Sample** | **Gender** | **Age** | **Smoking status** | **Group** | **Subtype** | **Hemoglobin (g/l)** | ***GAS5* (Ct)** | ***RPLP0* (Ct)** | **Calretinin (ng/ml)** | **Mesothelin (nM)** |
| --- | --- | --- | --- | --- | --- | --- | --- | --- | --- | --- |
| 1 | Male | 55 | Former | Asbestos-exposed controls |  | 0,09 | 32,31 | 30,45 | 0,120 | 0,737 |
| 2 | Male | 57 | Current | Asbestos-exposed controls |  | 0,09 | 33,08 | 28,22 | 0,155 | n.d. |
| 3 | Male | 55 | Former | Asbestos-exposed controls |  | 0,00 | 28,26 | 26,27 | 0,040 | 0,498 |
| 4 | Male | 59 | Never | Asbestos-exposed controls |  | 0,03 | 29,78 | 28,52 | 0,020 | 0,706 |
| 5 | Male | 53 | Never | Asbestos-exposed controls |  | 0,04 | 28,61 | 28,09 | 0,230 | 0,785 |
| 6 | Male | 68 | Never | Asbestos-exposed controls |  | 0,00 | 29,00 | 28,71 | 0,415 | 2,861 |
| 7 | Male | 71 | Never | Asbestos-exposed controls |  | 0,00 | 31,31 | 30,42 | 0,528 | 1,048 |
| 8 | Male | 71 | Former | Asbestos-exposed controls |  | 0,00 | 30,14 | 30,88 | 0,144 | 1,034 |
| 9 | Male | 75 | Never | Asbestos-exposed controls |  | 0,05 | 33,02 | 30,91 | 0,155 | 1,924 |
| 10 | Male | 76 | Never | Asbestos-exposed controls |  | 0,03 | 28,06 | 27,47 | 0,315 | 1,392 |
| 11 | Male | 67 | Former | Asbestos-exposed controls |  | 0,08 | 26,54 | 27,61 | 0,355 | 1,613 |
| 12 | Male | 67 | n.a. | Asbestos-exposed controls |  | 0,03 | 28,85 | 27,27 | 0,285 | 1,260 |
| 13 | Male | 77 | Never | Asbestos-exposed controls |  | 0,07 | 29,65 | 28,83 | 0,715 | 0,650 |
| 14 | Male | 73 | Former | Asbestos-exposed controls |  | 0,08 | 30,19 | 28,90 | 0,268 | 0,859 |
| 15 | Male | 85 | Former | Asbestos-exposed controls |  | 0,15 | 29,87 | 28,11 | 0,380 | 2,440 |
| 16 | Male | 75 | Never | Asbestos-exposed controls |  | 0,08 | 30,18 | 29,31 | 0,375 | 1,289 |
| 17 | Male | 62 | Former | Asbestos-exposed controls |  | 0,09 | 28,14 | 28,06 | 0,070 | 1,342 |
| 18 | Male | 76 | Former | Asbestos-exposed controls |  | 0,00 | 26,86 | 28,40 | 0,140 | 0,913 |
| 19 | Male | 67 | n.a. | Asbestos-exposed controls |  | 0,08 | 28,02 | 27,89 | 0,010 | 0,938 |
| 20 | Male | 67 | Former | Asbestos-exposed controls |  | 0,07 | 28,34 | 29,23 | 0,150 | 0,595 |
| 21 | Male | 68 | Never | Asbestos-exposed controls |  | 0,08 | 30,96 | 29,01 | 0,300 | 1,093 |
| 22 | Male | 61 | Never | Asbestos-exposed controls |  | 0,07 | 31,93 | 31,57 | 0,020 | 1,100 |
| 23 | Male | 71 | Never | Asbestos-exposed controls |  | 0,09 | 29,29 | 28,85 | 0,290 | 1,075 |
| 24 | Male | 85 | Former | Asbestos-exposed controls |  | n.d. | 29,12 | 28,70 | 0,155 | 1,006 |
| 25 | Male | 61 | Never | Asbestos-exposed controls |  | 0,07 | 30,32 | 30,25 | 0,264 | 0,976 |
| 26 | Male | 76 | Former | Asbestos-exposed controls |  | 0,04 | 34,44 | 32,13 | 0,220 | 1,414 |
| 27 | Male | 49 | Current | Asbestos-exposed controls |  | 0,00 | 31,18 | 28,53 | 0,180 | 1,134 |
| 28 | Male | 72 | Former | Asbestos-exposed controls |  | 0,04 | 30,76 | 29,03 | 0,070 | 0,559 |
| 29 | Male | 72 | Former | Asbestos-exposed controls |  | 0,03 | 27,10 | 30,91 | 1,064 | 0,656 |
| 30 | Male | 75 | Never | Asbestos-exposed controls |  | 0,00 | 29,41 | 31,71 | 0,010 | 2,698 |
| 31 | Male | 72 | Never | Asbestos-exposed controls |  | 0,24 | 28,94 | 33,91 | 0,010 | 0,510 |
| 32 | Male | 85 | Former | Mesothelioma patients | Biphasic | 0,00 | 29,02 | 32,32 | 10,534 | 34,454 |
| 33 | Male | 73 | Former | Mesothelioma patients | n.a. | 0,00 | 25,70 | 29,42 | 2,743 | 12,398 |
| 34 | Male | 68 | Never | Mesothelioma patients | n.a. | 0,02 | 28,36 | 31,43 | 0,162 | 3,497 |
| 35 | Male | 71 | Never | Mesothelioma patients | n.a. | 0,00 | 25,39 | 32,97 | 1,742 | 11,411 |
| 36 | Male | 51 | n.a. | Mesothelioma patients | Epithelioid | 0,08 | 27,54 | 29,14 | 1,514 | 12,088 |
| 37 | Male | 71 | Former | Mesothelioma patients | Epithelioid | 0,00 | 27,72 | 29,47 | 0,370 | 2,015 |
| 38 | Male | 53 | Never | Mesothelioma patients | Epithelioid | 0,02 | 27,59 | 30,07 | 6,646 | 7,114 |
| 39 | Male | 55 | Former | Mesothelioma patients | Epithelioid | 0,01 | 27,47 | 30,02 | 2,293 | 1,701 |
| 40 | Male | 75 | Never | Mesothelioma patients | Sarcomatoid | 0,00 | 25,93 | 30,61 | 0,662 | 0,690 |
| 41 | Male | 71 | Never | Mesothelioma patients | Epithelioid | 0,08 | 27,13 | 29,24 | 1,305 | 1,239 |
| 42 | Male | 67 | n.a. | Mesothelioma patients | Epithelioid | 0,06 | 27,19 | 28,92 | 0,075 | 1,084 |
| 43 | Male | 76 | Never | Mesothelioma patients | Epithelioid | 0,00 | 27,02 | 28,94 | 0,375 | 3,147 |
| 44 | Male | 72 | Never | Mesothelioma patients | Epithelioid | 0,00 | 26,73 | 29,65 | 0,496 | 1,876 |
| 45 | Male | 76 | Former | Mesothelioma patients | n.a. | 0,00 | 26,86 | 28,41 | 0,155 | 0,793 |
| 46 | Male | 76 | Never | Mesothelioma patients | Epithelioid | 0,06 | 26,54 | 28,27 | 1,420 | 1,030 |
| 47 | Male | 75 | Former | Mesothelioma patients | Epithelioid | 0,00 | 29,75 | 29,63 | 0,510 | 1,156 |
| 48 | Male | 77 | Never | Mesothelioma patients | Epithelioid | 0,10 | 24,50 | 29,85 | 0,250 | 1,611 |
| 49 | Male | 67 | Former | Mesothelioma patients | Epithelioid | 0,05 | 26,67 | 29,12 | 2,539 | 42,128 |
| 50 | Male | 72 | Former | Mesothelioma patients | Biphasic | 0,00 | 27,17 | 27,99 | 0,235 | 0,814 |
| 51 | Male | 75 | Never | Mesothelioma patients | Epithelioid | 0,20 | 26,02 | 27,30 | 1,557 | 1,241 |
| 52 | Male | 62 | Former | Mesothelioma patients | Epithelioid | 0,00 | 28,14 | 23,11 | 3,562 | 17,636 |
| 53 | Male | 39 | Never | Mesothelioma patients | Sarcomatoid | 0,14 | 26,24 | 26,95 | 1,044 | 0,642 |
| n.a.: not available | | | | | | | | | | |
| n.d.: not determined | | | | | | | | | | |
