# Supplementary material for: Circulating long non-coding RNA GAS5 (growth arrest-specific transcript 5) as a complement marker for the detection of malignant mesothelioma using liquid biopsies
Source: Biomark Res. 2020 May 13;8:15. doi: 10.1186/s40364-020-00194-4 (PMC7222324; doi:10.1186/s40364-020-00194-4)
Supplement: Supplementary file 4 — Additional file 4. Long non-coding RNAs (lncRNAs) represented on the Affymetrix HG-U133 Plus 2.0 arrays. [file 40364_2020_194_MOESM4_ESM.docx]

**Additional file 4.** Long non-coding RNAs (lncRNAs) represented on the Affymetrix HG-U133 Plus 2.0 arrays.

|  | | | | | | |
| --- | --- | --- | --- | --- | --- | --- |
| **No.** | **Probe set ID** | **Refseq transcript ID** | **Refseq gene symbol** | **Ensembl gene ID** | **Ensembl gene symbol** | **Gene title** |
| gen1 | 207458_at | NR_026785 | *C8orf51* | ENSG00000254389 | *C8orf51* | chromosome 8 open reading frame 51 |
| gen2 | 1563927_a_at | NR_015374 | *LOC401463* | ENSG00000254102 | *RP11-21C4.1* | hypothetical LOC401463 |
| gen3 | 243820_at | NR_015374 | *LOC401463* | ENSG00000254102 | *RP11-21C4.1* | hypothetical LOC401463 |
| gen4 | 1554976_a_at | NR_024378 | *NCRNA00051* | ENSG00000254008 | *NCRNA00051* | non-protein coding RNA 51 |
| gen5 | 1560260_at | NR_027108 /// NR_027109 | *LOC285593* | ENSG00000253955 | *CTB-33O18.3* | hypothetical LOC285593 |
| gen6 | 1562739_at | NR_027108 /// NR_027109 | *LOC285593* | ENSG00000253955 | *CTB-33O18.3* | hypothetical LOC285593 |
| gen7 | 1558828_s_at | NR_027180 | *LOC728264* | ENSG00000253864 | *AC131025.8* | Hypothetical LOC728264 |
| gen8 | 1559965_at | NR_024360 | *LOC100192378* | ENSG00000253661 | *RP11-65D13.1* | hypothetical LOC100192378 |
| gen9 | 1559966_a_at | NR_024360 | *LOC100192378* | ENSG00000253661 | *RP11-65D13.1* | hypothetical LOC100192378 |
| gen10 | 1561257_at | NR_033895 | *LOC286083* | ENSG00000253640 | *CTD-2281E23.1* | hypothetical LOC286083 |
| gen11 | 1569297_at | NR_024441 | *LOC731779* | ENSG00000253595 | *CTD-3064M3.1* | hypothetical LOC731779 |
| gen12 | 1566917_at | NR_026684 | *HPYR1* | ENSG00000253521 | *HPYR1* | Helicobacter pylori responsive 1 (non-protein coding) |
| gen13 | 212337_at | NR_002323 | *TUG1* | ENSG00000253352 | *TUG1* | taurine upregulated 1 (non-protein coding) |
| gen14 | 212725_s_at | NR_002323 | *TUG1* | ENSG00000253352 | *TUG1* | taurine upregulated 1 (non-protein coding) |
| gen15 | 222244_s_at | NR_002323 | *TUG1* | ENSG00000253352 | *TUG1* | taurine upregulated 1 (non-protein coding) |
| gen16 | 1561401_at | NR_027110 | *LOC285627* | ENSG00000253298 | *AC008703.1* | hypothetical LOC285627 |
| gen17 | 1569887_a_at | NR_024473 | *LOC286135* | ENSG00000253279 | *RP11-94H18.3* | hypothetical LOC286135 |
| gen18 | 1556046_a_at | NR_024281 | *LOC157627* | ENSG00000253230 | *RP11-403C10.2* | hypothetical LOC157627 |
| gen19 | 214839_at | NR_024281 | *LOC157627* | ENSG00000253230 | *RP11-403C10.2* | hypothetical LOC157627 |
| gen20 | 1562724_at | NR_033894 | *LOC286114* | ENSG00000253199 | *RP11-421P23.1* | hypothetical LOC286114 |
| gen21 | 244255_at | NR_033894 | *LOC286114* | ENSG00000253199 | *RP11-421P23.1* | hypothetical LOC286114 |
| gen22 | 1556684_at | NR_002312 | *RPPH1* | ENSG00000252678 | *RPPH1* | ribonuclease P RNA component H1 |
| gen23 | 1561514_at | NR_034133 | *LOC400655* | ENSG00000251565 | *AC079062.1* | hypothetical LOC400655 |
| gen24 | 1569264_at | NR_034133 | *LOC400655* | ENSG00000251565 | *AC079062.1* | hypothetical LOC400655 |
| gen25 | 1558678_s_at | NR_002819 | *MALAT1* | ENSG00000251562 | *MALAT1* | metastasis associated lung adenocarcinoma transcript 1 (non-protein coding) |
| gen26 | 224558_s_at | NR_002819 | *MALAT1* | ENSG00000251562 | *MALAT1* | metastasis associated lung adenocarcinoma transcript 1 (non-protein coding) |
| gen27 | 1556505_at | NR_033938 | *LOC100131366* | ENSG00000251533 | *AL161669.2* | hypothetical LOC100131366 |
| gen28 | 1562983_at | NR_033964 | *LOC441025* | ENSG00000251383 | *RP11-542G1.3* | hypothetical LOC441025 |
| gen29 | 231096_at | NR_026555 | *GDEP* | ENSG00000251321 | *RP11-610O8.1* | gene differentially expressed in prostate |
| gen30 | 234017_at | NR_024172 /// NR_024173 | *LOC91948* | ENSG00000251209 | *AC024651.1* | hypothetical LOC91948 |
| gen31 | 232718_at | NR_026765 | *C8orf75* | ENSG00000251191 | *C8orf75* | chromosome 8 open reading frame 75 |
| gen32 | 1557233_at | NR_033900 /// NR_033901 | *LOC285441* | ENSG00000251165 | *RP11-215A19.1* | hypothetical LOC285441 |
| gen33 | 1562617_at | NR_027127 | *LOC340074* | ENSG00000251045 | *CTC-321K16.4* | hypothetical LOC340074 |
| gen34 | 215009_s_at | NR_034075 /// NR_034076 /// NR_034077 | *LOC100499177* | ENSG00000251022 | *RP11-163O17.1* | hypothetical LOC100499177 |
| gen35 | 238241_at | NR_034138 | *LOC100144602* | ENSG00000250846 | *RP11-807H7.1* | hypothetical LOC100144602 |
| gen36 | 1564474_at | NR_024398 | *LOC728723* | ENSG00000250802 | *CTC-564N23.3* | hypothetical LOC728723 |
| gen37 | 1564475_s_at | NR_024398 | *LOC728723* | ENSG00000250802 | *CTC-564N23.3* | hypothetical LOC728723 |
| gen38 | 1570204_at | NR_024398 | *LOC728723* | ENSG00000250802 | *CTC-564N23.3* | hypothetical LOC728723 |
| gen39 | 241423_at | NR_024398 | *LOC728723* | ENSG00000250802 | *CTC-564N23.3* | hypothetical LOC728723 |
| gen40 | 226582_at | NR_026656 | *LOC400043* | ENSG00000250742 | *RP11-834C11.4* | hypothetical LOC400043 |
| gen41 | 1560911_at | NR_034136 | *LOC100133461* | ENSG00000250632 | *RP3-513G18.2* | hypothetical LOC100133461 |
| gen42 | 1563969_at | NR_028351 | *FLJ33360* | ENSG00000250490 | *CTD-2324F15.2* | FLJ33360 protein |
| gen43 | 239569_at | NR_033834 | *FLJ31485* | ENSG00000250208 | *RP11-143E21.7* | hypothetical LOC440119 |
| gen44 | 243351_at | NR_033834 | *FLJ31485* | ENSG00000250208 | *RP11-143E21.7* | hypothetical LOC440119 |
| gen45 | 1557822_at | NR_033976 | *LOC401134* | ENSG00000250125 | *RP11-707A18.1* | hypothetical LOC401134 |
| gen46 | 1557823_s_at | NR_033976 | *LOC401134* | ENSG00000250125 | *RP11-707A18.1* | hypothetical LOC401134 |
| gen47 | 1559033_at | NR_024423 /// NR_024424 | *LOC255167* | ENSG00000250056 | *CTD-2195M18.1* | hypothetical LOC255167 |
| gen48 | 240268_at | NR_033970 | *LOC440117* | ENSG00000249873 | *RP11-983C2.2* | hypothetical LOC440117 |
| gen49 | 240365_at | NR_024391 | *LOC647946* | ENSG00000249862 | *AC016205.1* | hypothetical LOC647946 |
| gen50 | 1558290_a_at | NR_003367 | *PVT1* | ENSG00000249859 | *PVT1* | Pvt1 oncogene (non-protein coding) |
| gen51 | 1562153_a_at | NR_003367 | *PVT1* | ENSG00000249859 | *PVT1* | Pvt1 oncogene (non-protein coding) |
| gen52 | 216240_at | NR_003367 | *PVT1* | ENSG00000249859 | *PVT1* | Pvt1 oncogene (non-protein coding) |
| gen53 | 236950_s_at | NR_027321 | *LOC157381* | ENSG00000249816 | *RP11-6D1.6* | hypothetical LOC157381 |
| gen54 | 1561650_s_at | NR_027112 | *LOC285692* | ENSG00000249781 | *CTD-2143L24.1* | hypothetical LOC285692 |
| gen55 | 1568739_at | NR_027112 | *LOC285692* | ENSG00000249781 | *CTD-2143L24.1* | hypothetical LOC285692 |
| gen56 | 1562251_a_at | NR_033859 | *LOC574538* | ENSG00000249695 | *RP11-598F7.4* | hypothetical LOC574538 |
| gen57 | 214123_s_at | NR_015453 | *C4orf10* | ENSG00000249673 | *C4orf10* | chromosome 4 open reading frame 10 |
| gen58 | 214685_at | NR_015453 | *C4orf10* | ENSG00000249673 | *C4orf10* | chromosome 4 open reading frame 10 |
| gen59 | 215141_at | NR_015453 | *C4orf10* | ENSG00000249673 | *C4orf10* | chromosome 4 open reading frame 10 |
| gen60 | 1558308_at | NR_028415 | *LOC100292680* | ENSG00000249628 | *RP3-340I3.1* | hypothetical LOC100292680 |
| gen61 | 237642_at | NR_033339 | *C4orf42* | ENSG00000249548 | *RP11-20I20.4* | Chromosome 4 open reading frame 42 |
| gen62 | 1562683_a_at | NR_034054 | *LOC285547* | ENSG00000249512 | *AC006445.1* | hypothetical LOC285547 |
| gen63 | 1561096_at | NR_027105 /// NR_027106 | *LOC285419* | ENSG00000249464 | *RP11-93L9.1* | hypothetical LOC285419 |
| gen64 | 208136_s_at | NR_024166 /// NR_024167 | *MGC3771* | ENSG00000249402 | *AC108134.5* | hypothetical LOC81854 |
| gen65 | 239612_at | NR_026657 | *LOC100240734* | ENSG00000249388 | *RP11-834C11.6* | hypothetical LOC100240734 |
| gen66 | 216209_at | NR_034132 | *LOC400084* | ENSG00000249267 | *RP5-916L7.1* | hypothetical LOC400084 |
| gen67 | 238211_at | NR_033920 | *LOC643714* | ENSG00000249231 | *AC026462.2* | hypothetical LOC643714 |
| gen68 | 208346_at | NR_026769 | *PPBPL2* | ENSG00000248848 | *PPBPL2* | pro-platelet basic protein-like 2 |
| gen69 | 1556887_at | NR_002811 | *NCRNA00119* | ENSG00000248724 | *NCRNA00119* | non-protein coding RNA 119 |
| gen70 | 240167_at | NR_033931 | *LOC152742* | ENSG00000248698 | *RP11-341G5.3* | hypothetical LOC152742 |
| gen71 | 230854_at | NR_024049 /// NR_024050 | *BCAR4* | ENSG00000248575 | *BCAR4* | breast cancer anti-estrogen resistance 4 |
| gen72 | 1560723_at | NR_027073 | *LOC283731* | ENSG00000248540 | *AC010931.2* | hypothetical LOC283731 |
| gen73 | 1562901_at | NR_034095 | *LOC400456* | ENSG00000248441 | *AC104260.1* | hypothetical LOC400456 |
| gen74 | 1557296_at | NR_026655 | *FLJ12825* | ENSG00000248265 | *RP11-834C11.3* | hypothetical LOC440101 |
| gen75 | 1556099_at | NR_033918 | *NCRNA00290* | ENSG00000248197 | *NCRNA00290* | non-protein coding RNA 290 |
| gen76 | 1561251_at | NR_033898 | *LOC285577* | ENSG00000248118 | *RP11-121L11.1* | hypothetical LOC285577 |
| gen77 | 1558711_at | NR_002806 | *FAM13AOS* | ENSG00000248019 | *FAM13AOS* | FAM13A opposite strand (non-protein coding) |
| gen78 | 230245_s_at | NR_024433 | *LOC283663* | ENSG00000247982 | *AC016525.1* | hypothetical LOC283663 |
| gen79 | 230648_at | NR_024433 | *LOC283663* | ENSG00000247982 | *AC016525.1* | hypothetical LOC283663 |
| gen80 | 214809_at | NR_037177 /// NR_037178 /// XR_108627 /// XR_110808 /// XR_110973 /// XR_110987 /// XR_111011 /// XR_112858 /// XR_113875 | *LOC100294145* | ENSG00000247909 | *AL669918.1* | hypothetical LOC100294145 |
| gen81 | 236076_at | NR_034107 | *LOC257396* | ENSG00000247796 | *CTD-2366F13.1* | hypothetical LOC257396 |
| gen82 | 241947_at | NR_026544 | *LOC100233209* | ENSG00000247774 | *RP11-493L12.2* | hypothetical LOC100233209 |
| gen83 | 1558256_at | NR_027301 | *LOC148189* | ENSG00000247687 | *AC006504.2* | hypothetical LOC148189 |
| gen84 | 231375_at | NR_026921 | *LOC202181* | ENSG00000247679 | *RP11-1277A3.1* | Hypothetical protein LOC202181 |
| gen85 | 235443_at | NR_034121 /// NR_034122 /// NR_034123 | *LOC100131067* | ENSG00000247572 | *CTC-281B15.1* | hypothetical LOC100131067 |
| gen86 | 242585_at | NR_034121 /// NR_034122 /// NR_034123 | *LOC100131067* | ENSG00000247572 | *CTC-281B15.1* | hypothetical LOC100131067 |
| gen87 | 225332_at | NR_026757 | *LOC729082* | ENSG00000247556 | *AC012652.1* | Hypothetical protein LOC729082 |
| gen88 | 231808_at | NR_026757 | *LOC729082* | ENSG00000247556 | *AC012652.1* | hypothetical LOC729082 |
| gen89 | 1558849_at | NR_015417 | *LOC284276* | ENSG00000247544 | *AC034110.1* | hypothetical LOC284276 |
| gen90 | 1558850_s_at | NR_015417 | *LOC284276* | ENSG00000247544 | *AC034110.1* | hypothetical LOC284276 |
| gen91 | 227925_at | NR_033839 | *FLJ39051* | ENSG00000247445 | *AP001318.3* | hypothetical LOC399972 |
| gen92 | 230999_at | NR_033839 | *FLJ39051* | ENSG00000247445 | *AP001318.3* | Hypothetical protein LOC399972 |
| gen93 | 1559352_a_at | NR_015454 | *LOC92659* | ENSG00000247329 | *AC145207.1* | hypothetical LOC92659 |
| gen94 | 1561850_at | NR_026913 | *LOC100133669* | ENSG00000247317 | *RP11-273G15.2* | similar to hCG1994695 |
| gen95 | 235010_at | NR_034137 | *LOC729013* | ENSG00000247271 | *CTD-2003C8.1* | hypothetical LOC729013 |
| gen96 | 1557826_at | NR_033890 | *LOC338817* | ENSG00000247157 | *RP11-434C1.1* | hypothetical LOC338817 |
| gen97 | 1560202_at | NR_034027 | *LOC338739* | ENSG00000247151 | *RP11-348A11.4* | hypothetical LOC338739 |
| gen98 | 242332_at | NR_033925 /// NR_036444 | *LOC400550* | ENSG00000246979 | *AC009108.1* | hypothetical LOC400550 |
| gen99 | 243059_at | NR_033925 /// NR_036444 | *LOC400550* | ENSG00000246979 | *AC009108.1* | hypothetical LOC400550 |
| gen100 | 1558899_s_at | NR_033932 | *FLJ35946* | ENSG00000246763 | *CTC-463N11.3* | hypothetical protein FLJ35946 |
| gen101 | 1558166_at | NR_026914 | *MGC16275* | ENSG00000246731 | *CTD-2514K5.2* | hypothetical protein MGC16275 |
| gen102 | 1558167_a_at | NR_026914 | *MGC16275* | ENSG00000246731 | *CTD-2514K5.2* | hypothetical protein MGC16275 |
| gen103 | 1561423_at | NR_033858 | *LOC642924* | ENSG00000246662 | *RP11-163D8.1* | hypothetical LOC642924 |
| gen104 | 238244_at | NR_033858 | *LOC642924* | ENSG00000246662 | *RP11-163D8.1* | hypothetical LOC642924 |
| gen105 | 1560707_at | NR_027078 | *LOC283856* | ENSG00000246379 | *AC007495.1* | hypothetical LOC283856 |
| gen106 | 242786_at | NR_036485 | *LOC283104* | ENSG00000246273 | *RP11-540A21.3* | hypothetical LOC283104 |
| gen107 | 1564211_at | NR_015430 | *C14orf64* | ENSG00000246223 | *C14orf64* | chromosome 14 open reading frame 64 |
| gen108 | 239675_at | NR_034148 | *LOC283143* | ENSG00000246100 | *CTC-774J1.2* | hypothetical LOC283143 |
| gen109 | 236112_at | NR_015450 | *LOC285548* | ENSG00000246095 | *AC006445.8* | hypothetical LOC285548 |
| gen110 | 1557465_at | NR_027047 /// NR_027048 | *NCRNA00282* | ENSG00000246013 | *NCRNA00282* | non-protein coding RNA 282 |
| gen111 | 228686_at | NR_015360 | *FLJ33630* | ENSG00000245937 | *CTC-228N24.3* | hypothetical LOC644873 |
| gen112 | 230685_at | NR_015360 | *FLJ33630* | ENSG00000245937 | *CTC-228N24.3* | hypothetical LOC644873 |
| gen113 | 231260_at | NR_037159 /// XR_109499 /// XR_111910 /// XR_115325 | *LOC386758* | ENSG00000245902 | *AC006116.1* | hypothetical LOC386758 |
| gen114 | 238708_at | NR_037159 /// XR_109499 /// XR_111910 /// XR_115325 | *LOC386758* | ENSG00000245902 | *AC006116.1* | hypothetical LOC386758 |
| gen115 | 239594_at | NR_026979 | *LOC145837* | ENSG00000245750 | *AC100826.1* | hypothetical LOC145837 |
| gen116 | 240838_s_at | NR_026979 | *LOC145837* | ENSG00000245750 | *AC100826.1* | hypothetical LOC145837 |
| gen117 | 238021_s_at | NR_034105 /// NR_034106 | *CRNDE* | ENSG00000245694 | *CRNDE* | colorectal neoplasia differentially expressed (non-protein coding) |
| gen118 | 238022_at | NR_034105 /// NR_034106 | *CRNDE* | ENSG00000245694 | *CRNDE* | colorectal neoplasia differentially expressed (non-protein coding) |
| gen119 | 1567361_at | NR_002832 /// NR_033312 /// NR_033313 /// NR_033314 /// NR_033315 | *BDNF-AS* | ENSG00000245573 | *BDNF-AS1* | BDNF antisense RNA (non-protein coding) |
| gen120 | 214657_s_at | NR_028272 | *NEAT1* | ENSG00000245532 | *NEAT1* | nuclear paraspeckle assembly transcript 1 (non-protein coding) |
| gen121 | 224565_at | NR_028272 | *NEAT1* | ENSG00000245532 | *NEAT1* | nuclear paraspeckle assembly transcript 1 (non-protein coding) |
| gen122 | 224566_at | NR_028272 | *NEAT1* | ENSG00000245532 | *NEAT1* | nuclear paraspeckle assembly transcript 1 (non-protein coding) |
| gen123 | 230272_at | NR_015436 /// NR_024383 /// NR_024384 | *LOC645323* | ENSG00000245526 | *CTC-547D20.1* | hypothetical LOC645323 |
| gen124 | 238850_at | NR_015436 /// NR_024383 /// NR_024384 | *LOC645323* | ENSG00000245526 | *CTC-547D20.1* | hypothetical LOC645323 |
| gen125 | 1562606_a_at | NR_033972 | *LOC440028* | ENSG00000245522 | *RP11-540A21.2* | hypothetical LOC440028 |
| gen126 | 1566830_at | NR_033972 | *LOC440028* | ENSG00000245522 | *RP11-540A21.2* | hypothetical LOC440028 |
| gen127 | 1564139_at | NR_026971 | *LOC144571* | ENSG00000245105 | *RP11-436I9.2* | hypothetical LOC144571 |
| gen128 | 1557395_at | NR_034081 | *LOC255130* | ENSG00000245067 | *RP11-12A1.1* | hypothetical LOC255130 |
| gen129 | 1556180_at | NR_027183 | *LOC729678* | ENSG00000245060 | *CTC-205M6.2* | hypothetical LOC729678 |
| gen130 | 227406_at | NR_024490 | *LOC100129387* | ENSG00000244879 | *AC022087.1* | hypothetical LOC100129387 |
| gen131 | 1557369_a_at | NR_027104 | *LOC285401* | ENSG00000244342 | *RP11-129K20.2* | hypothetical LOC285401 |
| gen132 | 1556920_s_at | NR_033843 | *LOC646168* | ENSG00000244227 | *RP11-298O21.5* | hypothetical LOC646168 |
| gen133 | 1560168_at | NR_033843 | *LOC646168* | ENSG00000244227 | *RP11-298O21.5* | hypothetical LOC646168 |
| gen134 | 235606_at | NR_028301 /// NR_028302 | *LOC344595* | ENSG00000243701 | *RP11-446H18.3* | hypothetical LOC344595 |
| gen135 | 239466_at | NR_028301 /// NR_028302 | *LOC344595* | ENSG00000243701 | *RP11-446H18.3* | hypothetical LOC344595 |
| gen136 | 1561402_at | NR_034007 | *LOC339894* | ENSG00000243629 | *RP11-6F2.4* | hypothetical LOC339894 |
| gen137 | 240827_at | NR_024255 /// NR_024256 | *FLJ45983* | ENSG00000243350 | *RP11-379F12.3* | hypothetical LOC399717 |
| gen138 | 1558601_at | NR_015391 | *LOC285194* | ENSG00000243197 | *LSAMP-AS1* | hypothetical LOC285194 |
| gen139 | 1558602_a_at | NR_015391 | *LOC285194* | ENSG00000243197 | *LSAMP-AS1* | hypothetical LOC285194 |
| gen140 | 231898_x_at | NR_004053 | *SOX2OT* | ENSG00000242808 | *SOX2OT* | SOX2 overlapping transcript (non-protein coding) |
| gen141 | 1560762_at | NR_034033 | *LOC285972* | ENSG00000242258 | *RP11-511P7.2* | hypothetical LOC285972 |
| gen142 | 238058_at | NR_027034 | *LOC150381* | ENSG00000241990 | *RP6-109B7.3* | hypothetical LOC150381 |
| gen143 | 1561723_at | NR_034007 | *LOC339894* | ENSG00000241544 | *RP11-6F2.5* | Hypothetical protein LOC339894 |
| gen144 | 1556026_at | NR_027455 | *LOC100131434* | ENSG00000241489 | *AF011889.5* | hypothetical LOC100131434 |
| gen145 | 238283_at | NR_015414 /// NR_024276 | *LOC151658* | ENSG00000241469 | *RP11-631B21.1* | hypothetical LOC151658 |
| gen146 | 1561492_at | NR_033945 | *LOC647107* | ENSG00000241369 | *RP11-10O22.2* | hypothetical LOC647107 |
| gen147 | 240371_at | NR_033945 | *LOC647107* | ENSG00000241369 | *RP11-10O22.2* | hypothetical LOC647107 |
| gen148 | 233604_at | NR_033977 | *FLJ22763* | ENSG00000241224 | *RP11-59E19.1* | hypothetical LOC401081 |
| gen149 | 243687_at | NR_034008 | *LOC100498859* | ENSG00000241135 | *RP11-6F2.6* | hypothetical LOC100498859 |
| gen150 | 230666_at | NR_002795 | *HOXA11-AS* | ENSG00000240990 | *HOXA11-AS1* | HOXA11 antisense RNA (non-protein coding) |
| gen151 | 239950_at | NR_002795 | *HOXA11-AS* | ENSG00000240990 | *HOXA11-AS1* | HOXA11 antisense RNA (non-protein coding) |
| gen152 | 1569882_at | NR_002811 | *NCRNA00119* | ENSG00000240962 /// ENSG00000248724 | *RP11-39E4.1* | non-protein coding RNA 119 |
| gen153 | 1556883_a_at | NR_015361 | *LOC440896* | ENSG00000240907 /// ENSG00000241190 | *BX255923.3* | hypothetical LOC440896 |
| gen154 | 1570388_a_at | NR_015361 | *LOC440896* | ENSG00000240907 /// ENSG00000241190 | *BX255923.3* | hypothetical LOC440896 |
| gen155 | 222090_at | NR_024454 | *LOC100134713* | ENSG00000240889 | *RP4-726N20.2* | hypothetical LOC100134713 |
| gen156 | 1569582_at | NR_026915 | *LOC201651* | ENSG00000240602 | *RP11-64D22.2* | similar to arylacetamide deacetylase (AADAC) |
| gen157 | 1559884_at | NR_003529 | *CDKN2B-AS* | ENSG00000240498 | *CDKN2B-AS1* | CDKN2B antisense RNA (non-protein coding) |
| gen158 | 1556406_at | NR_015400 | *LOC255025* | ENSG00000239589 | *RP11-118N24.3* | hypothetical LOC255025 |
| gen159 | 1560432_at | NR_024066 | *CLRN1OS* | ENSG00000239265 | *CLRN1-AS1* | clarin 1 opposite strand |
| gen160 | 233960_s_at | NR_026927 | *LOC115110* | ENSG00000238164 | *RP3-395M20.8* | hypothetical LOC115110 |
| gen161 | 1558653_at | NR_033882 | *LOC339751* | ENSG00000238133 | *MLK7-AS1* | hypothetical LOC339751 |
| gen162 | 240590_at | NR_033879 | *LOC348761* | ENSG00000238062 | *AC105344.2* | hypothetical LOC348761 |
| gen163 | 232001_at | NR_036502 /// NR_036503 | *LOC439949* | ENSG00000237943 | *RP11-5N23.2* | hypothetical LOC439949 |
| gen164 | 220629_at | NR_024627 | *KCNQ1DN* | ENSG00000237941 | *KCNQ1DN* | KCNQ1 downstream neighbor |
| gen165 | 230452_at | NR_033871 | *FLJ42351* | ENSG00000237753 | *AC079922.3* | hypothetical LOC400999 |
| gen166 | 1557617_at | NR_024463 | *LOC100189589* | ENSG00000237737 | *AC005041.9* | hypothetical LOC100189589 |
| gen167 | 220244_at | NR_024065 | *LOH3CR2A* | ENSG00000237697 | *NCRNA00312* | loss of heterozygosity, 3, chromosomal region 2, gene A |
| gen168 | 1556133_s_at | NR_023362 | *LOC100169752* | ENSG00000237675 | *RP11-118H17.1* | hypothetical LOC100169752 |
| gen169 | 1562844_at | NR_033880 | *LOC339822* | ENSG00000237667 | *AC113607.1* | hypothetical LOC339822 |
| gen170 | 1570006_at | NR_036586 /// XR_041695 /// XR_041696 /// XR_041697 | *LOC400958* | ENSG00000237638 | *AC007386.2* | hypothetical LOC400958 |
| gen171 | 220209_at | NR_003064 | *PYY2* | ENSG00000237575 | *PYY2* | peptide YY, 2 (seminalplasmin) |
| gen172 | 215244_at | NR_002733 /// XR_112221 | *DGCR5 /// LOC100508602* | ENSG00000237517 | *DGCR5* | DiGeorge syndrome critical region gene 5 (non-protein coding) /// hypothetical LOC100508602 |
| gen173 | 228804_at | NR_002733 | *DGCR5* | ENSG00000237517 | *DGCR5* | DiGeorge syndrome critical region gene 5 (non-protein coding) |
| gen174 | 1556801_at | NR_026744 | *LOC400794* | ENSG00000237463 | *RP11-280O1.2* | hypothetical LOC400794 |
| gen175 | 1557761_s_at | NR_026744 | *LOC400794* | ENSG00000237463 | *RP11-280O1.2* | hypothetical LOC400794 |
| gen176 | 1561693_at | NR_026744 | *LOC400794* | ENSG00000237463 | *RP11-280O1.2* | hypothetical LOC400794 |
| gen177 | 243048_at | NR_015352 | *CECR7* | ENSG00000237438 | *CECR7* | cat eye syndrome chromosome region, candidate 7 (non-protein coding) |
| gen178 | 224456_s_at | NR_026878 | *MGC12982* | ENSG00000237424 | *RP11-511I2.5* | hypothetical protein MGC12982 |
| gen179 | 224457_at | NR_026878 | *MGC12982* | ENSG00000237424 | *RP11-511I2.5* | hypothetical protein MGC12982 |
| gen180 | 1570235_at | NR_027310 | *MGC27382* | ENSG00000237413 | *RP11-183M13.1* | hypothetical MGC27382 |
| gen181 | 231447_at | NR_034167 | *MGC44328* | ENSG00000237282 | *RP4-568F9.7* | hypothetical LOC440757 |
| gen182 | 215970_at | NR_027247 | *LOC100130331* | ENSG00000237250 | *RP11-193H5.1* | actin, gamma-like |
| gen183 | 240809_at | NR_027273 | *C21orf121* | ENSG00000237232 | *NCRNA00318* | chromosome 21 open reading frame 121 |
| gen184 | 1556695_a_at | NR_015369 /// NR_021490 /// NR_021491 | *FLJ42709* | ENSG00000237187 | *RP11-65F13.2* | hypothetical LOC441094 |
| gen185 | 1556696_s_at | NR_015369 /// NR_021490 /// NR_021491 | *FLJ42709* | ENSG00000237187 | *RP11-65F13.2* | hypothetical LOC441094 |
| gen186 | 1556771_a_at | NR_024369 | *LOC415056* | ENSG00000237159 | *RP11-296L22.4* | hypothetical LOC415056 |
| gen187 | 219791_s_at | NR_003679 | *NBLA00301* | ENSG00000237125 | *RP11-471J12.1* | Nbla00301 |
| gen188 | 236141_at | NR_003679 | *NBLA00301* | ENSG00000237125 | *RP11-471J12.1* | Nbla00301 |
| gen189 | 1557892_at | NR_033369 | *LOC100144597* | ENSG00000237119 | *RP11-305P22.5* | hypothetical LOC100144597 |
| gen190 | 1557893_a_at | NR_033369 | *LOC100144597* | ENSG00000237119 | *RP11-305P22.5* | hypothetical LOC100144597 |
| gen191 | 224195_at | NR_001551 | *TTTY12* | ENSG00000237048 | *TTTY12* | testis-specific transcript, Y-linked 12 (non-protein coding) |
| gen192 | 228117_at | NR_034118 /// XR_115467 | *LOC100132273 /// LOC100510391* | ENSG00000237037 | *RP4-669P10.18* | hypothetical LOC100132273 /// hypothetical LOC100510391 |
| gen193 | 1553756_at | NR_026663 | *C9orf70* | ENSG00000237009 | *C9orf70* | chromosome 9 open reading frame 70 |
| gen194 | 221422_s_at | NR_026677 | *NCRNA00287* | ENSG00000236901 | *NCRNA00287* | non-protein coding RNA 287 |
| gen195 | 223522_at | NR_026677 | *NCRNA00287* | ENSG00000236901 | *NCRNA00287* | non-protein coding RNA 287 |
| gen196 | 1555196_at | NR_034026 | *LOC100287114* | ENSG00000236834 | *RP11-385E5.4* | hCG2019585-like |
| gen197 | 1569270_at | NR_024453 | *LOC100134368* | ENSG00000236829 | *Z97634.5* | similar to hCG1644121 |
| gen198 | 1562717_at | NR_034135 | *C2orf46* | ENSG00000236790 | *NCRNA00299* | chromosome 2 open reading frame 46 |
| gen199 | 1553440_at | NR_026908 | *C18orf16* | ENSG00000236721 | *CHST9-AS1* | chromosome 18 open reading frame 16 |
| gen200 | 237980_at | NR_034024 /// NR_034025 | *LOC338864* | ENSG00000236678 | *RP11-285E18.1* | hypothetical LOC338864 |
| gen201 | 228972_at | NR_028514 | *LOC100306951* | ENSG00000236618 | *AC100748.2* | hypothetical LOC100306951 |
| gen202 | 239506_s_at | NR_015390 /// NR_024385 | *LOC151300* | ENSG00000236445 | *AC097468.5* | hypothetical LOC151300 |
| gen203 | 239507_at | NR_015390 /// NR_024385 | *LOC151300* | ENSG00000236445 | *AC097468.5* | hypothetical LOC151300 |
| gen204 | 1569604_at | NR_024455 | *LOC100133612* | ENSG00000236423 | *RP13-15E13.1* | similar to hCG1815312 |
| gen205 | 1569895_at | NR_024455 | *LOC100133612* | ENSG00000236423 | *RP13-15E13.1* | similar to hCG1815312 |
| gen206 | 1558212_at | NR_015375 | *FLJ35024* | ENSG00000236404 | *RP11-125B21.2* | hypothetical LOC401491 |
| gen207 | 1553918_at | NR_027272 | *C21orf129* | ENSG00000236384 | *C21orf129* | chromosome 21 open reading frame 129 |
| gen208 | 1560697_at | NR_026836 /// NR_026837 | *LOC283392* | ENSG00000236333 | *AC087886.1* | hypothetical LOC283392 |
| gen209 | 1560698_a_at | NR_026836 /// NR_026837 | *LOC283392* | ENSG00000236333 | *AC087886.1* | hypothetical LOC283392 |
| gen210 | 207288_at | NR_026710 /// NR_026711 | *ASMTL-AS* | ENSG00000236017 | *ASMTL-AS1* | ASMTL antisense RNA (non-protein coding) |
| gen211 | 230566_at | NR_026920 | *C22orf27* | ENSG00000235989 | *MORC2-AS1* | chromosome 22 open reading frame 27 |
| gen212 | 1555871_at | NR_026962 /// NR_026963 | *TTC28-AS* | ENSG00000235954 | *TTC28-AS1* | TTC28 antisense RNA (non-protein coding) |
| gen213 | 232246_at | NR_026962 /// NR_026963 | *TTC28-AS* | ENSG00000235954 | *TTC28-AS1* | TTC28 antisense RNA (non-protein coding) |
| gen214 | 244189_at | NR_026962 /// NR_026963 | *TTC28-AS* | ENSG00000235954 | *TTC28-AS1* | TTC28 antisense RNA (non-protein coding) |
| gen215 | 222314_x_at | NR_004428 | *EGOT* | ENSG00000235947 | *EGOT* | eosinophil granule ontogeny transcript (non-protein coding) |
| gen216 | 240475_at | NR_004428 | *EGOT* | ENSG00000235947 | *EGOT* | eosinophil granule ontogeny transcript (non-protein coding) |
| gen217 | 1554057_at | NR_027023 | *LOC645676* | ENSG00000235919 | *RP11-29H23.1* | hypothetical LOC645676 |
| gen218 | 232230_at | NR_026762 | *NCRNA00263* | ENSG00000235823 | *NCRNA00263* | non-protein coding RNA 263 |
| gen219 | 1556859_a_at | NR_027113 /// NR_027114 | *LOC285740* | ENSG00000235740 | *RP11-436I24.1* | hypothetical LOC285740 |
| gen220 | 229227_at | NR_015415 | *FLJ45244* | ENSG00000235706 | *AL356017.3* | hypothetical locus FLJ45244 |
| gen221 | 1559136_s_at | NR_027456 | *LOC100272228* | ENSG00000235703 /// ENSG00000241769 | *RP13-507I23.1* | hypothetical LOC100272228 |
| gen222 | 1562839_at | NR_027456 | *LOC100272228* | ENSG00000235703 | *RP13-507I23.1* | hypothetical LOC100272228 |
| gen223 | 1569315_s_at | NR_027456 | *LOC100272228* | ENSG00000235703 | *RP13-507I23.1* | hypothetical LOC100272228 |
| gen224 | 1560413_at | NR_015405 | *LOC339788* | ENSG00000235665 | *AC007464.1* | hypothetical LOC339788 |
| gen225 | 243674_at | NR_026658 | *LOC100240735 /// LOC401522* | ENSG00000235659 | *RP11-374M1.5* | hypothetical LOC100240735 /// hypothetical LOC401522 |
| gen226 | 236854_at | NR_026958 | *LOC284749* | ENSG00000235621 | *RP1-66N13.3* | hypothetical LOC284749 |
| gen227 | 230495_at | NR_015399 | *LOC150568* | ENSG00000235597 | *AC013402.2* | hypothetical LOC150568 |
| gen228 | 232881_at | NR_002785 | *GNAS-AS* | ENSG00000235590 | *GNAS-AS1* | GNAS antisense RNA (non-protein coding) |
| gen229 | 230433_at | NR_033998 | *LOC729970* | ENSG00000235501 | *RP4-639F20.1* | hCG2028352-like |
| gen230 | 235362_at | NR_033998 | *LOC729970* | ENSG00000235501 | *RP4-639F20.1* | hCG2028352-like |
| gen231 | 221979_at | NR_033991 /// NR_033992 | *LOC100129250* | ENSG00000235453 | *C9orf133* | hypothetical LOC100129250 |
| gen232 | 78383_at | NR_033991 /// NR_033992 | *LOC100129250* | ENSG00000235453 | *C9orf133* | hypothetical LOC100129250 |
| gen233 | 1557371_a_at | NR_024283 | *LOC158376* | ENSG00000235387 | *RP11-327L3.1* | hypothetical LOC158376 |
| gen234 | 1558123_at | NR_015401 /// NR_024416 | *FLJ35390* | ENSG00000235314 | *AC017116.8* | hypothetical LOC255031 |
| gen235 | 1569089_a_at | NR_015401 /// NR_024416 | *FLJ35390* | ENSG00000235314 | *AC017116.8* | hypothetical LOC255031 |
| gen236 | 1569090_x_at | NR_015401 /// NR_024416 | *FLJ35390* | ENSG00000235314 | *AC017116.8* | hypothetical LOC255031 |
| gen237 | 220354_at | NR_034002 | *LOC100289410* | ENSG00000235280 | *MCF2L-AS1* | hypothetical LOC100289410 |
| gen238 | 230432_at | NR_033557 | *LOC100422737* | ENSG00000235142 | *RP1-60O19.1* | hypothetical LOC100422737 |
| gen239 | 235774_at | NR_033557 | *LOC100422737* | ENSG00000235142 | *RP1-60O19.1* | hypothetical LOC100422737 |
| gen240 | 203245_s_at | NR_015427 | *NCRNA00094* | ENSG00000235106 | *NCRNA00094* | non-protein coding RNA 94 |
| gen241 | 1561383_at | NR_027088 | *LOC284661* | ENSG00000235054 | *RP5-1166F10.1* | hypothetical LOC284661 |
| gen242 | 1561384_a_at | NR_027088 | *LOC284661* | ENSG00000235054 | *RP5-1166F10.1* | hypothetical LOC284661 |
| gen243 | 236351_at | NR_036580 /// XM_001716001 /// XM_001716702 | *LOC389023* | ENSG00000235026 | *AC066593.1* | hypothetical LOC389023 |
| gen244 | 221621_at | NR_027058 | *C17orf86* | ENSG00000234912 | *C17orf86* | chromosome 17 open reading frame 86 |
| gen245 | 229437_at | NR_001458 | *MIR155HG* | ENSG00000234883 | *MIR155HG* | MIR155 host gene (non-protein coding) |
| gen246 | 1562034_at | NR_033840 | *NCRNA00163* | ENSG00000234880 | *NCRNA00163* | non-protein coding RNA 163 |
| gen247 | 1560089_at | NR_033374 | *LOC100289019* | ENSG00000234771 | *RP11-395P17.3* | hypothetical LOC100289019 |
| gen248 | 224741_x_at | NR_002578 | *GAS5* | ENSG00000234741 | *GAS5* | growth arrest-specific 5 (non-protein coding) |
| gen249 | 224841_x_at | NR_002578 | *GAS5* | ENSG00000234741 | *GAS5* | growth arrest-specific 5 (non-protein coding) |
| gen250 | 227517_s_at | NR_002578 | *GAS5* | ENSG00000234741 | *GAS5* | growth arrest-specific 5 (non-protein coding) |
| gen251 | 228238_at | NR_002578 | *GAS5* | ENSG00000234741 | *GAS5* | growth arrest-specific 5 (non-protein coding) |
| gen252 | 1553987_at | NR_015404 | *C12orf47* | ENSG00000234608 | *C12orf47* | chromosome 12 open reading frame 47 |
| gen253 | 212868_x_at | NR_015404 | *C12orf47* | ENSG00000234608 | *C12orf47* | chromosome 12 open reading frame 47 |
| gen254 | 64432_at | NR_015404 | *C12orf47* | ENSG00000234608 | *C12orf47* | chromosome 12 open reading frame 47 |
| gen255 | 1563581_at | NR_026968 | *LOC285456* | ENSG00000234492 | *RP11-462C24.1* | hypothetical LOC285456 |
| gen256 | 1559650_at | NR_034097 | *LOC100128081* | ENSG00000234336 | *AC005017.1* | Hypothetical LOC100128081 |
| gen257 | 230796_at | NR_034128 | *LOC440900* | ENSG00000234199 /// ENSG00000243179 | *AC010982.1* | hypothetical LOC440900 |
| gen258 | 230797_s_at | NR_034128 | *LOC440900* | ENSG00000234199 /// ENSG00000243179 | *AC010982.1* | hypothetical LOC440900 |
| gen259 | 243795_s_at | NR_034128 | *LOC440900* | ENSG00000234199 /// ENSG00000243179 | *AC010982.1* | hypothetical LOC440900 |
| gen260 | 1556378_a_at | NR_015361 | *LOC440896* | ENSG00000234148 /// ENSG00000234394 /// ENSG00000240907 | *AL078621.3* | hypothetical LOC440896 |
| gen261 | 1560823_at | NR_026992 | *LOC340017* | ENSG00000234111 | *RP11-364P22.1* | hypothetical LOC340017 |
| gen262 | 1562681_at | NR_021489 | *LOC338651* | ENSG00000233930 | *RP13-25N22.1* | hypothetical LOC338651 |
| gen263 | 239565_at | NR_024585 | *LOC100128292* | ENSG00000233871 | *RP11-126H7.3* | hypothetical LOC100128292 |
| gen264 | 214983_at | NR_001545 | *TTTY15* | ENSG00000233864 | *TTTY15* | testis-specific transcript, Y-linked 15 (non-protein coding) |
| gen265 | 215756_at | NR_034150 /// NR_034151 | *LOC730227* | ENSG00000233791 | *RP11-134P9.1* | hypothetical LOC730227 |
| gen266 | 232318_s_at | NR_026955 | *NCRNA00284* | ENSG00000233725 | *NCRNA00284* | non-protein coding RNA 284 |
| gen267 | 1557472_a_at | NR_033873 | *FLJ30838* | ENSG00000233723 | *AC007092.1* | hypothetical LOC400955 |
| gen268 | 1562326_at | NR_033873 | *FLJ30838* | ENSG00000233723 | *AC007092.1* | hypothetical LOC400955 |
| gen269 | 207028_at | NR_026766 | *MYCNOS* | ENSG00000233718 | *MYCNOS* | v-myc myelocytomatosis viral related oncogene, neuroblastoma derived (avian) opposite strand |
| gen270 | 216188_at | NR_026766 | *MYCNOS* | ENSG00000233718 | *MYCNOS* | v-myc myelocytomatosis viral related oncogene, neuroblastoma derived (avian) opposite strand |
| gen271 | 1557107_at | NR_028137 | *LOC286002* | ENSG00000233705 | *AC078937.4* | hypothetical LOC286002 |
| gen272 | 1562776_at | NR_034023 | *LOC339807* | ENSG00000233694 | *AC007365.1* | hypothetical LOC339807 |
| gen273 | 223777_at | NR_024004 /// NR_024005 | *DDX11L2* | ENSG00000233614 /// ENSG00000236397 | *Z84812.1* | DEAD/H (Asp-Glu-Ala-Asp/His) box polypeptide 11 like 2 |
| gen274 | 1557891_s_at | NR_034115 | *LOC729178* | ENSG00000233452 | *RP11-497D6.4* | hypothetical LOC729178 |
| gen275 | 238685_at | NR_034115 | *LOC729178* | ENSG00000233452 | *RP11-497D6.4* | hypothetical LOC729178 |
| gen276 | 1564178_at | NR_033846 | *LOC283033* | ENSG00000233395 | *RP11-168P8.7* | hypothetical LOC283033 |
| gen277 | 241525_at | NR_033841 | *LOC200772* | ENSG00000233392 | *AC104809.4* | hypothetical LOC200772 |
| gen278 | 233812_at | NR_024358 | *NCRNA00028* | ENSG00000233354 | *NCRNA00028* | non-protein coding RNA 28 |
| gen279 | 1562805_at | NR_030727 | *LOC349408* | ENSG00000233338 | *GS1-324M7.6* | hypothetical LOC349408 |
| gen280 | 1552467_at | NR_027695 | *DSCR10* | ENSG00000233316 | *DSCR10* | Down syndrome critical region gene 10 |
| gen281 | 1552468_a_at | NR_027695 | *DSCR10* | ENSG00000233316 | *DSCR10* | Down syndrome critical region gene 10 |
| gen282 | 220324_at | NR_026807 | *C6orf155* | ENSG00000233237 | *C6orf155* | chromosome 6 open reading frame 155 |
| gen283 | 1562413_at | NR_024233 | *NCRNA00167* | ENSG00000233220 | *NCRNA00167* | non-protein coding RNA 167 |
| gen284 | 1557656_at | NR_033986 | *LOC400238* | ENSG00000233208 | *AL096869.5* | hypothetical LOC400238 |
| gen285 | 1557657_a_at | NR_033986 | *LOC400238* | ENSG00000233208 | *AL096869.5* | hypothetical LOC400238 |
| gen286 | 1561728_a_at | NR_033986 | *LOC400238* | ENSG00000233208 | *AL096869.5* | hypothetical LOC400238 |
| gen287 | 225635_s_at | NR_036592 | *LOC401504* | ENSG00000233137 | *RP11-220I1.1* | hypothetical LOC401504 |
| gen288 | 225640_at | NR_036592 | *LOC401504* | ENSG00000233137 | *RP11-220I1.1* | hypothetical LOC401504 |
| gen289 | 226635_at | NR_036592 | *LOC401504* | ENSG00000233137 | *RP11-220I1.1* | hypothetical LOC401504 |
| gen290 | 230743_at | NR_033201 /// NR_033202 /// NR_033203 /// NR_033204 /// NR_033205 | *LOC404266* | ENSG00000233101 | *RP11-357H14.7* | hypothetical LOC404266 |
| gen291 | 236892_s_at | NR_033201 /// NR_033202 /// NR_033203 /// NR_033204 /// NR_033205 | *LOC404266* | ENSG00000233101 | *RP11-357H14.7* | hypothetical LOC404266 |
| gen292 | 236893_at | NR_033201 /// NR_033202 /// NR_033203 /// NR_033204 /// NR_033205 | *LOC404266* | ENSG00000233101 | *RP11-357H14.7* | hypothetical LOC404266 |
| gen293 | 239791_at | NR_033201 /// NR_033202 /// NR_033203 /// NR_033204 /// NR_033205 | *LOC404266* | ENSG00000233101 | *RP11-357H14.7* | hypothetical LOC404266 |
| gen294 | 240151_at | NR_033201 /// NR_033202 /// NR_033203 /// NR_033204 /// NR_033205 | *LOC404266* | ENSG00000233101 | *RP11-357H14.7* | hypothetical LOC404266 |
| gen295 | 1566761_a_at | NR_033929 | *FLJ34208* | ENSG00000233058 | *AC046143.7* | hypothetical LOC401106 |
| gen296 | 240767_x_at | NR_026668 | *LOC100286938* | ENSG00000232998 | *RP11-470P4.2* | hypothetical LOC100286938 |
| gen297 | 1553829_at | NR_027252 | *C2orf58* | ENSG00000232973 | *CYP1B1-AS1* | chromosome 2 open reading frame 58 |
| gen298 | 1563904_at | NR_033940 | *LOC100129620* | ENSG00000232825 | *RP5-896L10.1* | hypothetical LOC100129620 |
| gen299 | 1554332_a_at | NR_024470 | *LOC100127888* | ENSG00000232803 | *RP11-93B14.5* | hypothetical LOC100127888 |
| gen300 | 220703_at | NR_024628 /// NR_024629 /// NR_027708 /// NR_027709 | *C10orf110* | ENSG00000232656 | *IDI2-AS1* | chromosome 10 open reading frame 110 |
| gen301 | 1560841_at | NR_033995 | *LOC389247* | ENSG00000232648 | *RP11-367N14.2* | hypothetical LOC389247 |
| gen302 | 220772_at | NR_026783 | *BPESC1* | ENSG00000232416 | *BPESC1* | blepharophimosis, epicanthus inversus and ptosis, candidate 1 (non-protein coding) |
| gen303 | 1560881_a_at | NR_024028 | *NCRNA00112* | ENSG00000232401 | *NCRNA00112* | non-protein coding RNA 112 |
| gen304 | 226236_at | NR_015432 | *LOC388789* | ENSG00000232388 | *RP11-379J5.2* | hypothetical LOC388789 |
| gen305 | 231196_x_at | NR_026795 | *NCRNA00202* | ENSG00000232224 | *NCRNA00202* | non-protein coding RNA 202 |
| gen306 | 222001_x_at | NR_024510 /// NR_024511 | *LOC728855* | ENSG00000232151 /// ENSG00000235398 /// ENSG00000236943 | *RP11-277L2.2* | hypothetical LOC728855 |
| gen307 | 229429_x_at | NR_024510 /// NR_024511 | *LOC728855* | ENSG00000232151 /// ENSG00000235398 /// ENSG00000236943 | *RP11-277L2.2* | hypothetical LOC728855 |
| gen308 | 1559514_at | NR_033937 | *LOC100132077* | ENSG00000232063 | *RP11-307E17.8* | hypothetical LOC100132077 |
| gen309 | 239168_at | NR_026832 | *LOC150622* | ENSG00000232044 | *AC073479.1* | hypothetical LOC150622 |
| gen310 | 243362_s_at | NR_029373 /// NR_029374 | *LOC641518* | ENSG00000232021 | *RP11-558N14.1* | hypothetical LOC641518 |
| gen311 | 243363_at | NR_029373 /// NR_029374 | *LOC641518* | ENSG00000232021 | *RP11-558N14.1* | hypothetical LOC641518 |
| gen312 | 231313_at | NR_033981 | *FLJ27354* | ENSG00000231999 | *RP5-1007M22.2* | hypothetical LOC400761 |
| gen313 | 237236_x_at | NR_026794 | *LOC731789* | ENSG00000231976 | *RP13-16H11.1* | hypothetical LOC731789 |
| gen314 | 243971_x_at | NR_026794 | *LOC731789* | ENSG00000231976 | *RP13-16H11.1* | hypothetical LOC731789 |
| gen315 | 236555_at | NR_034108 /// NR_034109 /// NR_034110 /// NR_034111 | *LOC643749* | ENSG00000231889 | *TRAF3IP2-AS1* | hypothetical LOC643749 |
| gen316 | 235712_at | NR_002578 | *GAS5* | ENSG00000231792 | *RP5-1198E17.1* | Growth arrest-specific 5 (non-protein coding) |
| gen317 | 230882_at | NR_015448 | *DLX6-AS* | ENSG00000231764 | *DLX6-AS1* | DLX6 antisense RNA (non-protein coding) |
| gen318 | 228702_at | NR_015431 /// NR_024153 | *FLJ43663* | ENSG00000231721 | *AC058791.2* | hypothetical LOC378805 |
| gen319 | 238986_at | NR_015431 /// NR_024153 | *FLJ43663* | ENSG00000231721 | *AC058791.2* | hypothetical LOC378805 |
| gen320 | 1556304_s_at | NR_027036 | *LOC100271722* | ENSG00000231711 | *RP11-398F12.1* | hypothetical LOC100271722 |
| gen321 | 220904_at | NR_026780 | *C6orf208* | ENSG00000231690 | *C6orf208* | chromosome 6 open reading frame 208 |
| gen322 | 1564287_at | NR_027039 | *LOC144776* | ENSG00000231674 | *RP11-432D3.1* | hypothetical LOC144776 |
| gen323 | 1568746_a_at | NR_033946 | *LOC646268* | ENSG00000231666 | *RP11-404O13.1* | hCG1654703 |
| gen324 | 215629_s_at | NR_002612 /// NR_002771 | *DLEU2 /// DLEU2L* | ENSG00000231607 | *DLEU2* | deleted in lymphocytic leukemia 2 (non-protein coding) /// deleted in lymphocytic leukemia 2-like |
| gen325 | 1563229_at | NR_002612 | *DLEU2* | ENSG00000231607 | *DLEU2* | deleted in lymphocytic leukemia 2 (non-protein coding) |
| gen326 | 1569600_at | NR_002612 | *DLEU2* | ENSG00000231607 | *DLEU2* | Deleted in lymphocytic leukemia 2 (non-protein coding) |
| gen327 | 216870_x_at | NR_002612 | *DLEU2* | ENSG00000231607 | *DLEU2* | deleted in lymphocytic leukemia 2 (non-protein coding) |
| gen328 | 239936_at | NR_002612 | *DLEU2* | ENSG00000231607 | *DLEU2* | deleted in lymphocytic leukemia 2 (non-protein coding) |
| gen329 | 229059_at | NR_024366 /// NR_024376 | *NCRNA00256A /// NCRNA00256B* | ENSG00000231528 | *NCRNA00256A* | non-protein coding RNA 256A /// non-protein coding RNA 256B |
| gen330 | 1563728_at | NR_026679 /// NR_026687 | *NCRNA00032* | ENSG00000231459 | *NCRNA00032* | non-protein coding RNA 32 |
| gen331 | 1559712_at | NR_024394 | *LOC154822* | ENSG00000231419 | *AC004863.6* | hypothetical LOC154822 |
| gen332 | 1570376_at | NR_024394 | *LOC154822* | ENSG00000231419 | *AC004863.6* | hypothetical LOC154822 |
| gen333 | 230325_at | NR_024444 | *LOC100133985* | ENSG00000231327 | *AC016700.5* | hypothetical LOC100133985 |
| gen334 | 1556900_at | NR_034147 | *LOC149773* | ENSG00000231290 | *RP5-907D15.2* | hypothetical LOC149773 |
| gen335 | 1556901_s_at | NR_034147 | *LOC149773* | ENSG00000231290 | *RP5-907D15.2* | hypothetical LOC149773 |
| gen336 | 223725_at | NR_026829 | *C3orf42* | ENSG00000231177 | *GHRLOS2* | chromosome 3 open reading frame 42 |
| gen337 | 1562558_at | NR_033922 | *LOC440704* | ENSG00000231175 | *RP11-463J7.2* | hypothetical LOC440704 |
| gen338 | 219871_at | NR_026804 | *FLJ13197* | ENSG00000231160 | *RP11-617D20.1* | hypothetical FLJ13197 |
| gen339 | 1561691_at | NR_026969 | *LOC285735* | ENSG00000231023 | *RP11-314E23.1* | hypothetical LOC285735 |
| gen340 | 234847_at | NR_033837 | *LOC150992* | ENSG00000230923 | *NCRNA00309* | hypothetical LOC150992 |
| gen341 | 241972_at | NR_015378 | *LOC401588* | ENSG00000230844 | *RP1-71L16.2* | hypothetical LOC401588 |
| gen342 | 1561978_at | NR_027091 /// NR_027092 /// NR_027093 | *LOC284798* | ENSG00000230725 | *RP4-738P15.1* | hypothetical LOC284798 |
| gen343 | 216786_at | NR_002161 | *NCRNA00230A* | ENSG00000230663 /// ENSG00000233522 | *NCRNA00230B* | non-protein coding RNA 230A |
| gen344 | 235941_s_at | NR_002161 | *NCRNA00230A* | ENSG00000230663 /// ENSG00000233522 | *NCRNA00230B* | non-protein coding RNA 230A |
| gen345 | 1558623_at | NR_024439 | *LOC729121* | ENSG00000230651 | *AC096655.2* | hypothetical LOC729121 |
| gen346 | 1558515_at | NR_028379 | *NCRNA00182* | ENSG00000230590 | *FTX* | non-protein coding RNA 182 |
| gen347 | 242121_at | NR_028379 | *NCRNA00182* | ENSG00000230590 | *FTX* | non-protein coding RNA 182 |
| gen348 | 236403_at | NR_027051 /// NR_027052 | *FLJ39582* | ENSG00000230513 | *AC002472.9* | hypothetical LOC439931 |
| gen349 | 239015_at | NR_027051 /// NR_027052 | *FLJ39582* | ENSG00000230513 | *AC002472.9* | hypothetical LOC439931 |
| gen350 | 1561666_a_at | NR_021487 /// NR_027328 /// NR_027329 | *KIAA1908* | ENSG00000230487 | *AC093734.11* | hypothetical LOC114796 |
| gen351 | 230470_at | NR_026719 | *DSCR9* | ENSG00000230366 | *DSCR9* | Down syndrome critical region gene 9 (non-protein coding) |
| gen352 | 1562829_at | NR_027124 | *LOC339568* | ENSG00000230324 | *RP4-705O1.1* | hypothetical LOC339568 |
| gen353 | 232294_at | NR_027428 /// NR_027429 /// NR_027430 /// NR_027431 /// NR_027432 | *LOC219347* | ENSG00000230091 | *RP11-369J21.5* | hypothetical LOC219347 |
| gen354 | 1562716_at | NR_027087 | *LOC284632* | ENSG00000230023 | *RP11-10N16.2* | hypothetical LOC284632 |
| gen355 | 1557070_at | NR_033910 | *LOC100130275* | ENSG00000229950 | *RP1-290I10.6* | hypothetical LOC100130275 |
| gen356 | 1560503_a_at | NR_033910 | *LOC100130275* | ENSG00000229950 | *RP1-290I10.6* | hypothetical LOC100130275 |
| gen357 | 230963_at | NR_002791 | *EMX2OS* | ENSG00000229847 | *EMX2OS* | EMX2 opposite strand (non-protein coding) |
| gen358 | 214218_s_at | NR_001564 | *XIST* | ENSG00000229807 | *XIST* | X (inactive)-specific transcript (non-protein coding) |
| gen359 | 221728_x_at | NR_001564 | *XIST* | ENSG00000229807 | *XIST* | X (inactive)-specific transcript (non-protein coding) |
| gen360 | 224588_at | NR_001564 | *XIST* | ENSG00000229807 | *XIST* | X (inactive)-specific transcript (non-protein coding) |
| gen361 | 224589_at | NR_001564 | *XIST* | ENSG00000229807 | *XIST* | X (inactive)-specific transcript (non-protein coding) |
| gen362 | 224590_at | NR_001564 | *XIST* | ENSG00000229807 | *XIST* | X (inactive)-specific transcript (non-protein coding) |
| gen363 | 227671_at | NR_001564 | *XIST* | ENSG00000229807 | *XIST* | X (inactive)-specific transcript (non-protein coding) |
| gen364 | 243712_at | NR_001564 | *XIST* | ENSG00000229807 | *XIST* | X (inactive)-specific transcript (non-protein coding) |
| gen365 | 219563_at | NR_026779 | *C14orf139* | ENSG00000229645 | *C14orf139* | chromosome 14 open reading frame 139 |
| gen366 | 230845_at | NR_024103 | *NCRNA00253* | ENSG00000229637 | *HOXB13-AS1* | non-protein coding RNA 253 |
| gen367 | 215590_x_at | NR_028389 | *LOC100128640* | ENSG00000229589 | *RP11-216F19.1* | hypothetical LOC100128640 |
| gen368 | 236158_at | NR_033747 | *LOC728853* | ENSG00000229481 | *AC016582.1* | Similar to RIKEN cDNA 4930432E11 |
| gen369 | 1570447_at | NR_024609 | *FLJ44054* | ENSG00000229373 | *RP11-199F6.8* | hypothetical LOC643365 |
| gen370 | 231421_at | NR_015413 /// NR_024410 /// NR_024411 | *LOC254312* | ENSG00000229240 | *RP1-251M9.1* | hypothetical LOC254312 |
| gen371 | 224293_at | NR_001542 | *TTTY10* | ENSG00000229236 | *TTTY10* | testis-specific transcript, Y-linked 10 (non-protein coding) |
| gen372 | 236986_at | NR_034037 | *LOC100287814* | ENSG00000229228 | *RP11-17H4.2* | hCG2045266-like |
| gen373 | 1561327_at | NR_026781 | *NCRNA00242* | ENSG00000229214 | *NCRNA00242* | non-protein coding RNA 242 |
| gen374 | 1562249_at | NR_033897 | *LOC285965* | ENSG00000229153 | *AC092214.10* | hypothetical LOC285965 |
| gen375 | 238263_at | NR_033897 | *LOC285965* | ENSG00000229153 | *AC092214.10* | hypothetical LOC285965 |
| gen376 | 238387_s_at | NR_033375 | *NCRNA00226* | ENSG00000229084 | *NCRNA00226* | non-protein coding RNA 226 |
| gen377 | 1557037_a_at | NR_036531 /// NR_036532 | *LOC100289373* | ENSG00000228889 | *RP11-87L10.1* | hypothetical LOC100289373 |
| gen378 | 1557038_s_at | NR_036531 /// NR_036532 | *LOC100289373* | ENSG00000228889 | *RP11-87L10.1* | hypothetical LOC100289373 |
| gen379 | 232848_at | NR_033829 | *LOC642345* | ENSG00000228824 | *RP11-342C20.3* | hCG1818123 |
| gen380 | 1557055_s_at | NR_015368 | *LOC643837* | ENSG00000228794 | *RP11-206L10.11* | hypothetical LOC643837 |
| gen381 | 237011_at | NR_015368 | *LOC643837* | ENSG00000228794 | *RP11-206L10.11* | Hypothetical LOC643837 |
| gen382 | 232664_at | NR_033875 | *FLJ12334* | ENSG00000228784 | *AC013400.2* | hypothetical LOC400946 |
| gen383 | 1553634_a_at | NR_015392 | *FLJ40852* | ENSG00000228775 | *RP5-894A10.5* | hypothetical LOC285962 |
| gen384 | 1555363_s_at | NR_026956 | *LOC284440* | ENSG00000228642 | *AC011477.1* | hypothetical LOC284440 |
| gen385 | 1560390_s_at | NR_026956 | *LOC284440* | ENSG00000228642 | *AC011477.1* | hypothetical LOC284440 |
| gen386 | 239153_at | NR_003716 | *HOTAIR* | ENSG00000228630 | *HOTAIR* | hox transcript antisense RNA (non-protein coding) |
| gen387 | 1557996_at | NR_028058 | *LOC100132832* | ENSG00000228434 | *AC004951.6* | postmeiotic segregation increased 2-like 5-like |
| gen388 | 211050_x_at | NR_033908 /// XR_038775 | *LOC100134822 /// LOC100288069* | ENSG00000228327 /// ENSG00000239368 | *RP11-206L10.2* | similar to hCG1739109 /// hypothetical LOC100288069 |
| gen389 | 1558589_at | NR_026755 | *C21orf15* | ENSG00000228314 /// ENSG00000244155 | *CYP4F3LP* | chromosome 21 open reading frame 15 |
| gen390 | 1557167_at | NR_026790 | *HCG11* | ENSG00000228223 | *HCG11* | HLA complex group 11 |
| gen391 | 1557169_x_at | NR_026790 | *HCG11* | ENSG00000228223 | *HCG11* | HLA complex group 11 |
| gen392 | 1556797_at | NR_033997 | *LOC386597* | ENSG00000228203 | *AC017076.4* | hypothetical LOC386597 |
| gen393 | 1556798_a_at | NR_033997 | *LOC386597* | ENSG00000228203 | *AC017076.4* | hypothetical LOC386597 |
| gen394 | 1559145_at | NR_033997 | *LOC386597* | ENSG00000228203 | *AC017076.4* | hypothetical LOC386597 |
| gen395 | 1557591_at | NR_033848 | *LOC283038* | ENSG00000228021 | *RP11-383C5.3* | hypothetical LOC283038 |
| gen396 | 1563528_at | NR_026995 | *LOC91149* | ENSG00000228016 | *AC018712.3* | hypothetical LOC91149 |
| gen397 | 230541_at | NR_015422 | *LOC149134* | ENSG00000227953 | *RP11-439E19.3* | hypothetical LOC149134 |
| gen398 | 237326_at | NR_027266 /// NR_027267 | *C21orf82* | ENSG00000227456 | *NCRNA00310* | chromosome 21 open reading frame 82 |
| gen399 | 234529_at | NR_002769 | *PCGEM1* | ENSG00000227418 | *PCGEM1* | prostate-specific transcript 1 (non-protein coding) |
| gen400 | 1557613_at | NR_033373 | *FLJ39534* | ENSG00000227398 | *RP11-447D11.2* | hypothetical FLJ39534 |
| gen401 | 1569502_s_at | NR_033708 /// NR_033709 /// NR_033710 /// NR_033711 /// NR_033712 | *KIAA0495* | ENSG00000227372 | *TP73-AS1* | KIAA0495 |
| gen402 | 213339_at | NR_033708 /// NR_033709 /// NR_033710 /// NR_033711 /// NR_033712 | *KIAA0495* | ENSG00000227372 | *TP73-AS1* | KIAA0495 |
| gen403 | 213340_s_at | NR_033708 /// NR_033709 /// NR_033710 /// NR_033711 /// NR_033712 | *KIAA0495* | ENSG00000227372 | *TP73-AS1* | KIAA0495 |
| gen404 | 1555912_at | NR_002330 | *ST7OT1* | ENSG00000227199 | *ST7-AS1* | ST7 overlapping transcript 1 (non-protein coding) |
| gen405 | 1562831_a_at | NR_033850 | *LOC283089* | ENSG00000227165 | *RP11-323P17.1* | hypothetical LOC283089 |
| gen406 | 1563916_at | NR_033850 | *LOC283089* | ENSG00000227165 | *RP11-323P17.1* | hypothetical LOC283089 |
| gen407 | 232452_at | NR_027309 | *LOC148824* | ENSG00000227135 | *RP11-978I15.9* | hypothetical LOC148824 |
| gen408 | 244209_at | NR_029380 | *FLJ41350* | ENSG00000227128 | *RP11-107I14.1* | hypothetical LOC399806 |
| gen409 | 227452_at | NR_036488 | *LOC100499467* | ENSG00000227036 | *AC007639.1* | hypothetical LOC100499467 |
| gen410 | 230812_at | NR_036488 | *LOC100499467* | ENSG00000227036 | *AC007639.1* | hypothetical LOC100499467 |
| gen411 | 224870_at | NR_024031 | *KIAA0114* | ENSG00000226950 | *KIAA0114* | KIAA0114 |
| gen412 | 1552466_x_at | NR_026552 /// NR_026553 | *NCRNA00161* | ENSG00000226935 | *NCRNA00161* | non-protein coding RNA 161 |
| gen413 | 1554405_a_at | NR_026552 /// NR_026553 | *NCRNA00161* | ENSG00000226935 | *NCRNA00161* | non-protein coding RNA 161 |
| gen414 | 1557590_at | NR_028339 /// NR_028340 | *LOC100130522* | ENSG00000226924 | *AC139100.1* | hypothetical LOC100130522 |
| gen415 | 234913_at | NR_001525 /// NR_002177 /// NR_002178 | *TTTY4 /// TTTY4B /// TTTY4C* | ENSG00000226906 /// ENSG00000228296 /// ENSG00000235412 | *TTTY4* | testis-specific transcript, Y-linked 4 (non-protein coding) /// testis-specific transcript, Y-linked 4B (non-protein coding) /// testis-specific transcript, Y-linked 4C (non-protein coding) |
| gen416 | 1554301_at | NR_034141 /// NR_034142 | *LOC645591* | ENSG00000226869 | *RP11-203P23.1* | hypothetical LOC645591 |
| gen417 | 1554302_s_at | NR_034141 /// NR_034142 | *LOC645591* | ENSG00000226869 | *RP11-203P23.1* | hypothetical LOC645591 |
| gen418 | 1555892_s_at | NR_024408 | *LOC253039* | ENSG00000226752 | *RP11-27I1.2* | hypothetical LOC253039 |
| gen419 | 231828_at | NR_024408 | *LOC253039* | ENSG00000226752 | *RP11-27I1.2* | hypothetical LOC253039 |
| gen420 | 239958_at | NR_024408 | *LOC253039* | ENSG00000226752 | *RP11-27I1.2* | hypothetical LOC253039 |
| gen421 | 1562475_at | NR_033870 | *DKFZp686O1327* | ENSG00000226674 | *AC074093.1* | hypothetical LOC401014 |
| gen422 | 1556447_at | NR_027102 | *LOC285370* | ENSG00000226567 | *AC018495.2* | hypothetical LOC285370 |
| gen423 | 1556448_a_at | NR_027102 | *LOC285370* | ENSG00000226567 | *AC018495.2* | hypothetical LOC285370 |
| gen424 | 240068_at | NR_024100 | *C21orf130* | ENSG00000226496 | *NCRNA00323* | chromosome 21 open reading frame 130 |
| gen425 | 244322_at | NR_034120 | *LOC646329* | ENSG00000226380 | *AC058791.1* | hypothetical LOC646329 |
| gen426 | 1564790_at | NR_002331 | *ST7OT2* | ENSG00000226367 | *ST7-AS2* | ST7 overlapping transcript 2 (non-protein coding) |
| gen427 | 237808_at | NR_002331 | *ST7OT2* | ENSG00000226367 | *ST7-AS2* | ST7 overlapping transcript 2 (non-protein coding) |
| gen428 | 230177_at | NR_033417 | *GTF2H2B* | ENSG00000226259 | *GTF2H2B* | general transcription factor IIH, polypeptide 2B |
| gen429 | 1556667_at | NR_034096 | *LOC348751* | ENSG00000226124 | *AC073043.2* | hypothetical LOC348751 |
| gen430 | 1564459_at | NR_033845 | *LOC100129427* | ENSG00000226122 | *AC018705.5* | hypothetical LOC100129427 |
| gen431 | 1557098_s_at | NR_003244 | *HAR1A* | ENSG00000225978 | *HAR1A* | highly accelerated region 1A (non-protein coding) |
| gen432 | 1553420_at | NR_026830 | *FLJ32063* | ENSG00000225953 | *AC017096.1* | hypothetical LOC150538 |
| gen433 | 1560010_a_at | NR_026830 | *FLJ32063* | ENSG00000225953 | *AC017096.1* | hypothetical LOC150538 |
| gen434 | 232575_at | NR_015342 | *PCA3* | ENSG00000225937 | *PCA3* | prostate cancer antigen 3 (non-protein coding) |
| gen435 | 232190_x_at | NR_026927 /// XR_108288 /// XR_112079 /// XR_113327 | *LOC100133445 /// LOC115110* | ENSG00000225931 /// ENSG00000238164 | *RP3-395M20.7* | hypothetical LOC100133445 /// hypothetical LOC115110 |
| gen436 | 224020_at | NR_024160 | *MGC4473* | ENSG00000225826 | *RP1-10C16.2* | hypothetical LOC79100 |
| gen437 | 227168_at | NR_003491 /// NR_033319 /// NR_033320 /// NR_033321 | *MIAT* | ENSG00000225783 | *MIAT* | myocardial infarction associated transcript (non-protein coding) |
| gen438 | 228658_at | NR_003491 /// NR_033319 /// NR_033320 /// NR_033321 | *MIAT* | ENSG00000225783 | *MIAT* | myocardial infarction associated transcript (non-protein coding) |
| gen439 | 237322_at | NR_003491 /// NR_033319 /// NR_033320 /// NR_033321 | *MIAT* | ENSG00000225783 | *MIAT* | myocardial infarction associated transcript (non-protein coding) |
| gen440 | 230488_s_at | NR_002783 | *NCRNA00118* | ENSG00000225756 | *NCRNA00118* | non-protein coding RNA 118 |
| gen441 | 233524_at | NR_027341 | *C9orf44* | ENSG00000225511 | *C9orf44* | chromosome 9 open reading frame 44 |
| gen442 | 233525_s_at | NR_027341 | *C9orf44* | ENSG00000225511 | *C9orf44* | chromosome 9 open reading frame 44 |
| gen443 | 1554447_at | NR_024582 | *NCRNA00183* | ENSG00000225470 | *JPX* | non-protein coding RNA 183 |
| gen444 | 214120_at | NR_002727 | *RFPL1S* | ENSG00000225465 | *RFPL1-AS1* | RFPL1 antisense RNA (non-protein coding) |
| gen445 | 1568974_at | NR_027994 | *NHEG1* | ENSG00000225391 | *RP11-55K22.5* | neuroblastoma highly expressed 1 |
| gen446 | 231491_at | NR_024357 | *NCRNA00113* | ENSG00000225298 | *NCRNA00113* | non-protein coding RNA 113 |
| gen447 | 1560472_at | NR_015425 | *LOC338588* | ENSG00000225269 | *RP11-117P22.2* | hypothetical LOC338588 |
| gen448 | 228004_at | NR_001558 | *NCRNA00261* | ENSG00000225074 | *NCRNA00261* | non-protein coding RNA 261 |
| gen449 | 1561390_at | NR_028083 /// NR_028084 | *FAM41AY1 /// FAM41AY2* | ENSG00000224989 /// ENSG00000226362 | *FAM41AY1* | family with sequence similarity 41, member A, Y-linked 1 /// family with sequence similarity 41, member A, Y-linked 2 |
| gen450 | 230595_at | NR_015423 | *LOC572558* | ENSG00000224958 | *RP11-561O23.6* | hypothetical locus LOC572558 |
| gen451 | 1559645_at | NR_033927 | *NCRNA00184* | ENSG00000224939 | *NCRNA00184* | non-protein coding RNA 184 |
| gen452 | 1559646_a_at | NR_033927 | *NCRNA00184* | ENSG00000224939 | *NCRNA00184* | non-protein coding RNA 184 |
| gen453 | 1559254_at | NR_024089 | *NCRNA00162* | ENSG00000224930 | *NCRNA00162* | non-protein coding RNA 162 |
| gen454 | 1557481_a_at | NR_024090 | *C21orf131* | ENSG00000224924 | *NCRNA00320* | chromosome 21 open reading frame 131 |
| gen455 | 1564392_at | NR_024090 | *C21orf131* | ENSG00000224924 | *NCRNA00320* | chromosome 21 open reading frame 131 |
| gen456 | 225920_at | NR_015434 | *LOC148413* | ENSG00000224870 /// ENSG00000239298 | *RP4-758J18.2* | hypothetical LOC148413 |
| gen457 | 1563632_at | NR_033842 | *LOC220980* | ENSG00000224812 | *RP11-18M11.2* | hypothetical LOC220980 |
| gen458 | 1558967_s_at | NR_024052 /// NR_024053 | *HCG18* | ENSG00000224619 /// ENSG00000224705 /// ENSG00000228894 /// ENSG00000230660 /// ENSG00000231074 /// ENSG00000234893 /// ENSG00000235727 | *HCG18* | HLA complex group 18 |
| gen459 | 225577_at | NR_024052 /// NR_024053 | *HCG18* | ENSG00000224619 /// ENSG00000224705 /// ENSG00000228894 /// ENSG00000230660 /// ENSG00000231074 /// ENSG00000234893 /// ENSG00000235727 | *HCG18* | HLA complex group 18 |
| gen460 | 225584_at | NR_024052 /// NR_024053 | *HCG18* | ENSG00000224619 /// ENSG00000224705 /// ENSG00000228894 /// ENSG00000230660 /// ENSG00000231074 /// ENSG00000234893 /// ENSG00000235727 | *HCG18* | HLA complex group 18 |
| gen461 | 242618_at | NR_024052 /// NR_024053 | *HCG18* | ENSG00000224619 /// ENSG00000224705 /// ENSG00000228894 /// ENSG00000230660 /// ENSG00000231074 /// ENSG00000234893 /// ENSG00000235727 | *HCG18* | HLA complex group 18 |
| gen462 | 1563745_a_at | NR_015429 /// NR_024429 /// NR_024431 | *LOC283050* | ENSG00000224596 | *RP11-202P11.1* | hypothetical LOC283050 |
| gen463 | 1563043_at | NR_027103 | *LOC285375* | ENSG00000224514 | *AC093611.1* | hypothetical LOC285375 |
| gen464 | 231571_at | NR_037179 /// NR_037180 /// NR_037181 /// XR_108701 /// XR_110797 /// XR_110953 /// XR_110965 /// XR_110974 /// XR_110988 /// XR_111000 /// XR_111012 | *LOC100507362* | ENSG00000224446 /// ENSG00000224582 /// ENSG00000225426 /// ENSG00000226687 /// ENSG00000229383 /// ENSG00000230674 /// ENSG00000233857 /// ENSG00000235788 | *DAQB-12N14.5* | hypothetical LOC100507362 |
| gen465 | 1557871_at | NR_033844 | *LOC253573* | ENSG00000224406 | *RP11-48F14.1* | hypothetical LOC253573 |
| gen466 | 239242_at | NR_028443 | *LOC100303728* | ENSG00000224281 | *RP3-404F18.2* | hypothetical LOC100303728 |
| gen467 | 228601_at | NR_033979 | *LOC401022* | ENSG00000224189 | *AC009336.23* | hypothetical LOC401022 |
| gen468 | 239182_at | NR_033979 | *LOC401022* | ENSG00000224189 | *AC009336.23* | hypothetical LOC401022 |
| gen469 | 242042_s_at | NR_033979 | *LOC401022* | ENSG00000224189 | *AC009336.23* | hypothetical LOC401022 |
| gen470 | 239325_at | NR_034113 | *LOC729723* | ENSG00000224165 | *AC013267.2* | hypothetical LOC729723 |
| gen471 | 1557850_at | NR_027118 /// NR_027119 | *LOC285954* | ENSG00000224116 | *AC005027.4* | hypothetical LOC285954 |
| gen472 | 1559163_at | NR_027118 /// NR_027119 | *LOC285954* | ENSG00000224116 | *AC005027.4* | hypothetical LOC285954 |
| gen473 | 1561059_a_at | NR_026834 | *LOC152024* | ENSG00000224074 | *AC098971.2* | hypothetical LOC152024 |
| gen474 | 225698_at | NR_015370 | *NCRNA00219* | ENSG00000224032 | *NCRNA00219* | non-protein coding RNA 219 |
| gen475 | 1560714_at | NR_033847 | *FLJ37035* | ENSG00000224023 | *RP11-383C5.4* | hypothetical LOC399821 |
| gen476 | 239483_at | NR_033847 | *FLJ37035* | ENSG00000224023 | *RP11-383C5.4* | hypothetical LOC399821 |
| gen477 | 1563802_at | NR_027085 | *LOC284551* | ENSG00000223907 | *RP11-439L8.4* | hypothetical LOC284551 |
| gen478 | 234611_at | NR_034040 | *LOC100287902* | ENSG00000223776 | *RP11-385F5.2* | hypothetical LOC100287902 |
| gen479 | 226995_at | NR_026943 | *LOC642852* | ENSG00000223768 | *NCRNA00205* | hypothetical LOC642852 |
| gen480 | 228908_s_at | NR_026943 | *LOC642852* | ENSG00000223768 | *NCRNA00205* | hypothetical LOC642852 |
| gen481 | 228909_at | NR_026943 | *LOC642852* | ENSG00000223768 | *NCRNA00205* | hypothetical LOC642852 |
| gen482 | 1555979_at | NR_026874 | *FLJ39609* | ENSG00000223764 | *RP11-54O7.3* | Similar to hCG1995469 |
| gen483 | 1555980_a_at | NR_026874 | *FLJ39609* | ENSG00000223764 | *RP11-54O7.3* | Similar to hCG1995469 |
| gen484 | 1561433_at | NR_033903 | *LOC285103* | ENSG00000223760 | *AC018804.4* | hypothetical LOC285103 |
| gen485 | 235185_s_at | NR_027002 | *LOC388692* | ENSG00000223759 /// ENSG00000235999 | *RP11-353N4.4* | hypothetical LOC388692 |
| gen486 | 235186_at | NR_027002 | *LOC388692* | ENSG00000223759 /// ENSG00000235999 | *RP11-353N4.4* | hypothetical LOC388692 |
| gen487 | 228235_at | NR_024607 | *MGC16121* | ENSG00000223749 | *AC004383.4* | hypothetical protein MGC16121 |
| gen488 | 229784_at | NR_024607 | *MGC16121* | ENSG00000223749 | *AC004383.4* | hypothetical protein MGC16121 |
| gen489 | 227074_at | NR_034089 | *LOC100131564* | ENSG00000223745 | *RP4-717I23.3* | hypothetical LOC100131564 |
| gen490 | 240546_at | NR_036499 | *LOC389043* | ENSG00000223631 | *AC073869.19* | hypothetical LOC389043 |
| gen491 | 229385_s_at | NR_027064 | *PLAC2* | ENSG00000223573 | *PLAC2* | placenta-specific 2 (non-protein coding) |
| gen492 | 244374_at | NR_027064 | *PLAC2* | ENSG00000223573 | *PLAC2* | placenta-specific 2 (non-protein coding) |
| gen493 | 229609_at | NR_024397 | *LOC728190* | ENSG00000223482 /// ENSG00000225484 /// ENSG00000244733 | *RP11-322M19.1* | hypothetical LOC728190 |
| gen494 | 1558794_at | NR_024397 | *LOC728190* | ENSG00000223482 | *RP11-322M19.1* | Hypothetical LOC728190 |
| gen495 | 1563742_at | NR_033957 | *LOC643650* | ENSG00000223477 | *RP11-314P12.2* | hypothetical LOC643650 |
| gen496 | 230251_at | NR_026860 /// NR_026861 | *C6orf176* | ENSG00000223414 | *C6orf176* | chromosome 6 open reading frame 176 |
| gen497 | 240246_at | NR_033907 | *LOC642236* | ENSG00000223346 | *RP11-348I14.4* | Similar to FRG1 protein (FSHD region gene 1 protein) |
| gen498 | 219865_at | NR_023918 /// NR_023919 | *HSPC157* | ENSG00000218510 | *RP1-224A6.4* | hypothetical LOC29092 |
| gen499 | 1553428_at | NR_026970 | *LY86-AS* | ENSG00000216863 | *LY86-AS1* | LY86 antisense RNA (non-protein coding) |
| gen500 | 1556365_at | NR_026970 | *LY86-AS* | ENSG00000216863 | *LY86-AS1* | LY86 antisense RNA (non-protein coding) |
| gen501 | 1556366_s_at | NR_026970 | *LY86-AS* | ENSG00000216863 | *LY86-AS1* | LY86 antisense RNA (non-protein coding) |
| gen502 | 1568609_s_at | NR_024510 /// NR_024511 /// NR_024584 /// NR_027468 /// NR_027469 /// XR_040635 | *FAM91A2 /// FLJ39739 /// LOC100286793 /// LOC728855 /// LOC728875* | ENSG00000215863 /// ENSG00000223804 /// ENSG00000232151 /// ENSG00000235398 /// ENSG00000236943 | *RP11-495P10.2* | family with sequence similarity 91, member A2 /// hypothetical FLJ39739 /// hypothetical LOC100286793 /// hypothetical LOC728855 /// hypothetical LOC728875 |
| gen503 | 1556456_at | NR_027468 | *FLJ39739* | ENSG00000215863 /// ENSG00000223804 | *RP11-495P10.2* | hypothetical FLJ39739 |
| gen504 | 1556457_s_at | NR_027468 | *FLJ39739* | ENSG00000215863 /// ENSG00000223804 | *RP11-495P10.2* | hypothetical FLJ39739 |
| gen505 | 235599_at | NR_015407 | *LOC339535* | ENSG00000215808 | *RP11-371I1.2* | hypothetical LOC339535 |
| gen506 | 224040_at | NR_001541 | *TTTY5* | ENSG00000215560 | *TTTY5* | testis-specific transcript, Y-linked 5 (non-protein coding) |
| gen507 | 1553607_at | NR_027072 | *NCRNA00189* | ENSG00000215533 | *NCRNA00189* | non-protein coding RNA 189 |
| gen508 | 1553608_a_at | NR_027072 | *NCRNA00189* | ENSG00000215533 | *NCRNA00189* | non-protein coding RNA 189 |
| gen509 | 1563088_a_at | NR_026961 | *LOC284837* | ENSG00000215458 | *AP001053.11* | hypothetical LOC284837 |
| gen510 | 1559957_a_at | NR_026943 | *LOC642852* | ENSG00000215447 | *BX322557.10* | hypothetical LOC642852 |
| gen511 | 1569032_at | NR_026943 | *LOC642852* | ENSG00000215447 | *BX322557.10* | hypothetical LOC642852 |
| gen512 | 243656_at | NR_026943 | *LOC642852* | ENSG00000215447 | *BX322557.10* | hypothetical LOC642852 |
| gen513 | 220459_at | NR_002776 | *MCM3AP-AS* | ENSG00000215424 | *MCM3AP-AS1* | MCM3AP antisense RNA (non-protein coding) |
| gen514 | 232740_at | NR_002776 | *MCM3AP-AS* | ENSG00000215424 | *MCM3AP-AS1* | MCM3AP antisense RNA (non-protein coding) |
| gen515 | 232291_at | NR_027349 /// NR_027350 | *MIR17HG* | ENSG00000215417 | *MIR17HG* | MIR17 host gene (non-protein coding) |
| gen516 | 1559412_at | NR_027790 /// NR_027791 | *C21orf34* | ENSG00000215386 | *C21orf34* | chromosome 21 open reading frame 34 |
| gen517 | 1559901_s_at | NR_027790 /// NR_027791 | *C21orf34* | ENSG00000215386 | *C21orf34* | chromosome 21 open reading frame 34 |
| gen518 | 1557876_at | NR_026994 | *LOC340094* | ENSG00000215231 | *CTD-2247C11.3* | hypothetical LOC340094 |
| gen519 | 1557877_s_at | NR_026994 | *LOC340094* | ENSG00000215231 | *CTD-2247C11.3* | hypothetical LOC340094 |
| gen520 | 1553335_x_at | NR_027253 | *LOC285696* | ENSG00000215196 | *AC091878.1* | hypothetical LOC285696 |
| gen521 | 1553130_at | NR_015441 | *LOC652276* | ENSG00000215154 | *AC141586.5* | hypothetical LOC652276 |
| gen522 | 210464_at | NR_026772 | *C8orf71* | ENSG00000215117 | *C8orf71* | chromosome 8 open reading frame 71 |
| gen523 | 240423_at | NR_015364 | *LOC441204* | ENSG00000214870 | *AC004540.5* | hypothetical locus LOC441204 |
| gen524 | 240424_s_at | NR_015364 | *LOC441204* | ENSG00000214870 | *AC004540.5* | hypothetical locus LOC441204 |
| gen525 | 1555040_at | NR_034140 | *C12orf33* | ENSG00000214851 | *C12orf33* | chromosome 12 open reading frame 33 |
| gen526 | 234664_at | NR_028325 /// NR_028326 /// NR_029401 | *LOC100132062 /// LOC100133161 /// LOC731275* | ENSG00000214837 /// ENSG00000230724 | *RP11-261C10.3* | hypothetical LOC100132062 /// hypothetical LOC100133161 /// hypothetical LOC731275 |
| gen527 | 237111_at | NR_033996 | *LOC388942* | ENSG00000214691 | *AC104654.1* | hypothetical LOC388942 |
| gen528 | 210794_s_at | NR_002766 /// NR_003530 /// NR_003531 /// NR_033358 /// NR_033359 /// NR_033360 | *MEG3* | ENSG00000214548 | *MEG3* | maternally expressed 3 (non-protein coding) |
| gen529 | 212732_at | NR_002766 /// NR_003530 /// NR_003531 /// NR_033358 /// NR_033359 /// NR_033360 | *MEG3* | ENSG00000214548 | *MEG3* | maternally expressed 3 (non-protein coding) |
| gen530 | 226210_s_at | NR_002766 /// NR_003530 /// NR_003531 /// NR_033358 /// NR_033359 /// NR_033360 | *MEG3* | ENSG00000214548 | *MEG3* | maternally expressed 3 (non-protein coding) |
| gen531 | 226211_at | NR_002766 /// NR_003530 /// NR_003531 /// NR_033358 /// NR_033359 /// NR_033360 | *MEG3* | ENSG00000214548 | *MEG3* | maternally expressed 3 (non-protein coding) |
| gen532 | 227390_at | NR_002766 /// NR_003530 /// NR_003531 /// NR_033358 /// NR_033359 /// NR_033360 | *MEG3* | ENSG00000214548 | *MEG3* | maternally expressed 3 (non-protein coding) |
| gen533 | 210795_s_at | NR_002766 /// NR_003530 /// NR_003531 /// NR_033358 /// NR_033359 /// NR_033360 | *MEG3* | ENSG00000214512 | *AL117190.1* | Maternally expressed 3 (non-protein coding) |
| gen534 | 1562048_at | NR_026934 | *LOC152225* | ENSG00000214407 /// ENSG00000241280 | *RP11-221J22.1* | hypothetical LOC152225 |
| gen535 | 1569332_at | NR_026767 | *C3orf66* | ENSG00000214381 | *C3orf66* | chromosome 3 open reading frame 66 |
| gen536 | 1564539_at | NR_033944 | *LOC647323* | ENSG00000214146 | *RP11-699L21.1* | hypothetical LOC647323 |
| gen537 | 1564485_at | NR_024480 | *LOC100131551* | ENSG00000214145 | *RP11-513G11.1* | hypothetical LOC100131551 |
| gen538 | 224278_at | NR_023391 | *C2orf14* | ENSG00000214081 /// ENSG00000239402 /// ENSG00000241259 /// ENSG00000241606 | *C2orf14* | chromosome 2 open reading frame 14 |
| gen539 | 227919_at | NR_015379 | *UCA1* | ENSG00000214049 | *UCA1* | urothelial cancer associated 1 (non-protein coding) |
| gen540 | 1561294_a_at | NR_015398 | *LOC100128554* | ENSG00000214043 | *RP5-944M2.3* | hypothetical LOC100128554 |
| gen541 | 231540_at | NR_026966 | *LOC100130691* | ENSG00000213963 | *AC074286.1* | hypothetical LOC100130691 |
| gen542 | 218624_s_at | NR_026052 | *MGC2752* | ENSG00000213753 | *AC016629.2* | hypothetical LOC65996 |
| gen543 | 222694_at | NR_026052 | *MGC2752* | ENSG00000213753 | *AC016629.2* | hypothetical LOC65996 |
| gen544 | 230528_s_at | NR_026052 | *MGC2752* | ENSG00000213753 | *AC016629.2* | hypothetical LOC65996 |
| gen545 | 1558791_at | NR_026975 | *LOC286467* | ENSG00000213468 | *RP11-453F18__B.1* | hypothetical LOC286467 |
| gen546 | 1556737_at | NR_027254 | *LOC388387* | ENSG00000213373 | *AC100793.2* | hypothetical LOC388387 |
| gen547 | 227941_at | NR_036496 | *LOC339803* | ENSG00000212978 | *AC016747.3* | hypothetical LOC339803 |
| gen548 | 216665_s_at | NR_001536 | *TTTY2* | ENSG00000212855 /// ENSG00000212856 /// ENSG00000235059 /// ENSG00000236951 | *TTTY2* | testis-specific transcript, Y-linked 2 (non-protein coding) |
| gen549 | 217261_at | NR_001536 /// NR_003590 | *TTTY2 /// TTTY2B* | ENSG00000212855 /// ENSG00000212856 | *TTTY2* | testis-specific transcript, Y-linked 2 (non-protein coding) /// testis-specific transcript, Y-linked 2B (non-protein coding) |
| gen550 | 240912_x_at | NR_026949 | *NCRNA00277* | ENSG00000212766 | *NCRNA00277* | non-protein coding RNA 277 |
| gen551 | 1555858_at | NR_027007 | *LOC440944* | ENSG00000206573 | *RP11-58B17.1* | hypothetical LOC440944 |
| gen552 | 1555860_x_at | NR_027007 | *LOC440944* | ENSG00000206573 | *RP11-58B17.1* | hypothetical LOC440944 |
| gen553 | 1557357_at | NR_027007 | *LOC440944* | ENSG00000206573 | *RP11-58B17.1* | hypothetical LOC440944 |
| gen554 | 232052_at | NR_027007 | *LOC440944* | ENSG00000206573 | *RP11-58B17.1* | hypothetical LOC440944 |
| gen555 | 1562381_at | NR_026972 /// NR_026973 | *LOC285830* | ENSG00000206508 /// ENSG00000226378 /// ENSG00000230017 | *HLA-F-AS1* | hypothetical LOC285830 |
| gen556 | 222279_at | NR_026972 /// NR_026973 | *LOC285830* | ENSG00000206446 /// ENSG00000206508 /// ENSG00000214922 /// ENSG00000226378 /// ENSG00000230017 /// ENSG00000239257 /// ENSG00000239784 /// ENSG00000240845 /// ENSG00000241880 /// ENSG00000242039 | *HLA-F-AS1* | hypothetical LOC285830 |
| gen557 | 1559050_at | NR_026791 | *HCG27* | ENSG00000206344 /// ENSG00000220890 /// ENSG00000223577 /// ENSG00000231281 /// ENSG00000232758 /// ENSG00000234079 | *HCG27* | HLA complex group 27 |
| gen558 | 223628_at | NR_026815 | *TMEM191A* | ENSG00000206140 /// ENSG00000226287 | *TMEM191C* | transmembrane protein 191A |
| gen559 | 226924_at | NR_024484 | *LOC400657* | ENSG00000206029 | *AC116904.1* | hypothetical LOC400657 |
| gen560 | 1560320_a_at | NR_026947 | *LOC283314* | ENSG00000205885 | *ABC12-49244600F4.3* | hypothetical LOC283314 |
| gen561 | 1561127_at | NR_033387 | *NCRNA00168* | ENSG00000205696 | *NCRNA00168* | non-protein coding RNA 168 |
| gen562 | 1558046_x_at | NR_034031 | *LOC389906* | ENSG00000205663 /// ENSG00000205664 /// ENSG00000234449 | *RP11-706O15.5* | hypothetical LOC389906 |
| gen563 | 1569629_x_at | NR_034031 | *LOC389906* | ENSG00000205663 /// ENSG00000205664 /// ENSG00000234449 | *RP11-706O15.5* | hypothetical LOC389906 |
| gen564 | 222031_at | NR_034031 | *LOC389906* | ENSG00000205663 /// ENSG00000205664 /// ENSG00000234449 | *RP11-706O15.5* | hypothetical LOC389906 |
| gen565 | 222196_at | NR_034031 | *LOC389906* | ENSG00000205662 /// ENSG00000205663 /// ENSG00000234449 | *RP11-706O15.7* | hypothetical LOC389906 |
| gen566 | 59433_at | NR_034031 | *LOC389906* | ENSG00000205662 /// ENSG00000205663 /// ENSG00000205664 /// ENSG00000234449 | *RP11-706O15.7* | hypothetical LOC389906 |
| gen567 | 1555988_a_at | NR_026828 | *LOC126536* | ENSG00000205396 | *AC004790.2* | hypothetical LOC126536 |
| gen568 | 237242_at | NR_026828 | *LOC126536* | ENSG00000205396 | *AC004790.2* | hypothetical LOC126536 |
| gen569 | 236001_at | NR_036581 | *LOC400573* | ENSG00000205351 | *AC002347.2* | hypothetical LOC400573 |
| gen570 | 243402_at | NR_015406 /// NR_029405 | *LOC149837 /// LOC643406* | ENSG00000205181 /// ENSG00000230563 | *RP5-1022P6.6* | hypothetical LOC149837 /// hypothetical LOC643406 |
| gen571 | 229992_at | NR_015406 | *LOC149837* | ENSG00000205181 | *RP5-1022P6.6* | hypothetical LOC149837 |
| gen572 | 1562601_at | NR_033831 | *UNQ6975* | ENSG00000205054 | *AC009236.1* | putative uncharacterized protein UNQ6975/PRO21958 |
| gen573 | 229094_at | NR_027040 | *LOC401431* | ENSG00000204934 | *RP4-751H13.6* | hypothetical LOC401431 |
| gen574 | 242350_s_at | NR_034129 | *LOC100128098* | ENSG00000204832 | *RP11-414K1.3* | hypothetical LOC100128098 |
| gen575 | 233491_at | NR_026682 /// NR_026683 | *LOC100268168* | ENSG00000204758 | *CTC-308K20.1* | hypothetical LOC100268168 |
| gen576 | 1557483_at | NR_027089 /// NR_027090 | *LOC284788* | ENSG00000204684 | *RP5-1004I9.1* | hypothetical LOC284788 |
| gen577 | 237298_at | NR_027257 | *FLJ26850* | ENSG00000204666 | *CTD-2126E3.1* | FLJ26850 protein |
| gen578 | 1568987_at | NR_026680 /// NR_027295 | *MGC57346* | ENSG00000204650 | *AC126544.1* | hypothetical LOC401884 |
| gen579 | 208287_at | NR_028032 | *HCG9* | ENSG00000204625 /// ENSG00000206504 /// ENSG00000227647 /// ENSG00000234313 /// ENSG00000236579 /// ENSG00000237812 | *HCG9* | HLA complex group 9 |
| gen580 | 234493_at | NR_026670 | *LOC116437* | ENSG00000204603 | *RP11-638F5.1* | hypothetical LOC116437 |
| gen581 | 238997_at | NR_026816 | *PSORS1C3* | ENSG00000204528 /// ENSG00000223364 /// ENSG00000224056 /// ENSG00000224744 /// ENSG00000226422 /// ENSG00000230983 /// ENSG00000231450 | *PSORS1C3* | psoriasis susceptibility 1 candidate 3 (non-protein coding) |
| gen582 | 225028_at | NR_015367 | *LOC550643* | ENSG00000204272 | *RP11-622K12.1* | hypothetical LOC550643 |
| gen583 | 225029_at | NR_015367 | *LOC550643* | ENSG00000204272 | *RP11-622K12.1* | hypothetical LOC550643 |
| gen584 | 238872_at | NR_027276 | *LOC100128239* | ENSG00000204241 | *RP11-713P17.3* | hypothetical LOC100128239 |
| gen585 | 239151_at | NR_029396 | *LOC399753* | ENSG00000204164 /// ENSG00000204177 | *RP11-508M1.3* | hypothetical LOC399753 |
| gen586 | 220660_at | NR_024032 | *C9orf27* | ENSG00000204148 | *C9orf27* | chromosome 9 open reading frame 27 |
| gen587 | 230236_at | NR_024015 | *TDRG1* | ENSG00000204091 | *RP11-535K1.1* | testis development related protein 1 |
| gen588 | 1556896_at | NR_034124 | *LOC284751* | ENSG00000203999 | *RP11-290F20.1* | hypothetical LOC284751 |
| gen589 | 231586_at | NR_034168 | *LOC642864* | ENSG00000203897 | *RP11-475E11.5* | spermatogenesis-related protein 7 |
| gen590 | 235126_at | NR_027285 /// NR_027286 | *NCRNA00292* | ENSG00000198468 | *NCRNA00292* | non-protein coding RNA 292 |
| gen591 | 210409_at | NR_027906 | *C6orf124* | ENSG00000198221 | *C6orf124* | chromosome 6 open reading frame 124 |
| gen592 | 1553484_at | NR_029451 | *C12orf67* | ENSG00000197503 | *C12orf67* | chromosome 12 open reading frame 67 |
| gen593 | 216276_s_at | NR_001569 /// NR_024106 /// NR_024107 | *ADAM3A* | ENSG00000197475 /// ENSG00000253922 | *ADAM3A* | ADAM metallopeptidase domain 3A |
| gen594 | 240192_at | NR_024255 /// NR_024256 | *FLJ45983* | ENSG00000197308 | *RP11-379F12.7* | hypothetical LOC399717 |
| gen595 | 1561490_at | NR_015356 /// NR_033664 /// NR_033665 | *AAA1* | ENSG00000197085 | *AC005688.1* | asthma-associated alternatively spliced gene 1 |
| gen596 | 234117_at | NR_015356 /// NR_033664 /// NR_033665 | *AAA1* | ENSG00000197085 | *AC005688.1* | asthma-associated alternatively spliced gene 1 |
| gen597 | 1556736_at | NR_033939 | *LOC100129858* | ENSG00000196951 | *RP11-425I13.3* | hypothetical LOC100129858 |
| gen598 | 235869_at | NR_033939 | *LOC100129858* | ENSG00000196951 | *RP11-425I13.3* | hypothetical LOC100129858 |
| gen599 | 225857_s_at | NR_015366 /// NR_027241 | *LOC388796* | ENSG00000196756 | *RP4-564F22.2* | hypothetical LOC388796 |
| gen600 | 65588_at | NR_015366 /// NR_027241 | *LOC388796* | ENSG00000196756 | *RP4-564F22.2* | hypothetical LOC388796 |
| gen601 | 1563369_at | NR_027345 /// NR_027346 | *NCRNA00173* | ENSG00000196668 | *NCRNA00173* | non-protein coding RNA 173 |
| gen602 | 237591_at | NR_027345 /// NR_027346 | *NCRNA00173* | ENSG00000196668 | *NCRNA00173* | non-protein coding RNA 173 |
| gen603 | 237205_at | NR_024338 /// NR_024339 | *NCRNA00238* | ENSG00000196553 | *NCRNA00238* | non-protein coding RNA 238 |
| gen604 | 233355_at | NR_027686 /// NR_027687 | *NCRNA00176* | ENSG00000196421 | *NCRNA00176* | non-protein coding RNA 176 |
| gen605 | 215985_at | NR_026751 | *NCRNA00171* | ENSG00000196299 /// ENSG00000204623 /// ENSG00000225618 /// ENSG00000229653 /// ENSG00000232691 /// ENSG00000235394 /// ENSG00000235814 /// ENSG00000236598 | *BX088647.1* | Non-protein coding RNA 171 |
| gen606 | 234097_s_at | NR_026751 | *ZNRD1-AS* | ENSG00000196299 /// ENSG00000204623 /// ENSG00000225618 /// ENSG00000229653 /// ENSG00000232691 /// ENSG00000235394 /// ENSG00000235814 /// ENSG00000236598 | *BX088647.1* | ZNRD1 antisense RNA (non-protein coding) |
| gen607 | 234457_at | NR_026751 | *ZNRD1-AS* | ENSG00000196299 /// ENSG00000204623 /// ENSG00000225618 /// ENSG00000229653 /// ENSG00000232691 /// ENSG00000235394 /// ENSG00000235814 /// ENSG00000236598 | *BX088647.1* | ZNRD1 antisense RNA (non-protein coding) |
| gen608 | 227474_at | NR_015377 | *LOC654433* | ENSG00000189223 | *AC016683.6* | hypothetical LOC654433 |
| gen609 | 228425_at | NR_015377 | *LOC654433* | ENSG00000189223 | *AC016683.6* | hypothetical LOC654433 |
| gen610 | 231229_at | NR_024192 /// NR_024193 | *HILS1* | ENSG00000188662 | *HILS1* | histone linker H1 domain, spermatid-specific 1 |
| gen611 | 236631_at | NR_026960 | *C21orf125* | ENSG00000188660 | *NCRNA00319* | chromosome 21 open reading frame 125 |
| gen612 | 225786_at | NR_026778 | *NCRNA00201* | ENSG00000188206 | *NCRNA00201* | non-protein coding RNA 201 |
| gen613 | 1558423_at | NR_026999 | *NCRNA00265* | ENSG00000188185 | *NCRNA00265* | non-protein coding RNA 265 |
| gen614 | 1558425_x_at | NR_026999 | *NCRNA00265* | ENSG00000188185 | *NCRNA00265* | non-protein coding RNA 265 |
| gen615 | 219840_s_at | NR_028288 | *TCL6* | ENSG00000187621 | *TCL6* | T-cell leukemia/lymphoma 6 (non-protein coding) |
| gen616 | 221054_s_at | NR_028288 | *TCL6* | ENSG00000187621 | *TCL6* | T-cell leukemia/lymphoma 6 (non-protein coding) |
| gen617 | 221624_at | NR_028288 | *TCL6* | ENSG00000187621 | *TCL6* | T-cell leukemia/lymphoma 6 (non-protein coding) |
| gen618 | 239964_at | NR_028288 | *TCL6* | ENSG00000187621 | *TCL6* | T-cell leukemia/lymphoma 6 (non-protein coding) |
| gen619 | 214696_at | NR_028502 /// NR_028503 /// NR_028504 /// NR_028505 | *C17orf91* | ENSG00000186594 | *C17orf91* | chromosome 17 open reading frame 91 |
| gen620 | 1557557_at | NR_034182 | *LOC100129196* | ENSG00000186056 | *RP5-1166H10.2* | hypothetical LOC100129196 |
| gen621 | 1557558_s_at | NR_034182 | *LOC100129196* | ENSG00000186056 | *RP5-1166H10.2* | hypothetical LOC100129196 |
| gen622 | 233562_at | NR_026827 | *LOC84856* | ENSG00000185904 | *RP11-178A10.1* | hypothetical LOC84856 |
| gen623 | 230195_at | NR_036513 | *LOC100131138* | ENSG00000185847 | *RP1-46F2.2* | hypothetical LOC100131138 |
| gen624 | 1559920_a_at | NR_024482 /// NR_024483 | *CECR4* | ENSG00000185837 | *CECR5-AS1* | cat eye syndrome chromosome region, candidate 4 (non-protein coding) |
| gen625 | 231303_at | NR_024027 | *NCRNA00158* | ENSG00000185433 | *NCRNA00158* | non-protein coding RNA 158 |
| gen626 | 239968_at | NR_026863 | *C21orf84* | ENSG00000185186 | *NCRNA00313* | chromosome 21 open reading frame 84 |
| gen627 | 240589_at | NR_026863 | *C21orf84* | ENSG00000185186 | *NCRNA00313* | chromosome 21 open reading frame 84 |
| gen628 | 224292_at | NR_001537 | *TTTY13* | ENSG00000184991 | *TTTY13* | testis-specific transcript, Y-linked 13 (non-protein coding) |
| gen629 | 244829_at | NR_027793 | *C6orf218* | ENSG00000183674 | *C6orf218* | chromosome 6 open reading frame 218 |
| gen630 | 1555554_at | NR_026760 | *BASE* | ENSG00000183566 | *RP11-49G10.8* | breast cancer and salivary gland expression gene |
| gen631 | 1564017_at | NR_027498 /// NR_028082 | *NCRNA00175* | ENSG00000183535 | *NCRNA00175* | non-protein coding RNA 175 |
| gen632 | 224142_s_at | NR_001533 /// NR_003591 | *TTTY8 /// TTTY8B* | ENSG00000183385 /// ENSG00000185700 | *TTTY8* | testis-specific transcript, Y-linked 8 (non-protein coding) /// testis-specific transcript, Y-linked 8B (non-protein coding) |
| gen633 | 224143_at | NR_001533 /// NR_003591 | *TTTY8 /// TTTY8B* | ENSG00000183385 /// ENSG00000185700 | *TTTY8* | testis-specific transcript, Y-linked 8 (non-protein coding) /// testis-specific transcript, Y-linked 8B (non-protein coding) |
| gen634 | 209917_s_at | NR_015381 | *TP53TG1* | ENSG00000182165 | *TP53TG1* | TP53 target 1 (non-protein coding) |
| gen635 | 210241_s_at | NR_015381 | *TP53TG1* | ENSG00000182165 | *TP53TG1* | TP53 target 1 (non-protein coding) |
| gen636 | 210886_x_at | NR_015381 | *TP53TG1* | ENSG00000182165 | *TP53TG1* | TP53 target 1 (non-protein coding) |
| gen637 | 1561537_at | NR_036498 | *LOC388906* | ENSG00000182057 | *Z83851.3* | hypothetical LOC388906 |
| gen638 | 1556704_s_at | NR_024443 /// XR_110887 | *LOC100133920 /// LOC286297* | ENSG00000182021 | *RP11-381O7.3* | hypothetical LOC100133920 /// hypothetical LOC286297 |
| gen639 | 1564277_a_at | NR_024443 /// XR_110887 | *LOC100133920 /// LOC286297* | ENSG00000182021 | *RP11-381O7.3* | hypothetical LOC100133920 /// hypothetical LOC286297 |
| gen640 | 1554764_a_at | NR_026946 | *C11orf64* | ENSG00000181995 | *NCRNA00301* | chromosome 11 open reading frame 64 |
| gen641 | 1554765_a_at | NR_026946 | *C11orf64* | ENSG00000181995 | *NCRNA00301* | chromosome 11 open reading frame 64 |
| gen642 | 1563145_at | NR_026946 | *C11orf64* | ENSG00000181995 | *NCRNA00301* | chromosome 11 open reading frame 64 |
| gen643 | 1552979_at | NR_024079 | *C2orf52* | ENSG00000181798 | *C2orf52* | chromosome 2 open reading frame 52 |
| gen644 | 241475_at | NR_015445 | *BREA2* | ENSG00000181097 | *RP11-429J17.2* | breast cancer estrogen-induced apoptosis 2 |
| gen645 | 224174_at | NR_001548 | *TTTY11* | ENSG00000180910 | *TTTY11* | testis-specific transcript, Y-linked 11 (non-protein coding) |
| gen646 | 1562953_s_at | NR_015359 | *NCRNA00247* | ENSG00000180769 | *NCRNA00247* | non-protein coding RNA 247 |
| gen647 | 238081_at | NR_015359 | *NCRNA00247* | ENSG00000180769 | *NCRNA00247* | non-protein coding RNA 247 |
| gen648 | 241401_at | NR_015359 | *NCRNA00247* | ENSG00000180769 | *NCRNA00247* | non-protein coding RNA 247 |
| gen649 | 1553449_at | NR_024347 | *C16orf81* | ENSG00000180422 | *NCRNA00304* | chromosome 16 open reading frame 81 |
| gen650 | 1553450_s_at | NR_024347 | *C16orf81* | ENSG00000180422 | *NCRNA00304* | chromosome 16 open reading frame 81 |
| gen651 | 241895_at | NR_026758 | *LOC440905* | ENSG00000180178 /// ENSG00000239402 /// ENSG00000241017 | *AC018865.8* | hypothetical LOC440905 |
| gen652 | 220747_at | NR_026883 /// NR_026884 | *HSPC072* | ENSG00000179935 | *RP5-1068E13.3* | hypothetical LOC29075 |
| gen653 | 238096_at | NR_024349 | *LOC284023* | ENSG00000179859 | *AC025335.1* | hypothetical LOC284023 |
| gen654 | 1557727_at | NR_033872 | *LOC400960* | ENSG00000179818 | *AC136007.2* | hypothetical LOC400960 |
| gen655 | 1568640_at | NR_033872 | *LOC400960* | ENSG00000179818 | *AC136007.2* | Hypothetical gene supported by BC040598 |
| gen656 | 227969_at | NR_033872 | *LOC400960* | ENSG00000179818 | *AC136007.2* | hypothetical LOC400960 |
| gen657 | 235137_at | NR_033872 | *LOC400960* | ENSG00000179818 | *AC136007.2* | hypothetical LOC400960 |
| gen658 | 235482_at | NR_033872 | *LOC400960* | ENSG00000179818 | *AC136007.2* | hypothetical LOC400960 |
| gen659 | 227664_at | NR_024279 | *FLJ37453* | ENSG00000179743 | *RP11-169K16.9* | hypothetical LOC729614 |
| gen660 | 1553934_at | NR_027245 | *C18orf20* | ENSG00000179676 | *NCRNA00305* | chromosome 18 open reading frame 20 |
| gen661 | 1564444_at | NR_024564 | *LOC100130264* | ENSG00000179447 | *RP5-1027G4.3* | hypothetical LOC100130264 |
| gen662 | 235397_at | NR_026873 | *NCRNA00174* | ENSG00000179406 | *NCRNA00174* | non-protein coding RNA 174 |
| gen663 | 242649_x_at | NR_022014 | *C15orf21* | ENSG00000179362 | *HMGN2P46* | Dresden prostate cancer 2 |
| gen664 | 230012_at | NR_026951 | *C17orf44* | ENSG00000178977 | *NCRNA00324* | chromosome 17 open reading frame 44 |
| gen665 | 227909_at | NR_024359 /// NR_024493 | *NCRNA00086 /// NCRNA00087* | ENSG00000178947 /// ENSG00000196972 | *NCRNA00086* | non-protein coding RNA 86 /// non-protein coding RNA 87 |
| gen666 | 1553519_at | NR_027246 | *C21orf94* | ENSG00000178457 | *NCRNA00314* | chromosome 21 open reading frame 94 |
| gen667 | 232446_at | NR_027107 | *MGC45800* | ENSG00000177822 | *AC108142.1* | hypothetical LOC90768 |
| gen668 | 1556630_at | NR_026939 /// NR_026940 /// NR_026941 | *CASC2* | ENSG00000177640 | *CASC2* | cancer susceptibility candidate 2 |
| gen669 | 1562336_at | NR_026939 /// NR_026940 /// NR_026941 | *CASC2* | ENSG00000177640 | *CASC2* | cancer susceptibility candidate 2 |
| gen670 | 1564371_a_at | NR_026939 /// NR_026940 /// NR_026941 | *CASC2* | ENSG00000177640 | *CASC2* | cancer susceptibility candidate 2 |
| gen671 | 1564372_s_at | NR_026939 /// NR_026940 /// NR_026941 | *CASC2* | ENSG00000177640 | *CASC2* | cancer susceptibility candidate 2 |
| gen672 | 239713_at | NR_026939 /// NR_026940 /// NR_026941 | *CASC2* | ENSG00000177640 | *CASC2* | cancer susceptibility candidate 2 |
| gen673 | 224915_x_at | NR_003604 /// NR_003605 /// NR_003606 /// NR_036658 /// NR_036659 | *NCRNA00275* | ENSG00000177410 | *NCRNA00275* | non-protein coding RNA 275 |
| gen674 | 226227_x_at | NR_003604 /// NR_003605 /// NR_003606 /// NR_036658 /// NR_036659 | *NCRNA00275* | ENSG00000177410 | *NCRNA00275* | non-protein coding RNA 275 |
| gen675 | 226835_s_at | NR_003604 /// NR_003605 /// NR_003606 /// NR_036658 /// NR_036659 | *NCRNA00275* | ENSG00000177410 | *NCRNA00275* | non-protein coding RNA 275 |
| gen676 | 231275_at | NR_015440 /// NR_024371 | *FLJ42875* | ENSG00000177133 | *RP1-163G9.1* | hypothetical LOC440556 |
| gen677 | 236360_at | NR_015440 /// NR_024371 | *FLJ42875* | ENSG00000177133 | *RP1-163G9.1* | hypothetical LOC440556 |
| gen678 | 237108_x_at | NR_015440 /// NR_024371 | *FLJ42875* | ENSG00000177133 | *RP1-163G9.1* | hypothetical LOC440556 |
| gen679 | 242269_at | NR_015440 /// NR_024371 | *FLJ42875* | ENSG00000177133 | *RP1-163G9.1* | hypothetical LOC440556 |
| gen680 | 231595_at | NR_034093 /// NR_034094 | *LOC100129827* | ENSG00000177112 | *RP11-58H20.3* | hypothetical LOC100129827 |
| gen681 | 223913_s_at | NR_027148 | *C19orf30* | ENSG00000176840 | *NCRNA00306* | chromosome 19 open reading frame 30 |
| gen682 | 207063_at | NR_001544 | *NCRNA00185* | ENSG00000176728 | *TTTY14* | non-protein coding RNA 185 |
| gen683 | 224003_at | NR_001543 | *TTTY14* | ENSG00000176728 | *TTTY14* | testis-specific transcript, Y-linked 14 (non-protein coding) |
| gen684 | 1553448_at | NR_027060 | *FLJ34503* | ENSG00000175967 | *RP11-544L8__B.4* | hypothetical FLJ34503 |
| gen685 | 235083_at | NR_027244 | *LOC151009* | ENSG00000175772 /// ENSG00000204588 | *AC112229.7* | hypothetical LOC151009 |
| gen686 | 228614_at | NR_027063 | *NCRNA00116* | ENSG00000175701 | *NCRNA00116* | non-protein coding RNA 116 |
| gen687 | 1553053_at | NR_024182 /// NR_024183 /// NR_024184 | *C14orf48* | ENSG00000175699 | *C14orf48* | chromosome 14 open reading frame 48 |
| gen688 | 1570111_at | NR_024182 /// NR_024183 /// NR_024184 | *C14orf48* | ENSG00000175699 | *C14orf48* | chromosome 14 open reading frame 48 |
| gen689 | 1557788_a_at | NR_023389 /// NR_023390 | *C9orf130* | ENSG00000175611 | *C9orf130* | chromosome 9 open reading frame 130 |
| gen690 | 227893_at | NR_023389 /// NR_023390 | *C9orf130* | ENSG00000175611 | *C9orf130* | chromosome 9 open reading frame 130 |
| gen691 | 234429_at | NR_023389 /// NR_023390 | *C9orf130* | ENSG00000175611 | *C9orf130* | chromosome 9 open reading frame 130 |
| gen692 | 225065_x_at | NR_027158 /// NR_027159 /// NR_027160 /// NR_027161 /// NR_027162 /// NR_027163 /// NR_027164 /// NR_027165 /// NR_027166 /// NR_027167 /// NR_027168 /// NR_027169 /// NR_027170 /// NR_027171 /// NR_027172 /// NR_027173 /// NR_027174 /// NR_027175 /// NR_027176 /// NR_027177 /// NR_027178 /// NR_027179 /// NR_027667 | *NCRNA00188* | ENSG00000175061 | *NCRNA00188* | non-protein coding RNA 188 |
| gen693 | 239754_at | NR_027158 /// NR_027159 /// NR_027160 /// NR_027161 /// NR_027162 /// NR_027163 /// NR_027164 /// NR_027165 /// NR_027166 /// NR_027167 /// NR_027168 /// NR_027169 /// NR_027170 /// NR_027171 /// NR_027172 /// NR_027173 /// NR_027174 /// NR_027175 /// NR_027176 /// NR_027177 /// NR_027178 /// NR_027179 /// NR_027667 | *NCRNA00188* | ENSG00000175061 | *NCRNA00188* | non-protein coding RNA 188 |
| gen694 | 1554203_at | NR_027021 | *GRIK1-AS* | ENSG00000174680 | *GRIK1-AS1* | GRIK1 antisense RNA (non-protein coding) |
| gen695 | 1553204_at | NR_033263 | *C20orf200* | ENSG00000174403 | *C20orf200* | chromosome 20 open reading frame 200 |
| gen696 | 225799_at | NR_015395 /// NR_024204 /// NR_024205 /// NR_024206 /// NR_024373 | *LOC541471 /// NCRNA00152* | ENSG00000172965 /// ENSG00000222041 | *AC068491.1* | hypothetical LOC541471 /// non-protein coding RNA 152 |
| gen697 | 241418_at | NR_033752 | *LOC344887* | ENSG00000171658 | *RP11-443P15.2* | Similar to hCG2041270 |
| gen698 | 1563983_at | NR_024458 | *LOC100190939* | ENSG00000170919 | *XXyac-R12DG2.2* | Hypothetical LOC100190939 |
| gen699 | 226407_at | NR_024458 | *LOC100190939* | ENSG00000170919 | *XXyac-R12DG2.2* | hypothetical LOC100190939 |
| gen700 | 227709_at | NR_024458 | *LOC100190939* | ENSG00000170919 | *XXyac-R12DG2.2* | hypothetical LOC100190939 |
| gen701 | 227710_s_at | NR_024458 | *LOC100190939* | ENSG00000170919 | *XXyac-R12DG2.2* | hypothetical LOC100190939 |
| gen702 | 228913_at | NR_024458 | *LOC100190939* | ENSG00000170919 | *XXyac-R12DG2.2* | hypothetical LOC100190939 |
| gen703 | 225391_at | NR_015433 | *LOC93622* | ENSG00000170846 | *AC093323.3* | hypothetical LOC93622 |
| gen704 | 207259_at | NR_024626 | *C17orf73* | ENSG00000167117 | *C17orf73* | chromosome 17 open reading frame 73 |
| gen705 | 220263_at | NR_026763 | *SMAD5OS* | ENSG00000164621 | *SMAD5-AS1* | SMAD family member 5 opposite strand |
| gen706 | 1553493_a_at | NR_001578 | *TDH* | ENSG00000154316 | *TDH* | L-threonine dehydrogenase |
| gen707 | 1555151_s_at | NR_001578 | *TDH* | ENSG00000154316 | *TDH* | L-threonine dehydrogenase |
| gen708 | 224443_at | NR_026761 | *C1orf97* | ENSG00000153363 | *C1orf97* | chromosome 1 open reading frame 97 |
| gen709 | 224444_s_at | NR_026761 | *C1orf97* | ENSG00000153363 | *C1orf97* | chromosome 1 open reading frame 97 |
| gen710 | 205833_s_at | NR_024617 /// NR_028508 /// NR_028509 | *PART1* | ENSG00000152931 | *PART1* | prostate androgen-regulated transcript 1 (non-protein coding) |
| gen711 | 205834_s_at | NR_024617 /// NR_028508 /// NR_028509 | *PART1* | ENSG00000152931 | *PART1* | prostate androgen-regulated transcript 1 (non-protein coding) |
| gen712 | 233151_s_at | NR_001534 | *TTTY7* | ENSG00000147753 /// ENSG00000147761 | *TTTY7* | testis-specific transcript, Y-linked 7 (non-protein coding) |
| gen713 | 234309_at | NR_001534 | *TTTY7* | ENSG00000147753 /// ENSG00000147761 | *TTTY7* | testis-specific transcript, Y-linked 7 (non-protein coding) |
| gen714 | 219627_at | NR_027788 /// NR_027789 | *ZNF767* | ENSG00000133624 | *ZNF767* | zinc finger family member 767 |
| gen715 | 220449_at | NR_034104 | *LOC79015* | ENSG00000132832 | *RP11-445H22.3* | hypothetical LOC79015 |
| gen716 | 219442_at | NR_024034 | *KIAA0664L3* | ENSG00000131797 | *KIAA0664L3* | KIAA0664-like 3 |
| gen717 | 224041_at | NR_001527 | *TTTY6* | ENSG00000131538 /// ENSG00000131548 | *TTTY6* | testis-specific transcript, Y-linked 6 (non-protein coding) |
| gen718 | 211460_at | NR_001530 /// NR_002159 | *TTTY9A /// TTTY9B* | ENSG00000131007 /// ENSG00000131009 | *TTTY9B* | testis-specific transcript, Y-linked 9A (non-protein coding) /// testis-specific transcript, Y-linked 9B (non-protein coding) |
| gen719 | 224646_x_at | NR_002196 | *H19* | ENSG00000130600 | *H19* | H19, imprinted maternally expressed transcript (non-protein coding) |
| gen720 | 224997_x_at | NR_002196 | *H19* | ENSG00000130600 | *H19* | H19, imprinted maternally expressed transcript (non-protein coding) |
| gen721 | 216673_at | NR_001538 /// NR_003589 | *TTTY1 /// TTTY1B* | ENSG00000129816 /// ENSG00000129845 | *TTTY1B* | testis-specific transcript, Y-linked 1 (non-protein coding) /// testis-specific transcript, Y-linked 1B (non-protein coding) |
| gen722 | 231760_at | NR_028295 | *NCRNA00029* | ENSG00000125514 | *NCRNA00029* | non-protein coding RNA 29 |
| gen723 | 1554880_at | NR_026882 | *DKFZP434K028* | ENSG00000124915 | *RP11-467L20.10* | hypothetical LOC26070 |
| gen724 | 208585_at | NR_027795 | *BTN2A3* | ENSG00000124549 /// ENSG00000254005 | *BTN2A3* | butyrophilin, subfamily 2, member A3 |
| gen725 | 206279_at | NR_028062 | *PRKY* | ENSG00000099725 | *PRKY* | protein kinase, Y-linked |
| gen726 | 1552955_at | --- |  | ENSG00000170983 | *NCRNA00208* |  |
| gen727 | 1553024_at | --- |  | ENSG00000232307 | *DAOA-AS1* |  |
| gen728 | 1553269_at | --- |  | ENSG00000250312 | *ZNF718* |  |
| gen729 | 1553457_at | --- |  | ENSG00000215162 | *NCRNA00269* |  |
| gen730 | 1553882_at | --- |  | ENSG00000248360 | *RP11-458B24.2* |  |
| gen731 | 1553935_at | --- |  | ENSG00000249993 | *RP11-91K8.1* |  |
| gen732 | 1553936_a_at | --- |  | ENSG00000249993 | *RP11-91K8.1* |  |
| gen733 | 1554781_at | --- |  | ENSG00000250328 | *CTC-210G5.1* |  |
| gen734 | 1554887_at | --- |  | ENSG00000232445 | *RP11-132A1.4* |  |
| gen735 | 1554946_at | --- |  | ENSG00000249550 | *RP11-438N16.1* |  |
| gen736 | 1554949_at | --- |  | ENSG00000231010 | *RP6-109B7.2* |  |
| gen737 | 1554983_at | --- |  | ENSG00000238265 | *NCRNA00317* |  |
| gen738 | 1555085_at | --- |  | ENSG00000224718 | *RP11-222A5.1* |  |
| gen739 | 1555132_at | --- |  | ENSG00000196893 | *AC090286.4* |  |
| gen740 | 1555135_at | --- |  | ENSG00000246025 | *AL355344.1* |  |
| gen741 | 1555224_at | --- |  | ENSG00000227480 | *AC069146.2* |  |
| gen742 | 1555344_at | --- |  | ENSG00000253656 | *KB-1568E2.1* |  |
| gen743 | 1555580_at | --- |  | ENSG00000253984 | *CTD-3056O22.1* |  |
| gen744 | 1555868_at | --- |  | ENSG00000236013 | *RP3-332B22.1* |  |
| gen745 | 1555869_a_at | --- |  | ENSG00000236013 | *RP3-332B22.1* |  |
| gen746 | 1555893_at | --- |  | ENSG00000254231 | *CTD-2284J15.1* |  |
| gen747 | 1555994_at | --- |  | ENSG00000227528 | *DIAPH3-AS1* |  |
| gen748 | 1556007_s_at | --- |  | ENSG00000230551 | *CTB-89H12.4* |  |
| gen749 | 1556043_a_at | --- |  | ENSG00000237298 | *AC009948.3* |  |
| gen750 | 1556048_at | --- |  | ENSG00000226754 | *RP5-1024G6.5* |  |
| gen751 | 1556064_at | --- |  | ENSG00000247670 | *AL078611.1* |  |
| gen752 | 1556161_a_at | --- |  | ENSG00000251616 | *RP11-485M7.3* |  |
| gen753 | 1556235_at | --- |  | ENSG00000227733 | *RP4-565E6.1* |  |
| gen754 | 1556247_a_at | --- |  | ENSG00000237862 | *RP1-63G5.7* |  |
| gen755 | 1556364_at | --- |  | ENSG00000241684 | *CTD-2230D16.1* |  |
| gen756 | 1556387_at | --- |  | ENSG00000243818 | *RP11-372E1.4* |  |
| gen757 | 1556388_a_at | --- |  | ENSG00000243818 | *RP11-372E1.4* |  |
| gen758 | 1556400_at | --- |  | ENSG00000233542 | *RP11-547D24.1* |  |
| gen759 | 1556401_a_at | --- |  | ENSG00000233542 | *RP11-547D24.1* |  |
| gen760 | 1556422_at | --- |  | ENSG00000245443 | *AC005498.1* |  |
| gen761 | 1556436_at | --- |  | ENSG00000246339 | *CTD-2172A10.1* |  |
| gen762 | 1556453_at | --- |  | ENSG00000229727 | *AC013460.1* |  |
| gen763 | 1556454_a_at | --- |  | ENSG00000229727 | *AC013460.1* |  |
| gen764 | 1556468_at | --- |  | ENSG00000249385 | *AL445670.1* |  |
| gen765 | 1556469_s_at | --- |  | ENSG00000249385 | *AL445670.1* |  |
| gen766 | 1556494_at | --- |  | ENSG00000250772 | *RP11-236P13.1* |  |
| gen767 | 1556507_at | --- |  | ENSG00000239513 | *RP11-2A4.3* |  |
| gen768 | 1556508_s_at | --- |  | ENSG00000239513 | *RP11-2A4.3* |  |
| gen769 | 1556511_a_at | --- |  | ENSG00000237928 | *RP4-668G5.1* |  |
| gen770 | 1556518_at | --- |  | ENSG00000227375 | *AC092937.2* |  |
| gen771 | 1556617_a_at | --- |  | ENSG00000242385 | *RP11-263A24.1* |  |
| gen772 | 1556645_s_at | --- |  | ENSG00000245025 | *RP11-875O11.1* |  |
| gen773 | 1556662_at | --- |  | ENSG00000250116 | *RP11-417F21.1* |  |
| gen774 | 1556663_s_at | --- |  | ENSG00000250116 | *RP11-417F21.1* |  |
| gen775 | 1556670_at | --- |  | ENSG00000234945 | *AC109828.1* |  |
| gen776 | 1556671_s_at | --- |  | ENSG00000234945 | *AC109828.1* |  |
| gen777 | 1556675_s_at | --- |  | ENSG00000249346 | *RP3-468B3.3* |  |
| gen778 | 1556717_at | --- |  | ENSG00000229719 | *AP001187.9* |  |
| gen779 | 1556718_s_at | --- |  | ENSG00000229719 | *AP001187.9* |  |
| gen780 | 1556734_at | --- |  | ENSG00000228444 | *LMO7-AS1* |  |
| gen781 | 1556775_at | --- |  | ENSG00000226622 | *AC092155.4* |  |
| gen782 | 1556776_a_at | --- |  | ENSG00000247585 | *AC008567.1* |  |
| gen783 | 1556777_a_at | --- |  | ENSG00000247585 | *AC008567.1* |  |
| gen784 | 1556789_a_at | --- |  | ENSG00000238197 | *GCFC1-AS1* |  |
| gen785 | 1556796_at | --- |  | ENSG00000206552 | *RP11-141M3.3* |  |
| gen786 | 1556851_at | --- |  | ENSG00000247011 | *RP11-700H6.1* |  |
| gen787 | 1556852_a_at | --- |  | ENSG00000247011 | *RP11-700H6.1* |  |
| gen788 | 1556899_at | --- |  | ENSG00000237054 | *AL132780.10* |  |
| gen789 | 1556903_at | --- |  | ENSG00000236064 | *RP6-191P20.4* |  |
| gen790 | 1556911_at | --- |  | ENSG00000230002 | *AC074008.4* |  |
| gen791 | 1556916_a_at | --- |  | ENSG00000248529 | *RP11-2O17.2* |  |
| gen792 | 1556917_a_at | --- |  | ENSG00000248529 | *RP11-2O17.2* |  |
| gen793 | 1556921_at | --- |  | ENSG00000227599 | *RP4-753D4.2* |  |
| gen794 | 1556924_at | --- |  | ENSG00000226312 | *CFLAR-AS1* |  |
| gen795 | 1556931_at | --- |  | ENSG00000230068 | *RP1-224A6.6* |  |
| gen796 | 1556936_at | --- |  | ENSG00000226237 | *RP11-276H19.1* |  |
| gen797 | 1556944_at | --- |  | ENSG00000236656 | *RP11-144L1.4* |  |
| gen798 | 1556945_a_at | --- |  | ENSG00000236656 | *RP11-144L1.4* |  |
| gen799 | 1556992_at | --- |  | ENSG00000248049 | *RP11-453E17.1* |  |
| gen800 | 1557004_at | --- |  | ENSG00000230102 | *RP11-407B7.1* |  |
| gen801 | 1557017_at | --- |  | ENSG00000245070 | *AC068765.1* |  |
| gen802 | 1557018_a_at | --- |  | ENSG00000245070 | *AC068765.1* |  |
| gen803 | 1557022_at | --- |  | ENSG00000245832 | *RP11-179A16.1* |  |
| gen804 | 1557026_at | --- |  | ENSG00000228044 | *RP4-781K5.4* |  |
| gen805 | 1557050_at | --- |  | ENSG00000233429 | *HOTAIRM1* |  |
| gen806 | 1557051_s_at | --- |  | ENSG00000233429 | *HOTAIRM1* |  |
| gen807 | 1557060_at | --- |  | ENSG00000236719 | *RP11-522D2.1* |  |
| gen808 | 1557117_at | --- |  | ENSG00000236778 | *RP11-550E22.3* |  |
| gen809 | 1557135_at | --- |  | ENSG00000235560 | *AC002310.1* |  |
| gen810 | 1557139_at | --- |  | ENSG00000245614 | *RP11-551L14.5* |  |
| gen811 | 1557149_at | --- |  | ENSG00000226900 | *RP11-432J24.5* |  |
| gen812 | 1557177_at | --- |  | ENSG00000251364 | *CTD-2516F10.2* |  |
| gen813 | 1557214_at | --- |  | ENSG00000250968 | *RP11-322J23.1* |  |
| gen814 | 1557219_at | --- |  | ENSG00000251350 | *RP11-328N19.1* |  |
| gen815 | 1557274_at | --- |  | ENSG00000238273 | *AC012360.6* |  |
| gen816 | 1557276_at | --- |  | ENSG00000249346 | *RP3-468B3.3* |  |
| gen817 | 1557277_a_at | --- |  | ENSG00000249346 | *RP3-468B3.3* |  |
| gen818 | 1557287_at | --- |  | ENSG00000231971 | *RP11-557H15.3* |  |
| gen819 | 1557336_at | --- |  | ENSG00000241316 | *RP11-81N13.1* |  |
| gen820 | 1557338_x_at | --- |  | ENSG00000241316 | *RP11-81N13.1* |  |
| gen821 | 1557386_at | --- |  | ENSG00000226806 | *AC011893.3* |  |
| gen822 | 1557424_at | --- |  | ENSG00000250544 | *CTC-493L21.1* |  |
| gen823 | 1557427_at | --- |  | ENSG00000247843 | *AC022916.1* |  |
| gen824 | 1557443_s_at | --- |  | ENSG00000250427 | *CTD-3179P9.2* |  |
| gen825 | 1557484_at | --- |  | ENSG00000235749 | *RP11-634B7.4* |  |
| gen826 | 1557486_at | --- |  | ENSG00000236977 | *AC010746.2* |  |
| gen827 | 1557491_at | --- |  | ENSG00000225488 | *RP11-760D2.1* |  |
| gen828 | 1557493_x_at | --- |  | ENSG00000225488 | *RP11-760D2.1* |  |
| gen829 | 1557498_a_at | --- |  | ENSG00000241336 | *RP11-451G4.1* |  |
| gen830 | 1557507_at | --- |  | ENSG00000248275 | *CTC-338M12.3* |  |
| gen831 | 1557540_at | --- |  | ENSG00000253123 | *RP11-527N22.1* |  |
| gen832 | 1557564_at | --- |  | ENSG00000244926 | *RP11-613D13.7* |  |
| gen833 | 1557565_a_at | --- |  | ENSG00000244926 | *RP11-613D13.7* |  |
| gen834 | 1557566_at | --- |  | ENSG00000244861 | *AC091609.1* |  |
| gen835 | 1557570_a_at | --- |  | ENSG00000231453 | *AC018470.4* |  |
| gen836 | 1557598_at | --- |  | ENSG00000234715 | *CTB-107G13.1* |  |
| gen837 | 1557599_a_at | --- |  | ENSG00000234715 | *CTB-107G13.1* |  |
| gen838 | 1557604_at | --- |  | ENSG00000238033 | *AC002480.2* |  |
| gen839 | 1557605_a_at | --- |  | ENSG00000238033 | *AC002480.2* |  |
| gen840 | 1557606_at | --- |  | ENSG00000247131 | *RP11-588G21.2* |  |
| gen841 | 1557610_at | --- |  | ENSG00000237399 | *RP11-298E9.3* |  |
| gen842 | 1557646_at | --- |  | ENSG00000247502 | *AL442203.1* |  |
| gen843 | 1557647_a_at | --- |  | ENSG00000247502 | *AL442203.1* |  |
| gen844 | 1557652_a_at | --- |  | ENSG00000173811 | *RP4-613B23.4* |  |
| gen845 | 1557692_a_at | --- |  | ENSG00000230288 | *RP11-519G16.1* |  |
| gen846 | 1557702_at | --- |  | ENSG00000223393 | *RP5-858B6.3* |  |
| gen847 | 1557731_at | --- |  | ENSG00000234721 | *AC125421.1* |  |
| gen848 | 1557733_a_at | --- |  | ENSG00000233355 | *RP11-343J24.1* |  |
| gen849 | 1557753_at | --- |  | ENSG00000246876 | *RP11-519M16.1* |  |
| gen850 | 1557776_at | --- |  | ENSG00000236155 | *RP11-231P20.2* |  |
| gen851 | 1557779_at | --- |  | ENSG00000231131 | *RP11-346D6.6* |  |
| gen852 | 1557787_at | --- |  | ENSG00000249521 | *CTD-2161F6.3* |  |
| gen853 | 1557802_at | --- |  | ENSG00000231816 | *RP4-782L23.1* |  |
| gen854 | 1557863_at | --- |  | ENSG00000246740 | *CTD-2382E5.1* |  |
| gen855 | 1557864_x_at | --- |  | ENSG00000246740 | *CTD-2382E5.1* |  |
| gen856 | 1557865_at | --- |  | ENSG00000246422 | *CTD-2024I7.13* |  |
| gen857 | 1557873_at | --- |  | ENSG00000247199 | *RP11-373N22.3* |  |
| gen858 | 1557878_at | --- |  | ENSG00000245213 | *RP11-10K16.1* |  |
| gen859 | 1557881_at | --- |  | ENSG00000223910 | *C10orf44* |  |
| gen860 | 1557896_at | --- |  | ENSG00000224481 | *RP11-495P10.3* |  |
| gen861 | 1558168_at | --- |  | ENSG00000206417 | *RP13-685P2.5* |  |
| gen862 | 1558320_at | --- |  | ENSG00000243491 | *RP11-521D12.5* |  |
| gen863 | 1558354_s_at | --- |  | ENSG00000226648 | *RP1-1J6.2* |  |
| gen864 | 1558402_at | --- |  | ENSG00000249201 | *CTD-3080P12.3* |  |
| gen865 | 1558440_at | --- |  | ENSG00000246792 | *RP11-68L18.1* |  |
| gen866 | 1558474_at | --- |  | ENSG00000232611 | *RP11-1114A5.4* |  |
| gen867 | 1558496_at | --- |  | ENSG00000241696 | *RP11-420J11.2* |  |
| gen868 | 1558525_at | --- |  | ENSG00000250616 | *AC012645.1* |  |
| gen869 | 1558529_s_at | --- |  | ENSG00000228463 | *AP006222.2* |  |
| gen870 | 1558588_at | --- |  | ENSG00000232151 /// ENSG00000235398 /// ENSG00000236943 | *RP11-277L2.2* |  |
| gen871 | 1558595_at | --- |  | ENSG00000229127 | *AC007038.7* |  |
| gen872 | 1558660_at | --- |  | ENSG00000224382 | *RP11-34C15.1* |  |
| gen873 | 1558661_at | --- |  | ENSG00000247276 | *AL031780.1* |  |
| gen874 | 1558666_at | --- |  | ENSG00000224204 | *Y10196.2* |  |
| gen875 | 1558672_at | --- |  | ENSG00000244967 | *AL360001.1* |  |
| gen876 | 1558720_at | --- |  | ENSG00000246451 | *AL049840.2* |  |
| gen877 | 1558795_at | --- |  | ENSG00000230910 | *RP3-525N10.2* |  |
| gen878 | 1558819_at | --- |  | ENSG00000246528 | *RP11-159H10.3* |  |
| gen879 | 1558830_at | --- |  | ENSG00000228609 | *RP3-341D10.4* |  |
| gen880 | 1558871_at | --- |  | ENSG00000251531 | *AC097470.1* |  |
| gen881 | 1559002_at | --- |  | ENSG00000231154 | *RP5-1055C14.7* |  |
| gen882 | 1559009_at | --- |  | ENSG00000247694 | *AL136040.2* |  |
| gen883 | 1559110_at | --- |  | ENSG00000226852 | *RP1-212P9.2* |  |
| gen884 | 1559131_a_at | --- |  | ENSG00000247225 | *AL031123.1* |  |
| gen885 | 1559141_s_at | --- |  | ENSG00000177757 /// ENSG00000182366 | *FAM87B* |  |
| gen886 | 1559148_at | --- |  | ENSG00000228671 | *RP11-109A6.4* |  |
| gen887 | 1559169_at | --- |  | ENSG00000233478 | *RP1-187B23.1* |  |
| gen888 | 1559205_s_at | --- |  | ENSG00000245954 | *RP11-18H21.1* |  |
| gen889 | 1559213_at | --- |  | ENSG00000253898 | *RP11-51M18.1* |  |
| gen890 | 1559220_at | --- |  | ENSG00000243224 | *RP5-1157M23.2* |  |
| gen891 | 1559221_at | --- |  | ENSG00000228495 | *RP3-523C21.1* |  |
| gen892 | 1559237_a_at | --- |  | ENSG00000247970 | *AL160313.1* |  |
| gen893 | 1559240_at | --- |  | ENSG00000246090 | *RP11-696N14.1* |  |
| gen894 | 1559280_a_at | --- |  | ENSG00000248329 | *RP11-366M4.3* |  |
| gen895 | 1559288_at | --- |  | ENSG00000236451 | *AC067956.1* |  |
| gen896 | 1559296_at | --- |  | ENSG00000241684 | *CTD-2230D16.1* |  |
| gen897 | 1559316_at | --- |  | ENSG00000251629 | *RP11-774D14.1* |  |
| gen898 | 1559321_at | --- |  | ENSG00000249364 | *RP11-434D9.1* |  |
| gen899 | 1559333_at | --- |  | ENSG00000228723 | *AC037193.1* |  |
| gen900 | 1559344_at | --- |  | ENSG00000250062 | *RP11-778J15.1* |  |
| gen901 | 1559348_a_at | --- |  | ENSG00000236914 | *AC087473.1* |  |
| gen902 | 1559372_at | --- |  | ENSG00000228397 | *RP1-224A6.3* |  |
| gen903 | 1559470_at | --- |  | ENSG00000228592 | *AP000459.4* |  |
| gen904 | 1559471_s_at | --- |  | ENSG00000228592 | *AP000459.4* |  |
| gen905 | 1559501_at | --- |  | ENSG00000236830 | *AP000689.8* |  |
| gen906 | 1559522_at | --- |  | ENSG00000228697 | *RP5-968D22.1* |  |
| gen907 | 1559540_at | --- |  | ENSG00000251575 | *CTD-2170G1.2* |  |
| gen908 | 1559542_a_at | --- |  | ENSG00000253181 | *RP11-863K10.2* |  |
| gen909 | 1559543_at | --- |  | ENSG00000231473 | *RP11-305D15.2* |  |
| gen910 | 1559605_a_at | --- |  | ENSG00000235997 | *AC109642.1* |  |
| gen911 | 1559617_at | --- |  | ENSG00000224272 | *AC114730.3* |  |
| gen912 | 1559621_at | --- |  | ENSG00000250075 | *RP11-584P21.2* |  |
| gen913 | 1559648_at | --- |  | ENSG00000233093 | *RP3-527F8.2* |  |
| gen914 | 1559654_s_at | --- |  | ENSG00000225083 | *GRTP1-AS1* |  |
| gen915 | 1559655_at | --- |  | ENSG00000204706 | *RP11-195E11.3* |  |
| gen916 | 1559656_a_at | --- |  | ENSG00000204706 | *RP11-195E11.3* |  |
| gen917 | 1559670_at | --- |  | ENSG00000240973 | *RP11-458K10.2* |  |
| gen918 | 1559765_a_at | --- |  | ENSG00000237886 | *RP11-611D20.2* |  |
| gen919 | 1559766_at | --- |  | ENSG00000246022 | *RP11-124N2.3* |  |
| gen920 | 1559787_at | --- |  | ENSG00000234572 | *AC007880.1* |  |
| gen921 | 1559861_at | --- |  | ENSG00000231806 | *RP11-342C23.4* |  |
| gen922 | 1559867_at | --- |  | ENSG00000250041 | *CTD-2003C8.2* |  |
| gen923 | 1559880_at | --- |  | ENSG00000253733 | *RP11-563N12.1* |  |
| gen924 | 1559917_a_at | --- |  | ENSG00000236830 | *AP000689.8* |  |
| gen925 | 1559929_at | --- |  | ENSG00000236751 | *RP1-30G7.2* |  |
| gen926 | 1559939_at | --- |  | ENSG00000204929 | *AC074391.1* |  |
| gen927 | 1559940_s_at | --- |  | ENSG00000204929 | *AC074391.1* |  |
| gen928 | 1559948_at | --- |  | ENSG00000251239 | *CTB-22K21.2* |  |
| gen929 | 1559976_at | --- |  | ENSG00000247498 | *RP11-392P7.8* |  |
| gen930 | 1559986_at | --- |  | ENSG00000244968 | *MIR3650* |  |
| gen931 | 1560240_at | --- |  | ENSG00000230836 | *AC104135.4* |  |
| gen932 | 1560246_at | --- |  | ENSG00000246214 | *RP11-260E18.1* |  |
| gen933 | 1560262_at | --- |  | ENSG00000236304 | *AP001189.4* |  |
| gen934 | 1560282_at | --- |  | ENSG00000249106 | *RP11-806K15.1* |  |
| gen935 | 1560288_at | --- |  | ENSG00000226747 | *AC007966.1* |  |
| gen936 | 1560298_at | --- |  | ENSG00000250668 | *CTD-2296D1.2* |  |
| gen937 | 1560337_at | --- |  | ENSG00000253734 | *RP11-579E24.1* |  |
| gen938 | 1560352_at | --- |  | ENSG00000226688 | *RP11-429G19.2* |  |
| gen939 | 1560383_at | --- |  | ENSG00000248115 | *RP11-752D24.2* |  |
| gen940 | 1560384_a_at | --- |  | ENSG00000248115 | *RP11-752D24.2* |  |
| gen941 | 1560391_at | --- |  | ENSG00000225436 | *AC005971.3* |  |
| gen942 | 1560409_at | --- |  | ENSG00000246813 | *AC012366.1* |  |
| gen943 | 1560425_s_at | --- |  | ENSG00000225706 | *RP11-75C9.1* |  |
| gen944 | 1560431_at | --- |  | ENSG00000231242 | *RP11-87H9.3* |  |
| gen945 | 1560449_at | --- |  | ENSG00000248318 | *RP11-713M15.1* |  |
| gen946 | 1560464_at | --- |  | ENSG00000246747 | *AC139530.1* |  |
| gen947 | 1560491_at | --- |  | ENSG00000241956 | *CTC-340A15.2* |  |
| gen948 | 1560495_at | --- |  | ENSG00000245622 | *AC110079.2* |  |
| gen949 | 1560506_at | --- |  | ENSG00000226953 | *AC010890.1* |  |
| gen950 | 1560520_at | --- |  | ENSG00000225541 | *AC002480.5* |  |
| gen951 | 1560533_at | --- |  | ENSG00000230427 | *RP11-313A24.1* |  |
| gen952 | 1560538_at | --- |  | ENSG00000232377 | *AC016910.1* |  |
| gen953 | 1560548_at | --- |  | ENSG00000229175 | *RP11-501G7.1* |  |
| gen954 | 1560560_at | --- |  | ENSG00000232628 | *RP11-365O16.3* |  |
| gen955 | 1560563_at | --- |  | ENSG00000245109 | *AC024361.1* |  |
| gen956 | 1560565_at | --- |  | ENSG00000249343 | *CTD-2275D24.2* |  |
| gen957 | 1560579_s_at | --- |  | ENSG00000224167 /// ENSG00000226419 | *RP3-522D1.1* |  |
| gen958 | 1560588_at | --- |  | ENSG00000225794 | *AC073321.4* |  |
| gen959 | 1560625_s_at | --- |  | ENSG00000225062 | *AC021016.6* |  |
| gen960 | 1560633_a_at | --- |  | ENSG00000240770 | *NCRNA00285* |  |
| gen961 | 1560634_a_at | --- |  | ENSG00000237479 | *AC007557.2* |  |
| gen962 | 1560679_at | --- |  | ENSG00000250054 | *AC005035.2* |  |
| gen963 | 1560690_at | --- |  | ENSG00000227143 | *RP11-159H3.2* |  |
| gen964 | 1560692_at | --- |  | ENSG00000224223 | *GS1-18A18.1* |  |
| gen965 | 1560716_at | --- |  | ENSG00000248461 | *CTD-2207A17.1* |  |
| gen966 | 1560717_at | --- |  | ENSG00000235731 | *AC124997.1* |  |
| gen967 | 1560725_at | --- |  | ENSG00000244040 | *CTD-2049J23.2* |  |
| gen968 | 1560760_s_at | --- |  | ENSG00000224308 | *RP1-13P20.6* |  |
| gen969 | 1560791_at | --- |  | ENSG00000245928 | *RP11-630D6.5* |  |
| gen970 | 1560797_s_at | --- |  | ENSG00000224216 /// ENSG00000224533 | *RP13-228J13.1* |  |
| gen971 | 1560827_at | --- |  | ENSG00000245975 | *AC090515.1* |  |
| gen972 | 1560833_at | --- |  | ENSG00000229311 | *RP11-475I24.8* |  |
| gen973 | 1560856_at | --- |  | ENSG00000226272 | *AC008533.1* |  |
| gen974 | 1560861_at | --- |  | ENSG00000235300 | *AC090627.1* |  |
| gen975 | 1560862_at | --- |  | ENSG00000230962 | *RP11-113E21.1* |  |
| gen976 | 1560890_at | --- |  | ENSG00000249307 | *RP11-438E5.1* |  |
| gen977 | 1560891_a_at | --- |  | ENSG00000249307 | *RP11-438E5.1* |  |
| gen978 | 1560920_s_at | --- |  | ENSG00000233800 | *RP11-295G24.4* |  |
| gen979 | 1560940_at | --- |  | ENSG00000229558 | *SACS-AS1* |  |
| gen980 | 1560941_a_at | --- |  | ENSG00000229558 | *SACS-AS1* |  |
| gen981 | 1560957_at | --- |  | ENSG00000223659 /// ENSG00000234986 | *RP5-857K21.5* |  |
| gen982 | 1560958_s_at | --- |  | ENSG00000223786 | *RP11-554D15.1* |  |
| gen983 | 1561029_at | --- |  | ENSG00000233997 | *AP000475.2* |  |
| gen984 | 1561033_at | --- |  | ENSG00000226803 | *RP11-203B9.4* |  |
| gen985 | 1561034_at | --- |  | ENSG00000224127 | *RP11-510C10.2* |  |
| gen986 | 1561037_a_at | --- |  | ENSG00000225518 | *RP11-396C23.2* |  |
| gen987 | 1561044_at | --- |  | ENSG00000237292 | *RP11-540K16.1* |  |
| gen988 | 1561045_a_at | --- |  | ENSG00000237292 | *RP11-540K16.1* |  |
| gen989 | 1561052_s_at | --- |  | ENSG00000254129 | *CTD-2647L4.1* |  |
| gen990 | 1561060_at | --- |  | ENSG00000203643 | *AC012456.3* |  |
| gen991 | 1561061_at | --- |  | ENSG00000245729 | *RP11-480D4.1* |  |
| gen992 | 1561069_at | --- |  | ENSG00000229775 | *RP11-298H24.1* |  |
| gen993 | 1561084_at | --- |  | ENSG00000250406 | *RP13-539F13.3* |  |
| gen994 | 1561086_at | --- |  | ENSG00000230393 | *AC092667.2* |  |
| gen995 | 1561091_at | --- |  | ENSG00000228308 | *RP11-255G21.1* |  |
| gen996 | 1561102_at | --- |  | ENSG00000253871 | *RP11-756K15.2* |  |
| gen997 | 1561103_at | --- |  | ENSG00000229913 | *RP11-378I13.1* |  |
| gen998 | 1561110_at | --- |  | ENSG00000246982 | *RP1-179N16.6* |  |
| gen999 | 1561141_at | --- |  | ENSG00000251301 | *RP11-81H14.2* |  |
| gen1000 | 1561210_at | --- |  | ENSG00000249584 | *RP11-478P10.1* |  |
| gen1001 | 1561211_at | --- |  | ENSG00000226252 | *RP1-18D14.7* |  |
| gen1002 | 1561212_at | --- |  | ENSG00000234864 | *AL022344.5* |  |
| gen1003 | 1561213_at | --- |  | ENSG00000233973 | *RP4-598G3.1* |  |
| gen1004 | 1561228_at | --- |  | ENSG00000248872 | *RP11-344G13.1* |  |
| gen1005 | 1561232_at | --- |  | ENSG00000249375 | *RP11-1136L8.1* |  |
| gen1006 | 1561233_at | --- |  | ENSG00000245651 | *RP11-620J15.2* |  |
| gen1007 | 1561237_at | --- |  | ENSG00000245521 | *AL139082.1* |  |
| gen1008 | 1561245_at | --- |  | ENSG00000253894 | *RP11-45K10.2* |  |
| gen1009 | 1561249_a_at | --- |  | ENSG00000246877 | *AC019294.4* |  |
| gen1010 | 1561254_at | --- |  | ENSG00000228742 | *RP5-884M6.1* |  |
| gen1011 | 1561256_at | --- |  | ENSG00000253510 | *RP5-991O23.1* |  |
| gen1012 | 1561266_at | --- |  | ENSG00000244675 | *AC108676.1* |  |
| gen1013 | 1561288_at | --- |  | ENSG00000249917 | *AF130342.1* |  |
| gen1014 | 1561289_at | --- |  | ENSG00000227555 | *RP11-406A20.4* |  |
| gen1015 | 1561290_at | --- |  | ENSG00000226983 | *AP000235.2* |  |
| gen1016 | 1561305_at | --- |  | ENSG00000248210 | *RP11-24I21.1* |  |
| gen1017 | 1561307_at | --- |  | ENSG00000230010 | *RP4-568F9.6* |  |
| gen1018 | 1561309_x_at | --- |  | ENSG00000230010 | *RP4-568F9.6* |  |
| gen1019 | 1561314_at | --- |  | ENSG00000248115 | *RP11-752D24.2* |  |
| gen1020 | 1561323_at | --- |  | ENSG00000250658 | *RP11-138B4.1* |  |
| gen1021 | 1561328_at | --- |  | ENSG00000234474 | *RP11-501J20.2* |  |
| gen1022 | 1561335_at | --- |  | ENSG00000235407 | *RP11-470L19.2* |  |
| gen1023 | 1561340_at | --- |  | ENSG00000234173 | *RP11-257I14.1* |  |
| gen1024 | 1561345_at | --- |  | ENSG00000229699 | *RP1-140J1.1* |  |
| gen1025 | 1561348_at | --- |  | ENSG00000250608 | *RP11-933H2.4* |  |
| gen1026 | 1561352_at | --- |  | ENSG00000229563 | *RP11-245M24.1* |  |
| gen1027 | 1561367_a_at | --- |  | ENSG00000231330 | *AL136962.1* |  |
| gen1028 | 1561369_at | --- |  | ENSG00000243902 | *RP1-63G5.5* |  |
| gen1029 | 1561370_at | --- |  | ENSG00000223522 | *AC093690.1* |  |
| gen1030 | 1561389_at | --- |  | ENSG00000226101 | *AC007461.2* |  |
| gen1031 | 1561398_at | --- |  | ENSG00000234229 | *RP11-308N19.4* |  |
| gen1032 | 1561408_at | --- |  | ENSG00000243321 | *RP11-167H9.3* |  |
| gen1033 | 1561411_at | --- |  | ENSG00000233410 | *RP11-16L9.3* |  |
| gen1034 | 1561413_at | --- |  | ENSG00000249035 | *CTB-113P19.1* |  |
| gen1035 | 1561419_at | --- |  | ENSG00000233360 | *Z83844.1* |  |
| gen1036 | 1561420_a_at | --- |  | ENSG00000233360 | *Z83844.1* |  |
| gen1037 | 1561421_a_at | --- |  | ENSG00000225946 | *RP11-395B7.2* |  |
| gen1038 | 1561427_at | --- |  | ENSG00000236340 | *AC000099.1* |  |
| gen1039 | 1561440_at | --- |  | ENSG00000225028 | *RP11-330M19.1* |  |
| gen1040 | 1561446_at | --- |  | ENSG00000248844 | *RP11-626H12.3* |  |
| gen1041 | 1561448_at | --- |  | ENSG00000241345 | *RP4-630C24.3* |  |
| gen1042 | 1561454_at | --- |  | ENSG00000250819 | *RP11-576E20.1* |  |
| gen1043 | 1561459_at | --- |  | ENSG00000233993 | *RP11-379J5.5* |  |
| gen1044 | 1561460_at | --- |  | ENSG00000233729 | *AC016909.1* |  |
| gen1045 | 1561468_at | --- |  | ENSG00000229702 | *RP1-274L7.1* |  |
| gen1046 | 1561469_at | --- |  | ENSG00000251323 | *RP11-452H21.4* |  |
| gen1047 | 1561471_at | --- |  | ENSG00000247624 | *AC006296.3* |  |
| gen1048 | 1561481_at | --- |  | ENSG00000232837 | *AF064858.7* |  |
| gen1049 | 1561491_at | --- |  | ENSG00000247867 | *CTD-2530H12.1* |  |
| gen1050 | 1561506_at | --- |  | ENSG00000235601 | *RP11-231K24.2* |  |
| gen1051 | 1561516_at | --- |  | ENSG00000228127 | *RP11-12L8.1* |  |
| gen1052 | 1561519_at | --- |  | ENSG00000239454 | *RP11-508O18.1* |  |
| gen1053 | 1561525_at | --- |  | ENSG00000241328 | *RP11-331K15.1* |  |
| gen1054 | 1561527_at | --- |  | ENSG00000237579 | *RP11-324L3.1* |  |
| gen1055 | 1561528_at | --- |  | ENSG00000249345 | *RP11-575F12.1* |  |
| gen1056 | 1561530_at | --- |  | ENSG00000237101 | *RP11-365O16.6* |  |
| gen1057 | 1561531_at | --- |  | ENSG00000239508 | *RP11-117L15.1* |  |
| gen1058 | 1561532_at | --- |  | ENSG00000245598 | *CTB-12A17.1* |  |
| gen1059 | 1561535_at | --- |  | ENSG00000234007 | *AC020601.1* |  |
| gen1060 | 1561539_at | --- |  | ENSG00000246523 | *RP11-736K20.6* |  |
| gen1061 | 1561540_at | --- |  | ENSG00000226620 | *RP11-94P17.1* |  |
| gen1062 | 1561545_at | --- |  | ENSG00000235397 | *AC107982.5* |  |
| gen1063 | 1561546_at | --- |  | ENSG00000225733 | *AC090937.2* |  |
| gen1064 | 1561550_at | --- |  | ENSG00000223646 | *AC002463.3* |  |
| gen1065 | 1561553_at | --- |  | ENSG00000231365 | *RP11-418J17.1* |  |
| gen1066 | 1561554_at | --- |  | ENSG00000227274 | *AC005358.3* |  |
| gen1067 | 1561555_at | --- |  | ENSG00000203601 | *RP11-375F2.2* |  |
| gen1068 | 1561556_at | --- |  | ENSG00000223842 | *RP11-135J2.3* |  |
| gen1069 | 1561558_at | --- |  | ENSG00000236671 | *RP11-573I11.2* |  |
| gen1070 | 1561559_at | --- |  | ENSG00000229494 | *AC012494.1* |  |
| gen1071 | 1561562_at | --- |  | ENSG00000245059 | *AC092718.1* |  |
| gen1072 | 1561567_at | --- |  | ENSG00000251392 | *AL034372.1* |  |
| gen1073 | 1561572_at | --- |  | ENSG00000250198 | *RP11-417J1.4* |  |
| gen1074 | 1561573_at | --- |  | ENSG00000225768 | *RP11-127O4.3* |  |
| gen1075 | 1561575_at | --- |  | ENSG00000225570 | *AC013733.4* |  |
| gen1076 | 1561576_a_at | --- |  | ENSG00000225570 | *AC013733.4* |  |
| gen1077 | 1561577_at | --- |  | ENSG00000232058 | *AC005772.2* |  |
| gen1078 | 1561581_at | --- |  | ENSG00000242622 | *RP11-18H7.1* |  |
| gen1079 | 1561593_at | --- |  | ENSG00000251199 | *RP11-400D2.2* |  |
| gen1080 | 1561605_at | --- |  | ENSG00000236378 | *RP11-394G3.2* |  |
| gen1081 | 1561608_at | --- |  | ENSG00000248176 | *RP11-472K22.1* |  |
| gen1082 | 1561618_at | --- |  | ENSG00000231538 | *AC068542.1* |  |
| gen1083 | 1561625_at | --- |  | ENSG00000237658 | *RP5-968D22.3* |  |
| gen1084 | 1561673_at | --- |  | ENSG00000248804 | *AC087699.1* |  |
| gen1085 | 1561678_at | --- |  | ENSG00000233048 | *RP5-1069C8.2* |  |
| gen1086 | 1561682_at | --- |  | ENSG00000233061 | *RP4-552O12.2* |  |
| gen1087 | 1561699_a_at | --- |  | ENSG00000232684 | *ATP11A-AS1* |  |
| gen1088 | 1561704_at | --- |  | ENSG00000231304 | *AC107622.1* |  |
| gen1089 | 1561712_at | --- |  | ENSG00000239335 | *RP11-745O10.2* |  |
| gen1090 | 1561719_at | --- |  | ENSG00000234597 | *AC010096.1* |  |
| gen1091 | 1561726_s_at | --- |  | ENSG00000238184 | *AC129929.5* |  |
| gen1092 | 1561732_at | --- |  | ENSG00000235643 | *RP1-69M21.2* |  |
| gen1093 | 1561741_at | --- |  | ENSG00000232257 | *RP11-92G12.3* |  |
| gen1094 | 1561757_a_at | --- |  | ENSG00000249196 | *RP11-669N7.2* |  |
| gen1095 | 1561795_at | --- |  | ENSG00000225535 | *AC068610.3* |  |
| gen1096 | 1561877_at | --- |  | ENSG00000229243 | *AC098973.1* |  |
| gen1097 | 1561881_at | --- |  | ENSG00000253658 | *RP11-600K15.1* |  |
| gen1098 | 1561889_at | --- |  | ENSG00000231114 | *AC078842.4* |  |
| gen1099 | 1561909_at | --- |  | ENSG00000254303 | *RP11-398G24.2* |  |
| gen1100 | 1561916_at | --- |  | ENSG00000226581 | *RP11-340I6.8* |  |
| gen1101 | 1561967_at | --- |  | ENSG00000244161 | *RP11-456N14.2* |  |
| gen1102 | 1561983_at | --- |  | ENSG00000242512 | *RP11-416O18.1* |  |
| gen1103 | 1562011_at | --- |  | ENSG00000225572 | *AC003077.1* |  |
| gen1104 | 1562023_at | --- |  | ENSG00000224750 | *RP11-94M14.2* |  |
| gen1105 | 1562029_at | --- |  | ENSG00000250230 | *RP11-855O10.2* |  |
| gen1106 | 1562030_at | --- |  | ENSG00000236858 | *CTA-992D9.6* |  |
| gen1107 | 1562032_at | --- |  | ENSG00000181123 | *RP4-539M6.14* |  |
| gen1108 | 1562044_at | --- |  | ENSG00000246211 | *RP11-632K5.3* |  |
| gen1109 | 1562046_at | --- |  | ENSG00000229051 | *RP5-952N6.1* |  |
| gen1110 | 1562053_at | --- |  | ENSG00000245213 | *RP11-10K16.1* |  |
| gen1111 | 1562056_at | --- |  | ENSG00000250262 | *AC133644.1* |  |
| gen1112 | 1562073_at | --- |  | ENSG00000249694 | *RP11-378A12.1* |  |
| gen1113 | 1562078_at | --- |  | ENSG00000254334 | *RP11-24P4.1* |  |
| gen1114 | 1562081_a_at | --- |  | ENSG00000226722 | *RP11-107B1.1* |  |
| gen1115 | 1562107_at | --- |  | ENSG00000230668 | *XXyac-YR29IB3.1* |  |
| gen1116 | 1562116_at | --- |  | ENSG00000228135 | *AC073128.10* |  |
| gen1117 | 1562121_at | --- |  | ENSG00000234661 | *AC011609.3* |  |
| gen1118 | 1562152_at | --- |  | ENSG00000224788 | *RP5-933E2.1* |  |
| gen1119 | 1562157_at | --- |  | ENSG00000224995 | *RP5-991C6.3* |  |
| gen1120 | 1562190_at | --- |  | ENSG00000229102 | *RP11-360P21.2* |  |
| gen1121 | 1562264_at | --- |  | ENSG00000224715 | *CITF22-49D8.1* |  |
| gen1122 | 1562272_at | --- |  | ENSG00000234210 | *AC006372.4* |  |
| gen1123 | 1562288_at | --- |  | ENSG00000227082 | *AL592494.5* |  |
| gen1124 | 1562341_at | --- |  | ENSG00000233008 | *RP11-475O6.1* |  |
| gen1125 | 1562352_at | --- |  | ENSG00000244738 | *RP11-373E16.3* |  |
| gen1126 | 1562353_x_at | --- |  | ENSG00000244738 | *RP11-373E16.3* |  |
| gen1127 | 1562389_at | --- |  | ENSG00000250241 | *RP11-9G1.3* |  |
| gen1128 | 1562418_at | --- |  | ENSG00000231236 | *AP001604.3* |  |
| gen1129 | 1562449_s_at | --- |  | ENSG00000224973 | *CTA-282F2.3* |  |
| gen1130 | 1562463_at | --- |  | ENSG00000236049 | *AC104777.2* |  |
| gen1131 | 1562474_at | --- |  | ENSG00000231365 | *RP11-418J17.1* |  |
| gen1132 | 1562478_at | --- |  | ENSG00000228705 | *RP5-885L7.10* |  |
| gen1133 | 1562491_at | --- |  | ENSG00000250954 | *RP11-79E3.3* |  |
| gen1134 | 1562509_at | --- |  | ENSG00000251011 | *RP11-402L6.1* |  |
| gen1135 | 1562510_at | --- |  | ENSG00000237290 | *RP11-214L19.1* |  |
| gen1136 | 1562523_at | --- |  | ENSG00000238217 | *AC093590.1* |  |
| gen1137 | 1562540_at | --- |  | ENSG00000250456 | *AC006552.1* |  |
| gen1138 | 1562543_at | --- |  | ENSG00000236372 | *RP5-865N13.1* |  |
| gen1139 | 1562549_at | --- |  | ENSG00000233077 | *RP11-290F20.2* |  |
| gen1140 | 1562568_at | --- |  | ENSG00000247228 | *AC009060.1* |  |
| gen1141 | 1562589_at | --- |  | ENSG00000230523 | *RP3-437I16.1* |  |
| gen1142 | 1562597_at | --- |  | ENSG00000248150 | *RP1-167G20.1* |  |
| gen1143 | 1562604_at | --- |  | ENSG00000234690 | *AC073283.4* |  |
| gen1144 | 1562610_at | --- |  | ENSG00000230333 | *AC004538.3* |  |
| gen1145 | 1562621_at | --- |  | ENSG00000249328 | *RP11-26J3.1* |  |
| gen1146 | 1562623_at | --- |  | ENSG00000248463 | *AC040170.1* |  |
| gen1147 | 1562630_at | --- |  | ENSG00000248529 | *RP11-2O17.2* |  |
| gen1148 | 1562634_at | --- |  | ENSG00000224832 | *AP000469.2* |  |
| gen1149 | 1562638_at | --- |  | ENSG00000250608 | *RP11-933H2.4* |  |
| gen1150 | 1562653_at | --- |  | ENSG00000232900 | *RP4-697P8.3* |  |
| gen1151 | 1562655_at | --- |  | ENSG00000203527 | *Z99756.1* |  |
| gen1152 | 1562661_at | --- |  | ENSG00000224635 | *RP4-564F22.5* |  |
| gen1153 | 1562664_at | --- |  | ENSG00000237870 | *AC073130.1* |  |
| gen1154 | 1562674_at | --- |  | ENSG00000238107 | *RP11-495P10.5* |  |
| gen1155 | 1562690_at | --- |  | ENSG00000248650 | *AL356534.1* |  |
| gen1156 | 1562691_at | --- |  | ENSG00000248464 | *RP11-473L15.2* |  |
| gen1157 | 1562693_at | --- |  | ENSG00000229766 | *RP5-971N18.3* |  |
| gen1158 | 1562697_at | --- |  | ENSG00000246526 | *RP11-539L10.2* |  |
| gen1159 | 1562698_x_at | --- |  | ENSG00000246526 | *RP11-539L10.2* |  |
| gen1160 | 1562699_at | --- |  | ENSG00000247416 | *RP11-629G13.1* |  |
| gen1161 | 1562710_at | --- |  | ENSG00000231079 | *AC105402.4* |  |
| gen1162 | 1562718_at | --- |  | ENSG00000234509 | *AP000253.1* |  |
| gen1163 | 1562737_at | --- |  | ENSG00000249618 | *RP11-422J15.1* |  |
| gen1164 | 1562742_at | --- |  | ENSG00000230817 | *RP4-601K24.1* |  |
| gen1165 | 1562747_at | --- |  | ENSG00000228013 | *RP11-350G8.5* |  |
| gen1166 | 1562786_at | --- |  | ENSG00000248386 | *AC024580.2* |  |
| gen1167 | 1562788_at | --- |  | ENSG00000223823 | *RP11-465B22.5* |  |
| gen1168 | 1562801_at | --- |  | ENSG00000251538 | *RP11-166A12.1* |  |
| gen1169 | 1562802_at | --- |  | ENSG00000231814 | *NCRNA00210* |  |
| gen1170 | 1562810_at | --- |  | ENSG00000229533 | *AC003986.5* |  |
| gen1171 | 1562811_at | --- |  | ENSG00000249326 | *CTD-2194D22.4* |  |
| gen1172 | 1562821_a_at | --- |  | ENSG00000235123 | *AF042090.2* |  |
| gen1173 | 1562822_at | --- |  | ENSG00000235834 | *RP1-60N8.1* |  |
| gen1174 | 1562823_at | --- |  | ENSG00000223949 | *RP11-24J23.2* |  |
| gen1175 | 1562826_at | --- |  | ENSG00000232913 | *RP11-429H9.4* |  |
| gen1176 | 1562841_at | --- |  | ENSG00000230736 | *RP1-149A16.3* |  |
| gen1177 | 1562850_at | --- |  | ENSG00000237764 | *RP11-3B12.2* |  |
| gen1178 | 1562854_at | --- |  | ENSG00000234437 | *RP1-206D15.3* |  |
| gen1179 | 1562860_at | --- |  | ENSG00000236436 | *AC012361.1* |  |
| gen1180 | 1562869_at | --- |  | ENSG00000233399 | *RP11-111I12.1* |  |
| gen1181 | 1562878_at | --- |  | ENSG00000250009 | *AC018797.1* |  |
| gen1182 | 1562891_at | --- |  | ENSG00000251335 | *AL353709.1* |  |
| gen1183 | 1562893_at | --- |  | ENSG00000244791 | *RP11-65D17.1* |  |
| gen1184 | 1562895_at | --- |  | ENSG00000233926 | *RP11-154D17.1* |  |
| gen1185 | 1562902_at | --- |  | ENSG00000203395 | *AC015969.3* |  |
| gen1186 | 1562908_at | --- |  | ENSG00000227016 | *RP11-262K1.1* |  |
| gen1187 | 1562915_at | --- |  | ENSG00000254278 | *RP11-278I4.2* |  |
| gen1188 | 1562916_at | --- |  | ENSG00000230542 | *NCRNA00102* |  |
| gen1189 | 1562918_at | --- |  | ENSG00000251145 | *AL136307.2* |  |
| gen1190 | 1562927_at | --- |  | ENSG00000249094 | *RP1-7G5.6* |  |
| gen1191 | 1562943_at | --- |  | ENSG00000235914 | *MACROD2-AS1* |  |
| gen1192 | 1562960_at | --- |  | ENSG00000229414 | *KCNQ1OT1* |  |
| gen1193 | 1562965_at | --- |  | ENSG00000251517 | *RP11-109E24.1* |  |
| gen1194 | 1562973_at | --- |  | ENSG00000236513 | *RP11-707P20.1* |  |
| gen1195 | 1562982_at | --- |  | ENSG00000238284 | *AC027269.2* |  |
| gen1196 | 1562997_a_at | --- |  | ENSG00000239482 | *RP11-90K6.1* |  |
| gen1197 | 1563005_at | --- |  | ENSG00000230550 | *RP11-74C13.3* |  |
| gen1198 | 1563007_at | --- |  | ENSG00000246084 | *AL445885.1* |  |
| gen1199 | 1563027_at | --- |  | ENSG00000225539 | *AC018799.1* |  |
| gen1200 | 1563038_at | --- |  | ENSG00000228560 | *RP11-550P17.5* |  |
| gen1201 | 1563039_at | --- |  | ENSG00000224973 | *CTA-282F2.3* |  |
| gen1202 | 1563040_s_at | --- |  | ENSG00000224973 | *CTA-282F2.3* |  |
| gen1203 | 1563048_at | --- |  | ENSG00000222044 | *RP5-1039K5.16* |  |
| gen1204 | 1563057_at | --- |  | ENSG00000247006 | *AC106731.1* |  |
| gen1205 | 1563059_at | --- |  | ENSG00000236091 | *RP3-473B4.3* |  |
| gen1206 | 1563069_at | --- |  | ENSG00000236332 | *AP001605.4* |  |
| gen1207 | 1563072_at | --- |  | ENSG00000251511 | *RP11-171N4.1* |  |
| gen1208 | 1563073_at | --- |  | ENSG00000236747 | *RP11-157D23.1* |  |
| gen1209 | 1563074_at | --- |  | ENSG00000242042 | *RP11-690C23.2* |  |
| gen1210 | 1563078_at | --- |  | ENSG00000229720 | *RP3-495K2.2* |  |
| gen1211 | 1563099_at | --- |  | ENSG00000246090 | *RP11-696N14.1* |  |
| gen1212 | 1563103_at | --- |  | ENSG00000232913 | *RP11-429H9.4* |  |
| gen1213 | 1563107_at | --- |  | ENSG00000233215 | *AP000472.2* |  |
| gen1214 | 1563116_at | --- |  | ENSG00000251329 | *RP11-240A16.1* |  |
| gen1215 | 1563127_at | --- |  | ENSG00000228888 | *RP4-764O22.2* |  |
| gen1216 | 1563142_at | --- |  | ENSG00000240687 | *RP11-521D12.1* |  |
| gen1217 | 1563143_at | --- |  | ENSG00000250748 | *RP11-230G5.2* |  |
| gen1218 | 1563157_at | --- |  | ENSG00000244203 | *RP11-905F6.1* |  |
| gen1219 | 1563165_at | --- |  | ENSG00000250546 | *RP11-8L2.1* |  |
| gen1220 | 1563168_at | --- |  | ENSG00000253974 | *RP11-275E10.1* |  |
| gen1221 | 1563170_at | --- |  | ENSG00000251542 | *CTC-313D10.1* |  |
| gen1222 | 1563171_at | --- |  | ENSG00000231373 | *RP11-466A17.1* |  |
| gen1223 | 1563185_at | --- |  | ENSG00000235885 | *AC023115.3* |  |
| gen1224 | 1563188_at | --- |  | ENSG00000223587 | *AY269186.1* |  |
| gen1225 | 1563219_at | --- |  | ENSG00000245711 | *CTD-2320O4.2* |  |
| gen1226 | 1563235_at | --- |  | ENSG00000236230 | *RP11-400N13.1* |  |
| gen1227 | 1563245_at | --- |  | ENSG00000227659 | *CLYBL-AS2* |  |
| gen1228 | 1563246_at | --- |  | ENSG00000234435 | *RP11-125P18.1* |  |
| gen1229 | 1563247_at | --- |  | ENSG00000232655 | *CTA-397C4.2* |  |
| gen1230 | 1563250_at | --- |  | ENSG00000225942 | *AC093326.2* |  |
| gen1231 | 1563254_a_at | --- |  | ENSG00000234736 | *RP11-507P23.3* |  |
| gen1232 | 1563255_at | --- |  | ENSG00000234736 | *RP11-507P23.3* |  |
| gen1233 | 1563265_at | --- |  | ENSG00000229005 | *RP5-881L22.4* |  |
| gen1234 | 1563287_at | --- |  | ENSG00000251210 | *RP11-168E17.1* |  |
| gen1235 | 1563296_at | --- |  | ENSG00000224405 | *RP11-90M5.4* |  |
| gen1236 | 1563298_at | --- |  | ENSG00000225765 | *AC068535.3* |  |
| gen1237 | 1563302_at | --- |  | ENSG00000228391 | *AC011995.3* |  |
| gen1238 | 1563316_at | --- |  | ENSG00000228853 | *RP11-175G14.1* |  |
| gen1239 | 1563341_at | --- |  | ENSG00000230612 | *AC004237.1* |  |
| gen1240 | 1563346_at | --- |  | ENSG00000240770 | *NCRNA00285* |  |
| gen1241 | 1563371_at | --- |  | ENSG00000253887 | *RP11-10A14.7* |  |
| gen1242 | 1563385_at | --- |  | ENSG00000227342 | *NCRNA00307* |  |
| gen1243 | 1563389_at | --- |  | ENSG00000223442 | *AC004041.2* |  |
| gen1244 | 1563396_x_at | --- |  | ENSG00000249349 | *CTC-384G19.1* |  |
| gen1245 | 1563397_at | --- |  | ENSG00000238201 | *AC114752.3* |  |
| gen1246 | 1563427_at | --- |  | ENSG00000232548 | *AC010744.1* |  |
| gen1247 | 1563484_at | --- |  | ENSG00000253311 | *AC011343.1* |  |
| gen1248 | 1563489_at | --- |  | ENSG00000249476 | *CTD-2587M2.1* |  |
| gen1249 | 1563539_at | --- |  | ENSG00000245883 | *AC011904.1* |  |
| gen1250 | 1563610_at | --- |  | ENSG00000254235 | *RP11-115J16.1* |  |
| gen1251 | 1563635_at | --- |  | ENSG00000235257 | *AC093415.2* |  |
| gen1252 | 1563691_at | --- |  | ENSG00000234317 | *AC108724.1* |  |
| gen1253 | 1563800_at | --- |  | ENSG00000250303 | *RP11-356J5.12* |  |
| gen1254 | 1563898_at | --- |  | ENSG00000247903 | *RP11-421F16.3* |  |
| gen1255 | 1563913_at | --- |  | ENSG00000243795 | *RP11-572M11.3* |  |
| gen1256 | 1563993_at | --- |  | ENSG00000253608 | *RP11-770E5.1* |  |
| gen1257 | 1564051_at | --- |  | ENSG00000245768 | *AC092378.1* |  |
| gen1258 | 1564083_at | --- |  | ENSG00000236714 | *AC005592.1* |  |
| gen1259 | 1564097_at | --- |  | ENSG00000241316 | *RP11-81N13.1* |  |
| gen1260 | 1564109_at | --- |  | ENSG00000249923 | *XXbac-B444P24.8* |  |
| gen1261 | 1564131_a_at | --- |  | ENSG00000226913 | *RP11-949J7.7* |  |
| gen1262 | 1564149_at | --- |  | ENSG00000237940 | *AC093642.3* |  |
| gen1263 | 1564154_at | --- |  | ENSG00000246710 | *AC009133.2* |  |
| gen1264 | 1564155_x_at | --- |  | ENSG00000246710 | *AC009133.2* |  |
| gen1265 | 1564175_at | --- |  | ENSG00000242516 | *RP11-413E6.8* |  |
| gen1266 | 1564209_at | --- |  | ENSG00000234962 | *RP11-69C17.1* |  |
| gen1267 | 1564242_at | --- |  | ENSG00000230061 | *AP001065.2* |  |
| gen1268 | 1564244_a_at | --- |  | ENSG00000224973 | *CTA-282F2.3* |  |
| gen1269 | 1564295_at | --- |  | ENSG00000231566 | *RP5-1158E12.3* |  |
| gen1270 | 1564323_at | --- |  | ENSG00000239205 | *RP11-747D18.1* |  |
| gen1271 | 1564338_at | --- |  | ENSG00000232564 | *RP4-591N18.2* |  |
| gen1272 | 1564352_at | --- |  | ENSG00000241832 | *CECR3* |  |
| gen1273 | 1564401_at | --- |  | ENSG00000235143 | *RP1-65J11.5* |  |
| gen1274 | 1564421_at | --- |  | ENSG00000253267 | *RP11-666I19.2* |  |
| gen1275 | 1564446_at | --- |  | ENSG00000224271 | *RP11-191L9.4* |  |
| gen1276 | 1564460_at | --- |  | ENSG00000235304 | *RP11-265P11.2* |  |
| gen1277 | 1564463_at | --- |  | ENSG00000246638 | *AC104759.1* |  |
| gen1278 | 1564595_at | --- |  | ENSG00000234781 | *AC068535.4* |  |
| gen1279 | 1564685_a_at | --- |  | ENSG00000225437 | *RP1-257A15.1* |  |
| gen1280 | 1564701_at | --- |  | ENSG00000235070 | *AC068138.1* |  |
| gen1281 | 1564721_at | --- |  | ENSG00000214803 | *RP11-37N22.1* |  |
| gen1282 | 1564772_at | --- |  | ENSG00000248762 | *RP11-31K23.2* |  |
| gen1283 | 1564872_at | --- |  | ENSG00000225498 | *AC002064.5* |  |
| gen1284 | 1564909_at | --- |  | ENSG00000237208 | *RP11-13E5.2* |  |
| gen1285 | 1565697_at | --- |  | ENSG00000246896 | *AC020911.1* |  |
| gen1286 | 1565729_at | --- |  | ENSG00000237058 | *RP13-436F16.1* |  |
| gen1287 | 1565730_at | --- |  | ENSG00000247484 | *AC004923.1* |  |
| gen1288 | 1565807_at | --- |  | ENSG00000236239 | *RP4-758J18.9* |  |
| gen1289 | 1565845_at | --- |  | ENSG00000249705 | *AC022413.1* |  |
| gen1290 | 1566082_at | --- |  | ENSG00000231970 | *RP11-452K12.7* |  |
| gen1291 | 1566126_at | --- |  | ENSG00000247699 | *CTB-127C13.1* |  |
| gen1292 | 1566127_at | --- |  | ENSG00000228763 | *AC010095.5* |  |
| gen1293 | 1566177_at | --- |  | ENSG00000233731 | *CLYBL-IT1* |  |
| gen1294 | 1566178_x_at | --- |  | ENSG00000233731 | *CLYBL-IT1* |  |
| gen1295 | 1566183_at | --- |  | ENSG00000248003 | *AL356356.4* |  |
| gen1296 | 1566419_at | --- |  | ENSG00000231771 | *RP11-147O5.1* |  |
| gen1297 | 1566656_a_at | --- |  | ENSG00000224356 | *RP11-151A6.4* |  |
| gen1298 | 1567183_s_at | --- |  | ENSG00000189229 | *AC069277.2* |  |
| gen1299 | 1567249_at | --- |  | ENSG00000237621 | *OR9A1P* |  |
| gen1300 | 1568635_at | --- |  | ENSG00000226903 | *RP11-75F3.1* |  |
| gen1301 | 1568636_a_at | --- |  | ENSG00000226903 | *RP11-75F3.1* |  |
| gen1302 | 1568649_at | --- |  | ENSG00000251161 | *AC025166.1* |  |
| gen1303 | 1568650_a_at | --- |  | ENSG00000251161 | *AC025166.1* |  |
| gen1304 | 1568691_at | --- |  | ENSG00000224376 | *AC017104.6* |  |
| gen1305 | 1568730_at | --- |  | ENSG00000237286 | *AC004906.3* |  |
| gen1306 | 1568749_at | --- |  | ENSG00000233656 | *RP11-716O23.1* |  |
| gen1307 | 1568754_at | --- |  | ENSG00000245927 | *AC005632.1* |  |
| gen1308 | 1568755_a_at | --- |  | ENSG00000245927 | *AC005632.1* |  |
| gen1309 | 1568780_at | --- |  | ENSG00000247397 | *AC068587.3* |  |
| gen1310 | 1568794_at | --- |  | ENSG00000231437 | *RP11-88H9.1* |  |
| gen1311 | 1568804_at | --- |  | ENSG00000225420 | *AC104134.2* |  |
| gen1312 | 1568812_at | --- |  | ENSG00000237166 | *AC007163.3* |  |
| gen1313 | 1568813_at | --- |  | ENSG00000226419 | *RP11-31F15.1* |  |
| gen1314 | 1568826_at | --- |  | ENSG00000246046 | *AL009179.1* |  |
| gen1315 | 1568844_at | --- |  | ENSG00000253281 | *RP11-716D16.1* |  |
| gen1316 | 1568848_at | --- |  | ENSG00000226308 | *RP4-813D12.3* |  |
| gen1317 | 1568851_at | --- |  | ENSG00000238078 | *RP11-295M18.2* |  |
| gen1318 | 1568852_x_at | --- |  | ENSG00000238078 | *RP11-295M18.2* |  |
| gen1319 | 1568853_at | --- |  | ENSG00000203288 | *RP11-98D18.9* |  |
| gen1320 | 1568871_at | --- |  | ENSG00000239519 | *RP11-447J13.3* |  |
| gen1321 | 1568872_at | --- |  | ENSG00000229688 | *AC004741.3* |  |
| gen1322 | 1568878_at | --- |  | ENSG00000254187 | *CTB-78F1.1* |  |
| gen1323 | 1568888_at | --- |  | ENSG00000245498 | *RP11-677M14.7* |  |
| gen1324 | 1568892_at | --- |  | ENSG00000238198 | *RP11-31F15.2* |  |
| gen1325 | 1568894_at | --- |  | ENSG00000251067 | *AC123912.2* |  |
| gen1326 | 1568899_at | --- |  | ENSG00000244773 | *AC132872.2* |  |
| gen1327 | 1568905_at | --- |  | ENSG00000224081 | *RP11-86H7.1* |  |
| gen1328 | 1568915_at | --- |  | ENSG00000230454 | *U73166.2* |  |
| gen1329 | 1568919_at | --- |  | ENSG00000225564 | *RP11-341A22.2* |  |
| gen1330 | 1568921_at | --- |  | ENSG00000253932 | *RP11-10C8.2* |  |
| gen1331 | 1568938_at | --- |  | ENSG00000224863 | *RP5-1109J22.2* |  |
| gen1332 | 1569004_at | --- |  | ENSG00000247217 | *AC008392.1* |  |
| gen1333 | 1569023_a_at | --- |  | ENSG00000234787 | *RP11-168P6.1* |  |
| gen1334 | 1569099_at | --- |  | ENSG00000253490 | *AC145110.1* |  |
| gen1335 | 1569100_a_at | --- |  | ENSG00000253490 | *AC145110.1* |  |
| gen1336 | 1569218_at | --- |  | ENSG00000254266 | *RP11-594N15.2* |  |
| gen1337 | 1569259_at | --- |  | ENSG00000226759 | *RP4-737A23.2* |  |
| gen1338 | 1569287_at | --- |  | ENSG00000234787 | *RP11-168P6.1* |  |
| gen1339 | 1569341_at | --- |  | ENSG00000203362 | *RP3-337H4.8* |  |
| gen1340 | 1569403_at | --- |  | ENSG00000203446 | *AC004988.1* |  |
| gen1341 | 1569405_at | --- |  | ENSG00000245424 | *AC011037.1* |  |
| gen1342 | 1569407_at | --- |  | ENSG00000236065 | *RP1-117O3.2* |  |
| gen1343 | 1569418_at | --- |  | ENSG00000245870 | *RP11-457P14.4* |  |
| gen1344 | 1569436_at | --- |  | ENSG00000237361 | *RP11-269C23.3* |  |
| gen1345 | 1569454_a_at | --- |  | ENSG00000249196 | *RP11-669N7.2* |  |
| gen1346 | 1569478_s_at | --- |  | ENSG00000233391 | *AC006504.1* |  |
| gen1347 | 1569486_at | --- |  | ENSG00000233288 | *RP11-760D2.5* |  |
| gen1348 | 1569525_s_at | --- |  | ENSG00000228294 /// ENSG00000229845 /// ENSG00000232775 | *AL589743.2* |  |
| gen1349 | 1569544_at | --- |  | ENSG00000240405 | *RP11-460N16.1* |  |
| gen1350 | 1569681_at | --- |  | ENSG00000226383 | *AC093375.1* |  |
| gen1351 | 1569685_at | --- |  | ENSG00000231595 | *AC005224.2* |  |
| gen1352 | 1569712_at | --- |  | ENSG00000249419 | *RP11-497K21.1* |  |
| gen1353 | 1569713_at | --- |  | ENSG00000247950 | *RP11-264J9.1* |  |
| gen1354 | 1569716_at | --- |  | ENSG00000235728 | *AC007349.5* |  |
| gen1355 | 1569727_at | --- |  | ENSG00000237013 | *AC010987.6* |  |
| gen1356 | 1569738_at | --- |  | ENSG00000232100 | *RP11-152L7.2* |  |
| gen1357 | 1569755_at | --- |  | ENSG00000230448 | *NCRNA00276* |  |
| gen1358 | 1569763_at | --- |  | ENSG00000236467 | *RP11-443A13.2* |  |
| gen1359 | 1569765_at | --- |  | ENSG00000227619 | *RP11-492E3.2* |  |
| gen1360 | 1569779_at | --- |  | ENSG00000226768 /// ENSG00000230174 /// ENSG00000231836 /// ENSG00000234858 | *XXbac-BPG181B23.4* |  |
| gen1361 | 1569780_at | --- |  | ENSG00000236795 | *AC006004.1* |  |
| gen1362 | 1569782_at | --- |  | ENSG00000228392 | *RP11-343J18.1* |  |
| gen1363 | 1569784_at | --- |  | ENSG00000250626 | *RP11-756P10.2* |  |
| gen1364 | 1569786_at | --- |  | ENSG00000240893 | *RP11-147G7.3* |  |
| gen1365 | 1569789_at | --- |  | ENSG00000239440 | *RP11-260O18.1* |  |
| gen1366 | 1569794_at | --- |  | ENSG00000236526 | *RP4-742J24.2* |  |
| gen1367 | 1569802_at | --- |  | ENSG00000225731 | *AP001627.1* |  |
| gen1368 | 1569805_at | --- |  | ENSG00000249383 | *RP11-1018N14.1* |  |
| gen1369 | 1569807_at | --- |  | ENSG00000236720 | *RP11-63B19.1* |  |
| gen1370 | 1569816_at | --- |  | ENSG00000236461 | *RP11-523L1.2* |  |
| gen1371 | 1569828_at | --- |  | ENSG00000226527 | *AP000289.6* |  |
| gen1372 | 1569831_at | --- |  | ENSG00000253553 | *RP11-586K2.1* |  |
| gen1373 | 1569833_at | --- |  | ENSG00000245479 | *AC021422.1* |  |
| gen1374 | 1569838_at | --- |  | ENSG00000246394 | *RP11-386I8.6* |  |
| gen1375 | 1569840_at | --- |  | ENSG00000253165 | *CTD-2309H9.3* |  |
| gen1376 | 1569841_x_at | --- |  | ENSG00000253165 | *CTD-2309H9.3* |  |
| gen1377 | 1569846_at | --- |  | ENSG00000248975 | *CTD-2251F13.1* |  |
| gen1378 | 1569849_at | --- |  | ENSG00000231883 | *RP1-297M16.2* |  |
| gen1379 | 1569858_at | --- |  | ENSG00000227809 | *RP11-171A24.2* |  |
| gen1380 | 1569859_at | --- |  | ENSG00000227848 | *SUCLA2-AS1* |  |
| gen1381 | 1569885_at | --- |  | ENSG00000242759 | *RP11-280F2.2* |  |
| gen1382 | 1569911_at | --- |  | ENSG00000254275 | *RP11-234A3.1* |  |
| gen1383 | 1569912_at | --- |  | ENSG00000235023 | *AP001626.2* |  |
| gen1384 | 1569954_at | --- |  | ENSG00000233968 | *RP11-354E11.2* |  |
| gen1385 | 1569958_at | --- |  | ENSG00000234264 | *RP4-694A7.4* |  |
| gen1386 | 1569961_at | --- |  | ENSG00000250631 | *AL353896.1* |  |
| gen1387 | 1569963_at | --- |  | ENSG00000226706 | *RP11-426A6.5* |  |
| gen1388 | 1570044_at | --- |  | ENSG00000227038 | *AC005077.12* |  |
| gen1389 | 1570064_at | --- |  | ENSG00000250596 | *RP11-440I14.2* |  |
| gen1390 | 1570080_at | --- |  | ENSG00000235527 | *RP5-1073O3.7* |  |
| gen1391 | 1570082_x_at | --- |  | ENSG00000235527 | *RP5-1073O3.7* |  |
| gen1392 | 1570106_at | --- |  | ENSG00000225300 | *RP11-439E19.1* |  |
| gen1393 | 1570116_at | --- |  | ENSG00000230773 | *AC079807.4* |  |
| gen1394 | 1570120_at | --- |  | ENSG00000228933 | *RP11-268G12.1* |  |
| gen1395 | 1570155_at | --- |  | ENSG00000254300 | *RP11-706J10.1* |  |
| gen1396 | 1570163_at | --- |  | ENSG00000248730 | *CTD-2022B6.1* |  |
| gen1397 | 1570206_at | --- |  | ENSG00000233569 | *RP11-500B12.1* |  |
| gen1398 | 1570224_at | --- |  | ENSG00000224017 | *AC005022.1* |  |
| gen1399 | 1570226_at | --- |  | ENSG00000250365 | *CTD-2213F21.2* |  |
| gen1400 | 1570230_at | --- |  | ENSG00000231172 | *AC007099.1* |  |
| gen1401 | 1570262_at | --- |  | ENSG00000248505 | *RP11-319E12.1* |  |
| gen1402 | 1570268_at | --- |  | ENSG00000246541 | *RP11-363G15.2* |  |
| gen1403 | 1570270_at | --- |  | ENSG00000236373 | *RP11-15K3.1* |  |
| gen1404 | 1570273_at | --- |  | ENSG00000234653 | *AC079117.1* |  |
| gen1405 | 1570285_at | --- |  | ENSG00000238005 | *RP11-443B7.1* |  |
| gen1406 | 1570291_at | --- |  | ENSG00000223392 | *CLDN10-AS1* |  |
| gen1407 | 1570292_at | --- |  | ENSG00000244215 | *RP11-88I21.2* |  |
| gen1408 | 1570301_at | --- |  | ENSG00000254288 | *RP11-6I2.3* |  |
| gen1409 | 1570375_at | --- |  | ENSG00000233038 | *AC011899.9* |  |
| gen1410 | 1570405_at | --- |  | ENSG00000245719 | *AC009292.1* |  |
| gen1411 | 1570423_at | --- |  | ENSG00000237975 | *RP1-14N1.2* |  |
| gen1412 | 1570469_at | --- |  | ENSG00000234323 | *RP11-308N19.1* |  |
| gen1413 | 1570568_at | --- |  | ENSG00000238204 | *AC012354.4* |  |
| gen1414 | 1570587_at | --- |  | ENSG00000225442 | *AC079111.1* |  |
| gen1415 | 207489_at | --- |  | ENSG00000244378 | *RPS2P45* |  |
| gen1416 | 207750_at | --- |  | ENSG00000242948 | *EPS15P1* |  |
| gen1417 | 209697_at | --- |  | ENSG00000251034 | *RP11-582J16.4* |  |
| gen1418 | 211456_x_at | --- |  | ENSG00000244020 | *AL359259.1* |  |
| gen1419 | 213525_at | --- |  | ENSG00000239791 | *AC002310.7* |  |
| gen1420 | 213685_at | --- |  | ENSG00000236810 | *RP5-886K2.3* |  |
| gen1421 | 213810_s_at | --- |  | ENSG00000224345 | *AKIRIN2-AS1* |  |
| gen1422 | 213972_at | --- |  | ENSG00000247993 | *RP11-79P5.2* |  |
| gen1423 | 214202_at | --- |  | ENSG00000248175 | *CTC-428G20.3* |  |
| gen1424 | 214345_at | --- |  | ENSG00000250320 | *CTD-2269F5.1* |  |
| gen1425 | 214857_at | --- |  | ENSG00000245773 | *AL121928.1* |  |
| gen1426 | 214867_at | --- |  | ENSG00000236370 | *RP11-574K11.16* |  |
| gen1427 | 215118_s_at | --- |  | ENSG00000253701 | *AL928768.3* |  |
| gen1428 | 215185_at | --- |  | ENSG00000204054 | *RP11-492E3.1* |  |
| gen1429 | 215298_at | --- |  | ENSG00000251632 | *RP11-714L20.1* |  |
| gen1430 | 215318_at | --- |  | ENSG00000230905 | *N4BP2L2-IT1* |  |
| gen1431 | 215327_at | --- |  | ENSG00000213994 | *RP11-414H17.5* |  |
| gen1432 | 215565_at | --- |  | ENSG00000224220 | *AC104699.1* |  |
| gen1433 | 215627_at | --- |  | ENSG00000234350 | *AC007405.4* |  |
| gen1434 | 215755_at | --- |  | ENSG00000222448 | *7SK* |  |
| gen1435 | 215861_at | --- |  | ENSG00000197670 | *RP4-724E16.2* |  |
| gen1436 | 215876_at | --- |  | ENSG00000226334 | *RP11-217B7.2* |  |
| gen1437 | 215929_at | --- |  | ENSG00000235824 | *RP11-478H13.2* |  |
| gen1438 | 215944_at | --- |  | ENSG00000236268 | *RP11-170N11.1* |  |
| gen1439 | 215975_x_at | --- |  | ENSG00000243055 | *RP11-242C19.3* |  |
| gen1440 | 216051_x_at | --- |  | ENSG00000228508 | *RP11-183E9.3* |  |
| gen1441 | 216110_x_at | --- |  | ENSG00000228389 | *AC068039.4* |  |
| gen1442 | 216319_at | --- |  | ENSG00000236673 | *RP11-69I8.2* |  |
| gen1443 | 216376_x_at | --- |  | ENSG00000227822 /// ENSG00000241859 | *AC006370.2* |  |
| gen1444 | 216445_at | --- |  | ENSG00000227612 | *RP5-1177E19.2* |  |
| gen1445 | 216459_x_at | --- |  | ENSG00000223405 /// ENSG00000224360 /// ENSG00000227422 /// ENSG00000231444 /// ENSG00000236492 /// ENSG00000237775 | *TIGD1L* |  |
| gen1446 | 216543_at | --- |  | ENSG00000231216 | *GS1-600G8.3* |  |
| gen1447 | 216566_at | --- |  | ENSG00000240269 | *D87024.2* |  |
| gen1448 | 216597_at | --- |  | ENSG00000242115 | *U66059.57* |  |
| gen1449 | 216664_at | --- |  | ENSG00000235059 /// ENSG00000236951 | *AC008175.1* |  |
| gen1450 | 216719_s_at | --- |  | ENSG00000249604 | *RP11-286E11.2* |  |
| gen1451 | 216849_at | --- |  | ENSG00000235725 | *AC007389.3* |  |
| gen1452 | 216935_at | --- |  | ENSG00000176075 | *NCRNA00302* |  |
| gen1453 | 217054_at | --- |  | ENSG00000225946 | *RP11-395B7.2* |  |
| gen1454 | 217193_x_at | --- |  | ENSG00000240269 | *D87024.2* |  |
| gen1455 | 217194_at | --- |  | ENSG00000226585 | *RP4-765C7.1* |  |
| gen1456 | 217328_at | --- |  | ENSG00000246032 | *AC104597.1* |  |
| gen1457 | 217406_at | --- |  | ENSG00000241954 | *RP1-149A16.17* |  |
| gen1458 | 217443_at | --- |  | ENSG00000246777 | *AC044802.1* |  |
| gen1459 | 217444_at | --- |  | ENSG00000236772 | *RP5-1184F4.5* |  |
| gen1460 | 217449_at | --- |  | ENSG00000248103 | *CTC-338M12.9* |  |
| gen1461 | 220687_at | --- |  | ENSG00000242687 | *AC004893.11* |  |
| gen1462 | 220846_s_at | --- |  | ENSG00000182873 | *RP11-181G12.2* |  |
| gen1463 | 220855_at | --- |  | ENSG00000229507 | *AC091271.2* |  |
| gen1464 | 220900_at | --- |  | ENSG00000251023 | *RP11-549J18.1* |  |
| gen1465 | 221183_at | --- |  | ENSG00000249717 | *RP11-44F21.3* |  |
| gen1466 | 221929_at | --- |  | ENSG00000253722 | *RP11-10N23.4* |  |
| gen1467 | 222051_s_at | --- |  | ENSG00000254208 | *RP11-219B4.3* |  |
| gen1468 | 222060_at | --- |  | ENSG00000229320 | *KRT8P12* |  |
| gen1469 | 222087_at | --- |  | ENSG00000249859 | *PVT1* |  |
| gen1470 | 222376_at | --- |  | ENSG00000248161 | *RP11-499E18.1* |  |
| gen1471 | 224005_at | --- |  | ENSG00000230850 | *BX571672.1* |  |
| gen1472 | 224006_at | --- |  | ENSG00000251138 | *RP11-81H3.2* |  |
| gen1473 | 224644_at | --- |  | ENSG00000225733 | *AC090937.2* |  |
| gen1474 | 225860_at | --- |  | ENSG00000126005 | *MT1P3* |  |
| gen1475 | 226341_at | --- |  | ENSG00000253738 | *GS1-251I9.4* |  |
| gen1476 | 227389_x_at | --- |  | ENSG00000228830 | *RP4-781K5.2* |  |
| gen1477 | 227431_at | --- |  | ENSG00000214293 | *AC090421.1* |  |
| gen1478 | 227437_at | --- |  | ENSG00000248008 | *RP11-18C24.6* |  |
| gen1479 | 227502_at | --- |  | ENSG00000244701 | *RP5-894A10.2* |  |
| gen1480 | 227591_at | --- |  | ENSG00000224660 | *AC087590.3* |  |
| gen1481 | 227724_at | --- |  | ENSG00000223482 /// ENSG00000226381 | *RP11-322M19.1* |  |
| gen1482 | 228102_at | --- |  | ENSG00000225216 | *AC007362.3* |  |
| gen1483 | 228103_s_at | --- |  | ENSG00000225216 | *AC007362.3* |  |
| gen1484 | 228147_at | --- |  | ENSG00000214765 | *SEPT7P2* |  |
| gen1485 | 228209_at | --- |  | ENSG00000230124 | *RP5-1180C10.2* |  |
| gen1486 | 228259_s_at | --- |  | ENSG00000224032 | *NCRNA00219* |  |
| gen1487 | 228275_at | --- |  | ENSG00000240024 | *RP11-393B14.1* |  |
| gen1488 | 228430_at | --- |  | ENSG00000225439 | *AC073263.4* |  |
| gen1489 | 228440_at | --- |  | ENSG00000251603 | *RP11-164P12.4* |  |
| gen1490 | 228441_s_at | --- |  | ENSG00000251603 | *RP11-164P12.4* |  |
| gen1491 | 228526_at | --- |  | ENSG00000230139 | *RP11-498J9.4* |  |
| gen1492 | 228549_at | --- |  | ENSG00000242861 | *RP11-285F7.2* |  |
| gen1493 | 228609_at | --- |  | ENSG00000227518 | *MIR1302-10* |  |
| gen1494 | 228612_at | --- |  | ENSG00000246067 | *RP11-113K21.5* |  |
| gen1495 | 228632_at | --- |  | ENSG00000223403 | *AL132709.7* |  |
| gen1496 | 228642_at | --- |  | ENSG00000233429 | *HOTAIRM1* |  |
| gen1497 | 228643_at | --- |  | ENSG00000246067 | *RP11-113K21.5* |  |
| gen1498 | 228661_s_at | --- |  | ENSG00000233184 | *RP11-421L21.3* |  |
| gen1499 | 228672_at | --- |  | ENSG00000235351 | *AC114730.11* |  |
| gen1500 | 228673_s_at | --- |  | ENSG00000224875 | *AC083949.1* |  |
| gen1501 | 228895_s_at | --- |  | ENSG00000229915 | *AC016999.2* |  |
| gen1502 | 229006_at | --- |  | ENSG00000251432 | *RP11-420A23.1* |  |
| gen1503 | 229040_at | --- |  | ENSG00000227039 | *AL844908.5* |  |
| gen1504 | 229041_s_at | --- |  | ENSG00000227039 | *AL844908.5* |  |
| gen1505 | 229130_at | --- |  | ENSG00000251615 | *RP11-774O3.3* |  |
| gen1506 | 229204_at | --- |  | ENSG00000203394 | *RP5-930J4.4* |  |
| gen1507 | 229363_at | --- |  | ENSG00000246898 | *AC132186.1* |  |
| gen1508 | 229388_at | --- |  | ENSG00000227540 | *RP11-152N13.5* |  |
| gen1509 | 229409_s_at | --- |  | ENSG00000238280 | *RP11-436D10.3* |  |
| gen1510 | 229420_at | --- |  | ENSG00000240567 | *RP11-3P17.4* |  |
| gen1511 | 229505_at | --- |  | ENSG00000224636 | *AC131971.3* |  |
| gen1512 | 229543_at | --- |  | ENSG00000244158 | *RP1-93H18.6* |  |
| gen1513 | 229592_at | --- |  | ENSG00000245388 | *AC009171.1* |  |
| gen1514 | 229635_at | --- |  | ENSG00000251442 | *RP11-792D21.2* |  |
| gen1515 | 229640_x_at | --- |  | ENSG00000250943 | *AL157931.2* |  |
| gen1516 | 229643_at | --- |  | ENSG00000226963 /// ENSG00000232788 | *AC078883.4* |  |
| gen1517 | 229716_at | --- |  | ENSG00000225986 | *RP3-340N1.5* |  |
| gen1518 | 229874_x_at | --- |  | ENSG00000238142 | *RP11-108M9.4* |  |
| gen1519 | 229896_at | --- |  | ENSG00000232729 | *AC083884.8* |  |
| gen1520 | 230073_at | --- |  | ENSG00000223745 | *RP4-717I23.3* |  |
| gen1521 | 230171_at | --- |  | ENSG00000246560 | *RP11-10L12.4* |  |
| gen1522 | 230184_at | --- |  | ENSG00000233639 | *AC018730.1* |  |
| gen1523 | 230301_at | --- |  | ENSG00000234286 | *AC006026.13* |  |
| gen1524 | 230353_at | --- |  | ENSG00000232607 | *MIR497HG* |  |
| gen1525 | 230410_at | --- |  | ENSG00000225216 | *AC007362.3* |  |
| gen1526 | 230423_at | --- |  | ENSG00000230798 | *RP4-792G4.2* |  |
| gen1527 | 230449_x_at | --- |  | ENSG00000253948 | *RP11-410L14.2* |  |
| gen1528 | 230450_at | --- |  | ENSG00000228878 | *AC007551.3* |  |
| gen1529 | 230451_at | --- |  | ENSG00000236088 | *AC015842.1* |  |
| gen1530 | 230460_at | --- |  | ENSG00000239523 | *RP11-202D20.1* |  |
| gen1531 | 230653_at | --- |  | ENSG00000250763 | *RP11-974F13.6* |  |
| gen1532 | 230776_at | --- |  | ENSG00000247180 | *AC018665.1* |  |
| gen1533 | 230817_at | --- |  | ENSG00000254010 | *RP11-103H7.5* |  |
| gen1534 | 230905_at | --- |  | ENSG00000232581 | *AC079742.4* |  |
| gen1535 | 230953_at | --- |  | ENSG00000178836 | *AC114812.5* |  |
| gen1536 | 231061_at | --- |  | ENSG00000249532 | *RP11-148B6.1* |  |
| gen1537 | 231089_at | --- |  | ENSG00000230387 | *RP4-737E23.2* |  |
| gen1538 | 231092_s_at | --- |  | ENSG00000247033 | *AC099508.1* |  |
| gen1539 | 231105_at | --- |  | ENSG00000235358 | *RP11-399E6.1* |  |
| gen1540 | 231237_x_at | --- |  | ENSG00000254208 | *RP11-219B4.3* |  |
| gen1541 | 231249_at | --- |  | ENSG00000229348 | *RP11-506B15.6* |  |
| gen1542 | 231250_at | --- |  | ENSG00000233283 | *RP11-357H14.20* |  |
| gen1543 | 231307_at | --- |  | ENSG00000232765 /// ENSG00000251617 | *RP11-382F24.2* |  |
| gen1544 | 231344_at | --- |  | ENSG00000227907 | *RP11-102C16.3* |  |
| gen1545 | 231364_at | --- |  | ENSG00000237928 | *RP4-668G5.1* |  |
| gen1546 | 231365_at | --- |  | ENSG00000253187 /// ENSG00000253289 | *RP1-170O19.6* |  |
| gen1547 | 231378_at | --- |  | ENSG00000253408 | *RP11-231D20.2* |  |
| gen1548 | 231410_at | --- |  | ENSG00000250377 | *CTC-467M3.3* |  |
| gen1549 | 231412_at | --- |  | ENSG00000251136 | *RP11-37B2.1* |  |
| gen1550 | 231458_at | --- |  | ENSG00000226995 | *RP5-828H9.3* |  |
| gen1551 | 231485_at | --- |  | ENSG00000234855 | *RP11-453E2.2* |  |
| gen1552 | 231521_at | --- |  | ENSG00000067601 | *PMS2P4* |  |
| gen1553 | 231557_at | --- |  | ENSG00000250604 | *RP11-597D13.8* |  |
| gen1554 | 231563_at | --- |  | ENSG00000232265 | *XXyac-YX155B6.5* |  |
| gen1555 | 231564_at | --- |  | ENSG00000233728 | *RP11-109P14.9* |  |
| gen1556 | 231613_at | --- |  | ENSG00000225782 | *RP11-322F10.2* |  |
| gen1557 | 231694_at | --- |  | ENSG00000235910 | *AP006216.12* |  |
| gen1558 | 231925_at | --- |  | ENSG00000241732 | *RP11-38P22.2* |  |
| gen1559 | 231942_at | --- |  | ENSG00000228794 /// ENSG00000249868 | *RP11-206L10.11* |  |
| gen1560 | 232022_at | --- |  | ENSG00000235381 | *RP11-477D19.2* |  |
| gen1561 | 232047_at | --- |  | ENSG00000242086 | *AC069513.3* |  |
| gen1562 | 232096_x_at | --- |  | ENSG00000242094 | *RP11-154H23.1* |  |
| gen1563 | 232107_at | --- |  | ENSG00000235477 | *RP11-122G18.5* |  |
| gen1564 | 232111_at | --- |  | ENSG00000250366 | *AL133167.1* |  |
| gen1565 | 232191_at | --- |  | ENSG00000233056 | *ERVH48-1* |  |
| gen1566 | 232241_at | --- |  | ENSG00000235703 | *RP13-507I23.1* |  |
| gen1567 | 232242_at | --- |  | ENSG00000241472 | *RP11-204J18.3* |  |
| gen1568 | 232300_at | --- |  | ENSG00000233165 | *RP11-96C23.8* |  |
| gen1569 | 232320_at | --- |  | ENSG00000232586 | *RP11-46A10.4* |  |
| gen1570 | 232340_at | --- |  | ENSG00000244625 | *CTA-211A9.5* |  |
| gen1571 | 232504_at | --- |  | ENSG00000253522 | *CTC-231O11.1* |  |
| gen1572 | 232512_at | --- |  | ENSG00000236283 | *AC013463.2* |  |
| gen1573 | 232522_at | --- |  | ENSG00000237996 | *RP11-258A12.3* |  |
| gen1574 | 232589_at | --- |  | ENSG00000233184 | *RP11-421L21.3* |  |
| gen1575 | 232790_at | --- |  | ENSG00000234171 | *AC108488.3* |  |
| gen1576 | 232804_at | --- |  | ENSG00000234380 | *AP000330.8* |  |
| gen1577 | 232827_at | --- |  | ENSG00000246981 | *AL139044.1* |  |
| gen1578 | 232828_at | --- |  | ENSG00000233009 | *RP11-430M15.1* |  |
| gen1579 | 232839_at | --- |  | ENSG00000224418 | *STK24-AS1* |  |
| gen1580 | 232880_at | --- |  | ENSG00000247484 | *AC004923.1* |  |
| gen1581 | 232965_at | --- |  | ENSG00000249530 | *AC007773.1* |  |
| gen1582 | 233016_at | --- |  | ENSG00000229191 | *RP11-168O16.1* |  |
| gen1583 | 233021_at | --- |  | ENSG00000227354 | *RBM26-AS1* |  |
| gen1584 | 233048_at | --- |  | ENSG00000229227 | *RP11-38L15.2* |  |
| gen1585 | 233130_at | --- |  | ENSG00000224822 | *AC112217.2* |  |
| gen1586 | 233135_at | --- |  | ENSG00000233123 | *RP5-1059M17.1* |  |
| gen1587 | 233142_at | --- |  | ENSG00000246181 | *AC022409.1* |  |
| gen1588 | 233149_at | --- |  | ENSG00000243024 | *AC012158.1* |  |
| gen1589 | 233162_at | --- |  | ENSG00000253967 | *RP11-333A23.4* |  |
| gen1590 | 233170_at | --- |  | ENSG00000226846 | *RP11-77P3.1* |  |
| gen1591 | 233174_at | --- |  | ENSG00000246089 | *RP11-115C21.2* |  |
| gen1592 | 233176_at | --- |  | ENSG00000240859 | *AC093627.10* |  |
| gen1593 | 233224_at | --- |  | ENSG00000224798 | *KPNA3-IT1* |  |
| gen1594 | 233237_at | --- |  | ENSG00000254226 | *CTB-12O2.1* |  |
| gen1595 | 233238_s_at | --- |  | ENSG00000254226 | *CTB-12O2.1* |  |
| gen1596 | 233410_at | --- |  | ENSG00000232973 | *CYP1B1-AS1* |  |
| gen1597 | 233475_at | --- |  | ENSG00000250328 | *CTC-210G5.1* |  |
| gen1598 | 233513_at | --- |  | ENSG00000245482 | *RP11-847H18.2* |  |
| gen1599 | 233522_at | --- |  | ENSG00000248309 | *CTC-454M9.1* |  |
| gen1600 | 233529_at | --- |  | ENSG00000231428 | *RP11-472K17.1* |  |
| gen1601 | 233530_at | --- |  | ENSG00000229728 | *RP11-314N13.3* |  |
| gen1602 | 233567_at | --- |  | ENSG00000231871 | *RP11-90L20.3* |  |
| gen1603 | 233583_at | --- |  | ENSG00000231078 | *RP11-560A15.4* |  |
| gen1604 | 233590_at | --- |  | ENSG00000224184 | *AC096559.1* |  |
| gen1605 | 233593_at | --- |  | ENSG00000251455 | *RP11-164P12.3* |  |
| gen1606 | 233594_at | --- |  | ENSG00000229688 | *AC004741.3* |  |
| gen1607 | 233747_at | --- |  | ENSG00000235154 | *CTA-280A3__B.2* |  |
| gen1608 | 233749_at | --- |  | ENSG00000231669 | *RP11-368D24__A.1* |  |
| gen1609 | 233755_at | --- |  | ENSG00000253706 | *RP11-758M4.4* |  |
| gen1610 | 233770_at | --- |  | ENSG00000182404 | *RP1-86D1.3* |  |
| gen1611 | 233784_at | --- |  | ENSG00000226097 | *AC099342.1* |  |
| gen1612 | 233928_at | --- |  | ENSG00000241158 | *RP11-14D22.5* |  |
| gen1613 | 233934_at | --- |  | ENSG00000234352 | *AC009264.1* |  |
| gen1614 | 233962_at | --- |  | ENSG00000235214 | *NCRNA00154* |  |
| gen1615 | 233981_at | --- |  | ENSG00000243754 | *RP11-125K10.4* |  |
| gen1616 | 234020_x_at | --- |  | ENSG00000229839 | *AC018462.2* |  |
| gen1617 | 234053_at | --- |  | ENSG00000224190 | *RP11-442O18.2* |  |
| gen1618 | 234138_at | --- |  | ENSG00000231229 | *RP6-239D12.1* |  |
| gen1619 | 234139_s_at | --- |  | ENSG00000231229 | *RP6-239D12.1* |  |
| gen1620 | 234141_s_at | --- |  | ENSG00000246130 | *RP11-875O11.2* |  |
| gen1621 | 234143_at | --- |  | ENSG00000251405 | *CTB-109A12.1* |  |
| gen1622 | 234171_at | --- |  | ENSG00000251576 | *RP11-536I6.1* |  |
| gen1623 | 234190_at | --- |  | ENSG00000248623 | *AP001630.1* |  |
| gen1624 | 234208_at | --- |  | ENSG00000253519 | *AC106801.1* |  |
| gen1625 | 234214_at | --- |  | ENSG00000244951 | *AL365502.1* |  |
| gen1626 | 234219_at | --- |  | ENSG00000234155 | *RP11-30P6.6* |  |
| gen1627 | 234234_at | --- |  | ENSG00000237686 | *RP5-1120P11.1* |  |
| gen1628 | 234250_at | --- |  | ENSG00000228496 | *AC106875.1* |  |
| gen1629 | 234267_at | --- |  | ENSG00000249346 | *RP3-468B3.3* |  |
| gen1630 | 234288_at | --- |  | ENSG00000254119 | *RP11-705O24.1* |  |
| gen1631 | 234293_x_at | --- |  | ENSG00000177788 /// ENSG00000240524 | *RP5-1061H20.4* |  |
| gen1632 | 234301_s_at | --- |  | ENSG00000235381 | *RP11-477D19.2* |  |
| gen1633 | 234345_at | --- |  | ENSG00000231881 | *RP5-1120P11.3* |  |
| gen1634 | 234364_at | --- |  | ENSG00000240269 | *D87024.2* |  |
| gen1635 | 234373_x_at | --- |  | ENSG00000227468 | *AL928742.12* |  |
| gen1636 | 234400_at | --- |  | ENSG00000230839 | *RP5-968J1.1* |  |
| gen1637 | 234443_at | --- |  | ENSG00000250433 | *RP11-13L2.2* |  |
| gen1638 | 234707_x_at | --- |  | ENSG00000240269 | *D87024.2* |  |
| gen1639 | 234763_at | --- |  | ENSG00000242815 | *RP4-665N4.4* |  |
| gen1640 | 234773_x_at | --- |  | ENSG00000248339 | *RP11-717H13.1* |  |
| gen1641 | 234787_at | --- |  | ENSG00000245538 | *AC126327.1* |  |
| gen1642 | 234825_at | --- |  | ENSG00000247735 | *AC120114.3* |  |
| gen1643 | 234826_at | --- |  | ENSG00000230552 | *AC092162.1* |  |
| gen1644 | 234877_x_at | --- |  | ENSG00000240269 | *D87024.2* |  |
| gen1645 | 234890_at | --- |  | ENSG00000217801 | *RP11-465B22.3* |  |
| gen1646 | 234940_s_at | --- |  | ENSG00000243176 | *RP11-550I24.2* |  |
| gen1647 | 235152_at | --- |  | ENSG00000245571 | *AP001258.4* |  |
| gen1648 | 235227_at | --- |  | ENSG00000225135 | *RP11-361F15.2* |  |
| gen1649 | 235373_at | --- |  | ENSG00000247498 | *RP11-392P7.8* |  |
| gen1650 | 235386_at | --- |  | ENSG00000251615 | *RP11-774O3.3* |  |
| gen1651 | 235445_at | --- |  | ENSG00000236666 /// ENSG00000250217 | *LA16c-3G11.5* |  |
| gen1652 | 235464_at | --- |  | ENSG00000230551 | *CTB-89H12.4* |  |
| gen1653 | 235466_s_at | --- |  | ENSG00000228106 | *RP11-452F19.3* |  |
| gen1654 | 235534_at | --- |  | ENSG00000224818 | *RP11-134G8.8* |  |
| gen1655 | 235617_x_at | --- |  | ENSG00000240045 | *RP11-451G4.2* |  |
| gen1656 | 235628_x_at | --- |  | ENSG00000245937 | *CTC-228N24.3* |  |
| gen1657 | 235696_at | --- |  | ENSG00000247134 | *RP11-11N9.4* |  |
| gen1658 | 235759_at | --- |  | ENSG00000253250 | *RP11-122A3.2* |  |
| gen1659 | 235824_at | --- |  | ENSG00000233236 | *AP001171.1* |  |
| gen1660 | 235921_at | --- |  | ENSG00000223784 | *RP11-554I8.2* |  |
| gen1661 | 235934_at | --- |  | ENSG00000245602 | *AC073548.1* |  |
| gen1662 | 235945_at | --- |  | ENSG00000049319 | *SRD5A2* |  |
| gen1663 | 236036_at | --- |  | ENSG00000227811 | *RP4-773A18.4* |  |
| gen1664 | 236093_at | --- |  | ENSG00000246051 | *AC020915.1* |  |
| gen1665 | 236148_at | --- |  | ENSG00000236914 | *AC087473.1* |  |
| gen1666 | 236176_at | --- |  | ENSG00000232453 | *RP4-794H19.1* |  |
| gen1667 | 236213_at | --- |  | ENSG00000222043 | *AC079305.10* |  |
| gen1668 | 236271_at | --- |  | ENSG00000254027 | *RP11-363E6.3* |  |
| gen1669 | 236282_at | --- |  | ENSG00000224046 | *AC005076.5* |  |
| gen1670 | 236336_at | --- |  | ENSG00000225439 | *AC073263.4* |  |
| gen1671 | 236398_s_at | --- |  | ENSG00000238005 | *RP11-443B7.1* |  |
| gen1672 | 236399_at | --- |  | ENSG00000238005 | *RP11-443B7.1* |  |
| gen1673 | 236444_x_at | --- |  | ENSG00000249082 | *CTC-276P9.1* |  |
| gen1674 | 236451_at | --- |  | ENSG00000227403 | *AC009299.3* |  |
| gen1675 | 236480_at | --- |  | ENSG00000247095 | *MIR210HG* |  |
| gen1676 | 236575_at | --- |  | ENSG00000243069 | *RP11-217E22.2* |  |
| gen1677 | 236598_at | --- |  | ENSG00000227403 | *AC009299.3* |  |
| gen1678 | 236658_at | --- |  | ENSG00000231654 | *RP11-514O12.2* |  |
| gen1679 | 236695_at | --- |  | ENSG00000227477 | *RP5-1069P2.4* |  |
| gen1680 | 236788_at | --- |  | ENSG00000231964 | *RP11-67C2.2* |  |
| gen1681 | 236798_at | --- |  | ENSG00000240024 | *RP11-393B14.1* |  |
| gen1682 | 236870_at | --- |  | ENSG00000224792 | *RP11-314A5.3* |  |
| gen1683 | 236990_at | --- |  | ENSG00000234028 | *AC062029.1* |  |
| gen1684 | 237065_s_at | --- |  | ENSG00000224699 | *RP11-225L12.2* |  |
| gen1685 | 237115_at | --- |  | ENSG00000232874 | *RP11-135A1.2* |  |
| gen1686 | 237154_at | --- |  | ENSG00000227591 | *RP1-28O10.1* |  |
| gen1687 | 237173_at | --- |  | ENSG00000223779 /// ENSG00000232151 /// ENSG00000235398 /// ENSG00000236943 | *RP11-403I13.4* |  |
| gen1688 | 237192_at | --- |  | ENSG00000243004 | *AC005062.2* |  |
| gen1689 | 237193_s_at | --- |  | ENSG00000243004 | *AC005062.2* |  |
| gen1690 | 237203_at | --- |  | ENSG00000229191 | *RP11-168O16.1* |  |
| gen1691 | 237224_at | --- |  | ENSG00000224805 | *RP1-18D14.4* |  |
| gen1692 | 237253_at | --- |  | ENSG00000239877 | *RP4-635B5.1* |  |
| gen1693 | 237343_at | --- |  | ENSG00000237401 | *AC107070.1* |  |
| gen1694 | 237351_at | --- |  | ENSG00000232079 | *AL035610.1* |  |
| gen1695 | 237421_at | --- |  | ENSG00000248636 | *RP11-768F21.1* |  |
| gen1696 | 237425_at | --- |  | ENSG00000226387 | *RP11-483B5.3* |  |
| gen1697 | 237501_at | --- |  | ENSG00000234690 | *AC073283.4* |  |
| gen1698 | 237529_at | --- |  | ENSG00000232170 | *GS1-756B1.2* |  |
| gen1699 | 237543_at | --- |  | ENSG00000246943 | *AC013722.2* |  |
| gen1700 | 237563_s_at | --- |  | ENSG00000233461 | *RP11-295G20.2* |  |
| gen1701 | 237564_at | --- |  | ENSG00000233461 | *RP11-295G20.2* |  |
| gen1702 | 237595_at | --- |  | ENSG00000224702 | *RP11-38C18.2* |  |
| gen1703 | 237675_at | --- |  | ENSG00000237457 | *RP11-547I7.2* |  |
| gen1704 | 237682_at | --- |  | ENSG00000249001 | *RP11-742B18.1* |  |
| gen1705 | 237696_at | --- |  | ENSG00000248184 | *RP11-231C18.1* |  |
| gen1706 | 237729_at | --- |  | ENSG00000246145 | *RP11-346I3.4* |  |
| gen1707 | 237734_s_at | --- |  | ENSG00000249833 | *RP11-71E19.5* |  |
| gen1708 | 237735_at | --- |  | ENSG00000249833 | *RP11-71E19.5* |  |
| gen1709 | 237738_at | --- |  | ENSG00000253551 | *RP11-26M5.2* |  |
| gen1710 | 237758_at | --- |  | ENSG00000251249 | *RP11-73G16.1* |  |
| gen1711 | 237760_at | --- |  | ENSG00000227290 | *RP4-544H6.2* |  |
| gen1712 | 237771_s_at | --- |  | ENSG00000251676 | *RP11-614F17.2* |  |
| gen1713 | 237772_at | --- |  | ENSG00000225187 | *AC073283.7* |  |
| gen1714 | 237787_at | --- |  | ENSG00000236866 | *AL157902.3* |  |
| gen1715 | 237789_at | --- |  | ENSG00000233203 | *RP11-67L3.4* |  |
| gen1716 | 237838_at | --- |  | ENSG00000228735 | *GS1-18A18.2* |  |
| gen1717 | 237845_at | --- |  | ENSG00000231616 | *RP11-575L7.4* |  |
| gen1718 | 237861_at | --- |  | ENSG00000247324 | *AC010547.1* |  |
| gen1719 | 237871_x_at | --- |  | ENSG00000235009 | *AC093822.1* |  |
| gen1720 | 237880_at | --- |  | ENSG00000224184 | *AC096559.1* |  |
| gen1721 | 237964_at | --- |  | ENSG00000224456 | *AP003774.7* |  |
| gen1722 | 238103_at | --- |  | ENSG00000248771 | *RP11-294O2.2* |  |
| gen1723 | 238126_at | --- |  | ENSG00000214293 | *AC090421.1* |  |
| gen1724 | 238180_at | --- |  | ENSG00000227053 | *RP11-395B7.4* |  |
| gen1725 | 238194_at | --- |  | ENSG00000204466 | *DGKK* |  |
| gen1726 | 238225_at | --- |  | ENSG00000253522 | *CTC-231O11.1* |  |
| gen1727 | 238251_at | --- |  | ENSG00000229565 | *AC108056.1* |  |
| gen1728 | 238278_at | --- |  | ENSG00000231983 | *RP11-398O19.3* |  |
| gen1729 | 238290_at | --- |  | ENSG00000233246 | *RP11-415J8.5* |  |
| gen1730 | 238308_at | --- |  | ENSG00000237605 | *RP11-343H5.6* |  |
| gen1731 | 238522_at | --- |  | ENSG00000221817 | *RP11-137L10.6* |  |
| gen1732 | 238766_at | --- |  | ENSG00000237436 | *RP11-312B8.1* |  |
| gen1733 | 238804_at | --- |  | ENSG00000236833 | *AC024560.2* |  |
| gen1734 | 238854_at | --- |  | ENSG00000248008 | *RP11-18C24.6* |  |
| gen1735 | 238944_at | --- |  | ENSG00000247377 | *AC006213.1* |  |
| gen1736 | 238953_at | --- |  | ENSG00000234494 | *AC003665.1* |  |
| gen1737 | 239180_at | --- |  | ENSG00000236057 | *AC006262.1* |  |
| gen1738 | 239215_at | --- |  | ENSG00000232468 | *AL022069.1* |  |
| gen1739 | 239237_at | --- |  | ENSG00000250924 | *AC007245.1* |  |
| gen1740 | 239244_at | --- |  | ENSG00000205056 | *CLLU1* |  |
| gen1741 | 239248_at | --- |  | ENSG00000234684 | *RP11-314N13.4* |  |
| gen1742 | 239263_at | --- |  | ENSG00000234869 | *RP3-439F8.1* |  |
| gen1743 | 239284_at | --- |  | ENSG00000234405 | *LL0XNC01-250H12.3* |  |
| gen1744 | 239287_at | --- |  | ENSG00000224137 | *AC079767.4* |  |
| gen1745 | 239319_at | --- |  | ENSG00000248663 | *CTC-504A5.1* |  |
| gen1746 | 239332_at | --- |  | ENSG00000244649 | *CTD-2377D24.6* |  |
| gen1747 | 239360_at | --- |  | ENSG00000228536 | *RP11-392O17.1* |  |
| gen1748 | 239407_at | --- |  | ENSG00000238197 | *GCFC1-AS1* |  |
| gen1749 | 239420_at | --- |  | ENSG00000249601 | *CTB-27N1.1* |  |
| gen1750 | 239558_at | --- |  | ENSG00000234884 | *CTA-407F11.8* |  |
| gen1751 | 239634_at | --- |  | ENSG00000248922 | *AL137061.2* |  |
| gen1752 | 239764_at | --- |  | ENSG00000231249 | *AC024168.2* |  |
| gen1753 | 239775_at | --- |  | ENSG00000237773 | *AC003075.4* |  |
| gen1754 | 239822_at | --- |  | ENSG00000231768 | *RP5-855F14.1* |  |
| gen1755 | 239869_at | --- |  | ENSG00000251611 | *RP11-610P16.1* |  |
| gen1756 | 239873_at | --- |  | ENSG00000230289 | *RP11-334J6.6* |  |
| gen1757 | 239882_at | --- |  | ENSG00000232310 | *RP11-557H15.4* |  |
| gen1758 | 239924_at | --- |  | ENSG00000228315 | *AP000347.2* |  |
| gen1759 | 239943_x_at | --- |  | ENSG00000225436 | *AC005971.3* |  |
| gen1760 | 239999_at | --- |  | ENSG00000215386 | *C21orf34* |  |
| gen1761 | 240012_at | --- |  | ENSG00000231990 | *RP11-53B5.1* |  |
| gen1762 | 240049_at | --- |  | ENSG00000228748 | *RP13-39P12.3* |  |
| gen1763 | 240075_at | --- |  | ENSG00000233485 | *RP3-467K16.2* |  |
| gen1764 | 240212_at | --- |  | ENSG00000245213 | *RP11-10K16.1* |  |
| gen1765 | 240221_at | --- |  | ENSG00000230551 | *CTB-89H12.4* |  |
| gen1766 | 240225_at | --- |  | ENSG00000248572 | *CTD-2108O9.2* |  |
| gen1767 | 240272_at | --- |  | ENSG00000229028 | *KRT223P* |  |
| gen1768 | 240273_at | --- |  | ENSG00000235202 | *RP11-39H13.1* |  |
| gen1769 | 240305_at | --- |  | ENSG00000225140 | *RP11-809C18.3* |  |
| gen1770 | 240314_at | --- |  | ENSG00000248550 | *AL162831.1* |  |
| gen1771 | 240346_at | --- |  | ENSG00000248576 | *RP11-834C11.8* |  |
| gen1772 | 240352_at | --- |  | ENSG00000237950 | *RP11-7O11.3* |  |
| gen1773 | 240366_at | --- |  | ENSG00000226869 | *RP11-203P23.1* |  |
| gen1774 | 240380_at | --- |  | ENSG00000250436 | *RP11-622A1.2* |  |
| gen1775 | 240427_at | --- |  | ENSG00000230600 | *RP11-73B2.2* |  |
| gen1776 | 240431_at | --- |  | ENSG00000229307 | *RP11-168G22.2* |  |
| gen1777 | 240441_at | --- |  | ENSG00000205865 | *FAM99B* |  |
| gen1778 | 240447_at | --- |  | ENSG00000229348 | *RP11-506B15.6* |  |
| gen1779 | 240487_at | --- |  | ENSG00000253447 | *RP11-619L12.4* |  |
| gen1780 | 240490_at | --- |  | ENSG00000229444 | *RP11-184I16.4* |  |
| gen1781 | 240561_at | --- |  | ENSG00000232160 | *RP5-842K24.2* |  |
| gen1782 | 240574_at | --- |  | ENSG00000247400 | *DNAJC3-AS1* |  |
| gen1783 | 240584_at | --- |  | ENSG00000236262 | *SHANK2-AS2* |  |
| gen1784 | 240609_at | --- |  | ENSG00000234707 | *RP11-745C15.2* |  |
| gen1785 | 240648_at | --- |  | ENSG00000223635 | *RP4-613A2.1* |  |
| gen1786 | 240661_at | --- |  | ENSG00000224950 | *RP5-1086K13.1* |  |
| gen1787 | 240678_at | --- |  | ENSG00000237374 | *AC007680.2* |  |
| gen1788 | 240685_at | --- |  | ENSG00000248809 | *RP11-6L6.3* |  |
| gen1789 | 240707_at | --- |  | ENSG00000250250 | *CTD-2350J17.1* |  |
| gen1790 | 240745_at | --- |  | ENSG00000238276 | *RP11-245J24.1* |  |
| gen1791 | 240746_s_at | --- |  | ENSG00000238276 | *RP11-245J24.1* |  |
| gen1792 | 240755_at | --- |  | ENSG00000228707 | *RP11-787B4.2* |  |
| gen1793 | 240807_at | --- |  | ENSG00000249601 | *CTB-27N1.1* |  |
| gen1794 | 240810_at | --- |  | ENSG00000235894 | *RP11-49P4.1* |  |
| gen1795 | 240823_at | --- |  | ENSG00000226645 | *AP006216.10* |  |
| gen1796 | 240828_at | --- |  | ENSG00000235488 | *RP11-560J1.1* |  |
| gen1797 | 240842_at | --- |  | ENSG00000235122 | *RP3-417L20.4* |  |
| gen1798 | 240882_at | --- |  | ENSG00000233760 | *AC004947.2* |  |
| gen1799 | 240897_at | --- |  | ENSG00000253163 | *RP11-443C10.1* |  |
| gen1800 | 240918_at | --- |  | ENSG00000253392 | *AC006277.2* |  |
| gen1801 | 240920_at | --- |  | ENSG00000224100 | *AP001630.5* |  |
| gen1802 | 240937_at | --- |  | ENSG00000253139 | *RP11-17A4.3* |  |
| gen1803 | 240945_at | --- |  | ENSG00000230627 | *RP1-155D22.1* |  |
| gen1804 | 241067_at | --- |  | ENSG00000253959 | *CTB-43E15.1* |  |
| gen1805 | 241095_at | --- |  | ENSG00000237435 | *RP11-147C23.1* |  |
| gen1806 | 241118_at | --- |  | ENSG00000233610 | *RP11-165D7.4* |  |
| gen1807 | 241124_at | --- |  | ENSG00000248165 | *RP11-44F21.2* |  |
| gen1808 | 241126_at | --- |  | ENSG00000251175 | *RP11-45L9.1* |  |
| gen1809 | 241133_at | --- |  | ENSG00000251060 | *U66061.31* |  |
| gen1810 | 241146_at | --- |  | ENSG00000226978 | *AC006324.1* |  |
| gen1811 | 241171_at | --- |  | ENSG00000249988 | *RP11-669M16.1* |  |
| gen1812 | 241183_at | --- |  | ENSG00000242029 | *RP11-457K10.1* |  |
| gen1813 | 241187_at | --- |  | ENSG00000248624 | *RP11-5N11.2* |  |
| gen1814 | 241196_at | --- |  | ENSG00000232591 | *RP5-1031D4.2* |  |
| gen1815 | 241235_at | --- |  | ENSG00000223711 | *AC091633.3* |  |
| gen1816 | 241239_at | --- |  | ENSG00000226966 | *AC106017.4* |  |
| gen1817 | 241247_at | --- |  | ENSG00000244541 | *RP11-167H9.6* |  |
| gen1818 | 241270_at | --- |  | ENSG00000250233 | *AC015802.1* |  |
| gen1819 | 241282_at | --- |  | ENSG00000226764 | *AC010145.4* |  |
| gen1820 | 241322_at | --- |  | ENSG00000249163 | *AL121809.1* |  |
| gen1821 | 241327_at | --- |  | ENSG00000248555 | *RP11-61G23.1* |  |
| gen1822 | 241337_at | --- |  | ENSG00000229116 | *RP11-20J15.3* |  |
| gen1823 | 241432_at | --- |  | ENSG00000248228 | *RP13-511O19.1* |  |
| gen1824 | 241434_at | --- |  | ENSG00000230896 | *RP11-767N6.7* |  |
| gen1825 | 241449_at | --- |  | ENSG00000254269 | *CTD-2281E23.2* |  |
| gen1826 | 241522_at | --- |  | ENSG00000226921 | *RP11-199F6.5* |  |
| gen1827 | 241587_at | --- |  | ENSG00000230292 | *RP11-432A8.1* |  |
| gen1828 | 241743_at | --- |  | ENSG00000230084 | *RP4-613B23.1* |  |
| gen1829 | 241759_at | --- |  | ENSG00000244558 | *RP11-445H22.4* |  |
| gen1830 | 241767_at | --- |  | ENSG00000253753 | *AC145123.2* |  |
| gen1831 | 241839_at | --- |  | ENSG00000231651 | *RP11-528B10.4* |  |
| gen1832 | 241847_at | --- |  | ENSG00000229043 | *AC091729.9* |  |
| gen1833 | 241945_at | --- |  | ENSG00000248391 | *CTD-2010I22.2* |  |
| gen1834 | 242035_at | --- |  | ENSG00000225666 | *AC005534.9* |  |
| gen1835 | 242087_x_at | --- |  | ENSG00000228109 | *AC068302.3* |  |
| gen1836 | 242102_at | --- |  | ENSG00000227528 | *DIAPH3-AS1* |  |
| gen1837 | 242139_s_at | --- |  | ENSG00000142396 | *ERVK3-1* |  |
| gen1838 | 242140_at | --- |  | ENSG00000142396 | *ERVK3-1* |  |
| gen1839 | 242184_s_at | --- |  | ENSG00000253744 | *AC025442.3* |  |
| gen1840 | 242185_at | --- |  | ENSG00000253744 | *AC025442.3* |  |
| gen1841 | 242206_at | --- |  | ENSG00000244117 | *RP11-3N2.1* |  |
| gen1842 | 242222_at | --- |  | ENSG00000175772 /// ENSG00000204588 | *AC112229.7* |  |
| gen1843 | 242226_at | --- |  | ENSG00000228183 | *GS1-279B7.1* |  |
| gen1844 | 242327_x_at | --- |  | ENSG00000239644 | *RP1-163M9.6* |  |
| gen1845 | 242355_at | --- |  | ENSG00000226890 | *LA16c-395F10.1* |  |
| gen1846 | 242358_at | --- |  | ENSG00000246695 | *RP11-877E17.2* |  |
| gen1847 | 242491_at | --- |  | ENSG00000250763 | *RP11-974F13.6* |  |
| gen1848 | 242510_at | --- |  | ENSG00000225891 | *RP3-476K8.3* |  |
| gen1849 | 242518_at | --- |  | ENSG00000245849 | *CTD-2339L15.1* |  |
| gen1850 | 242521_at | --- |  | ENSG00000247217 | *AC008392.1* |  |
| gen1851 | 242666_at | --- |  | ENSG00000228839 | *RP3-400N23.6* |  |
| gen1852 | 242682_at | --- |  | ENSG00000229692 | *AC019171.5* |  |
| gen1853 | 242692_at | --- |  | ENSG00000242797 | *RP11-168J18.4* |  |
| gen1854 | 242699_at | --- |  | ENSG00000254141 | *RP11-642D21.1* |  |
| gen1855 | 242754_at | --- |  | ENSG00000244184 | *RP11-314A20.2* |  |
| gen1856 | 242766_at | --- |  | ENSG00000224905 | *AP001347.6* |  |
| gen1857 | 242798_at | --- |  | ENSG00000236519 | *AL773604.8* |  |
| gen1858 | 242808_at | --- |  | ENSG00000241170 | *RP11-147I3.1* |  |
| gen1859 | 242812_at | --- |  | ENSG00000224619 /// ENSG00000224705 /// ENSG00000228894 /// ENSG00000230660 /// ENSG00000231074 /// ENSG00000234893 /// ENSG00000235727 | *HCG18* |  |
| gen1860 | 242833_at | --- |  | ENSG00000230054 | *RP11-402G3.5* |  |
| gen1861 | 242841_at | --- |  | ENSG00000227306 | *AP006285.6* |  |
| gen1862 | 242856_at | --- |  | ENSG00000232018 | *AL132709.9* |  |
| gen1863 | 242860_at | --- |  | ENSG00000236043 | *RP11-78C6.1* |  |
| gen1864 | 242873_at | --- |  | ENSG00000245648 | *RP11-277P12.20* |  |
| gen1865 | 242888_at | --- |  | ENSG00000230082 | *AC018809.6* |  |
| gen1866 | 242914_at | --- |  | ENSG00000226249 | *RP11-307P5.2* |  |
| gen1867 | 242994_at | --- |  | ENSG00000231730 | *RP4-657D16.3* |  |
| gen1868 | 243058_at | --- |  | ENSG00000253508 | *RP1-170O19.14* |  |
| gen1869 | 243098_at | --- |  | ENSG00000231324 | *AP000696.2* |  |
| gen1870 | 243170_at | --- |  | ENSG00000241772 | *AC092620.2* |  |
| gen1871 | 243171_at | --- |  | ENSG00000229196 | *RP11-775D22.2* |  |
| gen1872 | 243190_at | --- |  | ENSG00000225751 | *AC087501.1* |  |
| gen1873 | 243248_at | --- |  | ENSG00000254143 | *RP11-470M17.2* |  |
| gen1874 | 243338_at | --- |  | ENSG00000230551 | *CTB-89H12.4* |  |
| gen1875 | 243384_at | --- |  | ENSG00000228379 /// ENSG00000229643 | *AC010891.2* |  |
| gen1876 | 243408_at | --- |  | ENSG00000248986 /// ENSG00000249145 | *RP11-774O3.1* |  |
| gen1877 | 243441_at | --- |  | ENSG00000233283 | *RP11-357H14.20* |  |
| gen1878 | 243576_at | --- |  | ENSG00000233975 | *RP11-288L9.1* |  |
| gen1879 | 243581_at | --- |  | ENSG00000229808 | *RP11-456P18.2* |  |
| gen1880 | 243634_at | --- |  | ENSG00000238206 | *RP11-472B18.2* |  |
| gen1881 | 243698_at | --- |  | ENSG00000229452 | *AC005162.4* |  |
| gen1882 | 243725_at | --- |  | ENSG00000228608 | *RP3-399C22.1* |  |
| gen1883 | 243726_at | --- |  | ENSG00000251024 | *RP11-203B7.1* |  |
| gen1884 | 243728_at | --- |  | ENSG00000249713 | *CTD-2236F14.1* |  |
| gen1885 | 243762_at | --- |  | ENSG00000225255 | *LA16c-83F12.6* |  |
| gen1886 | 243776_at | --- |  | ENSG00000250781 | *RP11-63A11.1* |  |
| gen1887 | 243813_at | --- |  | ENSG00000246430 | *RP11-16M8.2* |  |
| gen1888 | 243823_at | --- |  | ENSG00000230472 | *RP1-28O17.1* |  |
| gen1889 | 243844_at | --- |  | ENSG00000248869 | *RP11-138I17.1* |  |
| gen1890 | 243871_at | --- |  | ENSG00000235842 | *RP11-356I2.2* |  |
| gen1891 | 243873_at | --- |  | ENSG00000225063 | *RP11-383G10.5* |  |
| gen1892 | 243904_at | --- |  | ENSG00000225135 | *RP11-361F15.2* |  |
| gen1893 | 243952_at | --- |  | ENSG00000100181 | *AC005301.5* |  |
| gen1894 | 243975_at | --- |  | ENSG00000228133 | *AC099684.1* |  |
| gen1895 | 243979_at | --- |  | ENSG00000251339 | *RP11-506N2.1* |  |
| gen1896 | 243986_at | --- |  | ENSG00000227674 | *RP11-520F9.2* |  |
| gen1897 | 243996_at | --- |  | ENSG00000227388 | *RP11-112J3.16* |  |
| gen1898 | 244040_at | --- |  | ENSG00000250970 | *RP11-274N19.2* |  |
| gen1899 | 244041_at | --- |  | ENSG00000243155 | *RP11-46A10.5* |  |
| gen1900 | 244102_at | --- |  | ENSG00000230054 | *RP11-402G3.5* |  |
| gen1901 | 244127_at | --- |  | ENSG00000224441 | *AC068831.3* |  |
| gen1902 | 244135_at | --- |  | ENSG00000253695 | *RP11-177H2.2* |  |
| gen1903 | 244152_at | --- |  | ENSG00000241211 | *RP11-192K2.2* |  |
| gen1904 | 244210_at | --- |  | ENSG00000253792 | *CTC-436K13.5* |  |
| gen1905 | 244242_at | --- |  | ENSG00000230074 | *RP11-195F19.9* |  |
| gen1906 | 244252_at | --- |  | ENSG00000244953 | *RP11-613D13.8* |  |
| gen1907 | 244269_at | --- |  | ENSG00000249662 | *RP11-321E2.4* |  |
| gen1908 | 244274_at | --- |  | ENSG00000246982 | *RP1-179N16.6* |  |
| gen1909 | 244280_at | --- |  | ENSG00000228495 | *RP3-523C21.1* |  |
| gen1910 | 244283_x_at | --- |  | ENSG00000254262 | *RP11-58O3.2* |  |
| gen1911 | 244307_s_at | --- |  | ENSG00000231187 | *RP11-38L15.3* |  |
| gen1912 | 244308_at | --- |  | ENSG00000231187 | *RP11-38L15.3* |  |
| gen1913 | 244325_at | --- |  | ENSG00000233570 | *RP11-194G10.1* |  |
| gen1914 | 244372_at | --- |  | ENSG00000230525 | *AC007403.2* |  |
| gen1915 | 244381_at | --- |  | ENSG00000230325 | *RP11-385F5.4* |  |
| gen1916 | 244429_at | --- |  | ENSG00000245904 | *RP11-796E2.4* |  |
| gen1917 | 244438_at | --- |  | ENSG00000223850 | *AC010145.3* |  |
| gen1918 | 244446_at | --- |  | ENSG00000250582 | *RP11-301H24.3* |  |
| gen1919 | 244483_at | --- |  | ENSG00000224479 | *AC136289.1* |  |
| gen1920 | 244540_at | --- |  | ENSG00000233047 | *RP11-24P14.1* |  |
| gen1921 | 244553_at | --- |  | ENSG00000253769 | *AC004080.17* |  |
| gen1922 | 244570_at | --- |  | ENSG00000229931 | *RP1-151F17.1* |  |
| gen1923 | 244577_at | --- |  | ENSG00000227495 | *AC109333.10* |  |
| gen1924 | 244582_at | --- |  | ENSG00000241409 | *AC064852.4* |  |
| gen1925 | 244589_at | --- |  | ENSG00000229775 | *RP11-298H24.1* |  |
| gen1926 | 244604_at | --- |  | ENSG00000233967 | *RP11-250B2.3* |  |
| gen1927 | 244606_at | --- |  | ENSG00000229895 | *RP4-655J12.3* |  |
| gen1928 | 244701_at | --- |  | ENSG00000227136 | *C10orf101* |  |
| gen1929 | 244705_at | --- |  | ENSG00000224897 | *RP11-3B12.1* |  |
| gen1930 | 244749_at | --- |  | ENSG00000245571 | *AP001258.4* |  |
| gen1931 | 244754_at | --- |  | ENSG00000233903 | *Z83851.4* |  |
| gen1932 | 244769_at | --- |  | ENSG00000245937 | *CTC-228N24.3* |  |
| gen1933 | 244824_at | --- |  | ENSG00000236819 | *AC087393.1* |  |
| gen1934 | 244886_at | --- |  | ENSG00000246582 | *RP11-1149O23.3* |  |
| gen1935 | 51226_at | --- |  | ENSG00000253722 | *RP11-10N23.4* |  |
| gen1936 | 78047_s_at | --- |  | ENSG00000126005 | *MT1P3* |  |
| gen1937 | 221847_at | NR_036583 /// XM_001721915 /// XM_001723199 /// XM_001723624 | *LOC100129361* | --- |  | hypothetical LOC100129361 |
| gen1938 | 215658_at | NR_036581 | *LOC400573* | --- |  | hypothetical LOC400573 |
| gen1939 | 236387_at | NR_036549 | *LOC100129961* | --- |  | hypothetical LOC100129961 |
| gen1940 | 1558404_at | NR_036540 | *LOC644242* | --- |  | hypothetical LOC644242 |
| gen1941 | 229870_at | NR_036539 | *LOC644656* | --- |  | hypothetical LOC644656 |
| gen1942 | 237116_at | NR_036538 | *LOC646903* | --- |  | hypothetical LOC646903 |
| gen1943 | 1558234_at | NR_036534 | *FLJ36644* | --- |  | hypothetical LOC400617 |
| gen1944 | 228207_at | NR_036533 | *LOC100499489* | --- |  | hypothetical LOC100499489 |
| gen1945 | 235323_at | NR_036533 | *LOC100499489* | --- |  | hypothetical LOC100499489 |
| gen1946 | 238456_at | NR_036530 | *LOC100289230* | --- |  | hypothetical LOC100289230 |
| gen1947 | 1555847_a_at | NR_036515 | *LOC284454* | --- |  | hypothetical LOC284454 |
| gen1948 | 235317_at | NR_036515 | *LOC284454* | --- |  | hypothetical LOC284454 |
| gen1949 | 1559528_at | NR_036511 /// NR_036512 | *LOC100129917* | --- |  | hypothetical LOC100129917 |
| gen1950 | 240015_at | NR_036502 /// NR_036503 | *LOC439949* | --- |  | hypothetical LOC439949 |
| gen1951 | 1560371_at | NR_036501 | *LOC401321* | --- |  | hypothetical LOC401321 |
| gen1952 | 232255_at | NR_036501 | *LOC401321* | --- |  | hypothetical LOC401321 |
| gen1953 | 232256_s_at | NR_036501 | *LOC401321* | --- |  | hypothetical LOC401321 |
| gen1954 | 227885_at | NR_036500 | *LOC400236* | --- |  | Hypothetical LOC400236 |
| gen1955 | 227887_at | NR_036500 | *LOC400236* | --- |  | hypothetical LOC400236 |
| gen1956 | 1560912_at | NR_036499 | *LOC389043* | --- |  | hypothetical LOC389043 |
| gen1957 | 1556827_at | NR_036497 | *LOC339929* | --- |  | hypothetical LOC339929 |
| gen1958 | 227940_at | NR_036496 | *LOC339803* | --- |  | hypothetical LOC339803 |
| gen1959 | 231427_at | NR_036490 | *LOC284648* | --- |  | hypothetical LOC284648 |
| gen1960 | 230858_at | NR_036488 | *LOC100499467* | --- |  | hypothetical LOC100499467 |
| gen1961 | 230351_at | NR_036487 | *LOC283481* | --- |  | hypothetical LOC283481 |
| gen1962 | 243225_at | NR_036487 | *LOC283481* | --- |  | hypothetical LOC283481 |
| gen1963 | 237392_at | NR_036486 | *LOC283480* | --- |  | hypothetical LOC283480 |
| gen1964 | 1566831_at | NR_036485 | *LOC283104* | --- |  | hypothetical LOC283104 |
| gen1965 | 1558728_at | NR_036480 | *LOC100128881* | --- |  | hypothetical LOC100128881 |
| gen1966 | 229857_s_at | NR_034172 | *LOC644246* | --- |  | Hypothetical protein LOC644246 |
| gen1967 | 1554382_at | NR_034149 | *LOC200261* | --- |  | hypothetical LOC200261 |
| gen1968 | 239685_at | NR_034148 | *LOC283143* | --- |  | Hypothetical protein LOC283143 |
| gen1969 | 1559433_at | NR_034147 | *LOC149773* | --- |  | hypothetical LOC149773 |
| gen1970 | 231637_at | NR_034130 | *LOC100499194* | --- |  | hypothetical LOC100499194 |
| gen1971 | 1558310_s_at | NR_034127 | *LOC100132356* | --- |  | hypothetical LOC100132356 |
| gen1972 | 229323_at | NR_034125 | *LOC387723* | --- |  | hypothetical LOC387723 |
| gen1973 | 1558930_at | NR_034119 | *LOC728192* | --- |  | hypothetical LOC728192 |
| gen1974 | 1563062_at | NR_034119 | *LOC728192* | --- |  | Hypothetical protein LOC728192 |
| gen1975 | 1557890_at | NR_034115 | *LOC729178* | --- |  | hypothetical LOC729178 |
| gen1976 | 1562566_at | NR_034108 /// NR_034109 /// NR_034110 /// NR_034111 | *LOC643749* | --- |  | hypothetical LOC643749 |
| gen1977 | 230589_at | NR_034108 /// NR_034109 /// NR_034110 /// NR_034111 | *LOC643749* | --- |  | hypothetical LOC643749 |
| gen1978 | 240577_at | NR_034108 /// NR_034109 /// NR_034110 /// NR_034111 | *LOC643749* | --- |  | hypothetical LOC643749 |
| gen1979 | 243553_x_at | NR_034108 /// NR_034109 /// NR_034110 /// NR_034111 | *LOC643749* | --- |  | hypothetical LOC643749 |
| gen1980 | 1556117_at | NR_034104 | *LOC79015* | --- |  | hypothetical LOC79015 |
| gen1981 | 243744_at | NR_034096 | *LOC348751* | --- |  | Hypothetical protein LOC348751 |
| gen1982 | 1562803_at | NR_034087 | *C9orf29* | --- |  | Chromosome 9 open reading frame 29 |
| gen1983 | 227901_at | NR_034085 /// NR_034086 | *LOC648987* | --- |  | hypothetical LOC648987 |
| gen1984 | 233840_at | NR_034082 | *LOC100130950* | --- |  | hypothetical LOC100130950 |
| gen1985 | 244518_at | NR_034036 | *LOC100130452* | --- |  | similar to hCG1777700 |
| gen1986 | 229338_at | NR_034032 | *LOC100289361* | --- |  | hypothetical LOC100289361 |
| gen1987 | 237730_at | NR_034018 /// NR_034019 | *LOC100130700* | --- |  | hypothetical LOC100130700 |
| gen1988 | 1560147_at | NR_034012 /// NR_034013 | *LOC100131176* | --- |  | Similar to hCG1991662 |
| gen1989 | 1556402_at | NR_034004 | *LOC100132741* | --- |  | Similar to hCG1655084 |
| gen1990 | 235691_at | NR_033998 | *LOC729970* | --- |  | Similar to hCG2028352 |
| gen1991 | 233077_at | NR_033989 | *FLJ13439* | --- |  | hypothetical LOC399876 |
| gen1992 | 1562659_at | NR_033984 | *LOC400548* | --- |  | hypothetical LOC400548 |
| gen1993 | 1561090_at | NR_033983 | *LOC400654* | --- |  | hypothetical LOC400654 |
| gen1994 | 1558832_at | NR_033967 | *FLJ32224* | --- |  | hypothetical LOC440584 |
| gen1995 | 233541_at | NR_033947 | *LOC644714* | --- |  | hypothetical LOC644714 |
| gen1996 | 1568745_at | NR_033946 | *LOC646268* | --- |  | hCG1654703 |
| gen1997 | 1566760_at | NR_033929 | *FLJ34208* | --- |  | hypothetical LOC401106 |
| gen1998 | 243547_at | NR_033904 | *FLJ39639* | --- |  | hypothetical protein FLJ39639 |
| gen1999 | 1557373_at | NR_033887 | *LOC339505* | --- |  | hypothetical LOC339505 |
| gen2000 | 1561455_at | NR_033881 | *LOC284294* | --- |  | hypothetical LOC284294 |
| gen2001 | 1560573_at | NR_033878 | *LOC387895* | --- |  | hypothetical LOC387895 |
| gen2002 | 243905_at | NR_033878 | *LOC387895* | --- |  | hypothetical LOC387895 |
| gen2003 | 228832_at | NR_033874 | *FLJ20021* | --- |  | hypothetical LOC90024 |
| gen2004 | 235909_at | NR_033872 | *LOC400960* | --- |  | Hypothetical gene supported by BC040598 |
| gen2005 | 240017_at | NR_033872 | *LOC400960* | --- |  | Hypothetical gene supported by BC040598 |
| gen2006 | 216874_at | NR_033870 | *DKFZp686O1327* | --- |  | Hypothetical gene supported by BC043549; BX648102 |
| gen2007 | 216877_at | NR_033870 | *DKFZp686O1327* | --- |  | Hypothetical gene supported by BC043549; BX648102 |
| gen2008 | 1564122_at | NR_033861 | *LOC283875* | --- |  | hypothetical LOC283875 |
| gen2009 | 1561222_at | NR_033855 | *LOC283432* | --- |  | hypothetical LOC283432 |
| gen2010 | 1563867_at | NR_033853 | *LOC283194* | --- |  | hypothetical LOC283194 |
| gen2011 | 1557207_s_at | NR_033852 | *LOC283177* | --- |  | hypothetical LOC283177 |
| gen2012 | 238112_at | NR_033852 | *LOC283177* | --- |  | hypothetical LOC283177 |
| gen2013 | 1565806_at | NR_033847 | *FLJ37035* | --- |  | FLJ37035 protein |
| gen2014 | 244465_at | NR_033846 | *LOC283033* | --- |  | Hypothetical protein LOC283033 |
| gen2015 | 1566526_at | NR_033833 | *LOC283688* | --- |  | hypothetical LOC283688 |
| gen2016 | 233013_x_at | NR_033805 | *LOC220906* | --- |  | hypothetical LOC220906 |
| gen2017 | 1553069_at | NR_033800 | *NCRNA00257* | --- |  | non-protein coding RNA 257 |
| gen2018 | 231954_at | NR_033797 | *DKFZP434I0714* | --- |  | hypothetical protein DKFZP434I0714 |
| gen2019 | 1560378_at | NR_033368 | *C21orf41* | --- |  | Chromosome 21 open reading frame 41 |
| gen2020 | 222947_at | NR_033341 | *LOC100379224* | --- |  | hypothetical LOC100379224 |
| gen2021 | 1553205_at | NR_033263 | *C20orf200* | --- |  | chromosome 20 open reading frame 200 |
| gen2022 | 1566860_at | NR_033252 | *LOC145663* | --- |  | hypothetical LOC145663 |
| gen2023 | 230307_at | NR_033240 | *LOC100129794* | --- |  | similar to hCG1804255 |
| gen2024 | 229599_at | NR_029453 /// NR_029454 /// NR_029455 | *LOC440335* | --- |  | hypothetical LOC440335 |
| gen2025 | 1560619_at | NR_029448 /// NR_029449 | *LOC255411* | --- |  | hypothetical LOC255411 |
| gen2026 | 225724_at | NR_029434 /// NR_029435 | *FLJ31306* | --- |  | hypothetical LOC379025 |
| gen2027 | 239432_at | NR_029434 /// NR_029435 | *FLJ31306* | --- |  | hypothetical LOC379025 |
| gen2028 | 1555865_at | NR_029409 | *LOC255512* | --- |  | hypothetical LOC255512 |
| gen2029 | 228839_s_at | NR_029407 | *LOC642361* | --- |  | hypothetical LOC642361 |
| gen2030 | 230552_at | NR_029389 /// NR_029390 | *LOC100134317 /// LOC284412* | --- |  | hypothetical LOC100134317 /// hypothetical LOC284412 |
| gen2031 | 1561319_at | NR_029385 | *OTX2OS1* | --- |  | Otx2 opposite strand transcript 1 |
| gen2032 | 1569078_at | NR_029376 | *LOC100294362* | --- |  | hypothetical LOC100294362 |
| gen2033 | 233512_at | NR_029193 | *LOC100287216* | --- |  | hypothetical LOC100287216 |
| gen2034 | 239125_at | NR_028443 | *LOC100303728* | --- |  | hypothetical LOC100303728 |
| gen2035 | 1569378_at | NR_028415 | *LOC100292680* | --- |  | hypothetical LOC100292680 |
| gen2036 | 226413_at | NR_028408 | *LOC400027* | --- |  | hypothetical LOC400027 |
| gen2037 | 238916_at | NR_028408 | *LOC400027* | --- |  | hypothetical LOC400027 |
| gen2038 | 207799_x_at | NR_028407 | *ARMCX4* | --- |  | armadillo repeat containing, X-linked 4 |
| gen2039 | 1561373_at | NR_028390 | *LOC285796* | --- |  | hypothetical LOC285796 |
| gen2040 | 1556244_s_at | NR_028386 | *LOC375196* | --- |  | hypothetical LOC375196 |
| gen2041 | 239008_at | NR_028379 | *NCRNA00182* | --- |  | Non-protein coding RNA 182 |
| gen2042 | 237522_at | NR_028371 | *FAS-AS* | --- |  | FAS antisense RNA (non-protein coding) |
| gen2043 | 230477_at | NR_028339 /// NR_028340 | *LOC100130522* | --- |  | Hypothetical LOC100130522 |
| gen2044 | 1556588_at | NR_028330 | *C15orf37* | --- |  | chromosome 15 open reading frame 37 |
| gen2045 | 1564787_at | NR_028328 | *LOC643923* | --- |  | hypothetical LOC643923 |
| gen2046 | 1568768_s_at | NR_028308 | *LOC100302650* | --- |  | hypothetical LOC100302650 |
| gen2047 | 207894_s_at | NR_028288 | *TCL6* | --- |  | T-cell leukemia/lymphoma 6 (non-protein coding) |
| gen2048 | 219839_x_at | NR_028288 | *TCL6* | --- |  | T-cell leukemia/lymphoma 6 (non-protein coding) |
| gen2049 | 238893_at | NR_028138 | *LOC338758* | --- |  | hypothetical LOC338758 |
| gen2050 | 235587_at | NR_028090 | *LOC202781* | --- |  | hypothetical LOC202781 |
| gen2051 | 1556829_at | NR_027954 | *LOC100287227* | --- |  | hypothetical LOC100287227 |
| gen2052 | 237651_x_at | NR_027793 | *C6orf218* | --- |  | chromosome 6 open reading frame 218 |
| gen2053 | 207287_at | NR_027715 | *FLJ14107* | --- |  | hypothetical LOC80094 |
| gen2054 | 220364_at | NR_027706 | *FLJ11235* | --- |  | hypothetical FLJ11235 |
| gen2055 | 1553145_at | NR_027696 /// NR_027697 | *FLJ39653* | --- |  | hypothetical FLJ39653 |
| gen2056 | 1556033_at | NR_027468 | *FLJ39739* | --- |  | hypothetical FLJ39739 |
| gen2057 | 239005_at | NR_027468 | *FLJ39739* | --- |  | Hypothetical FLJ39739 |
| gen2058 | 1564601_at | NR_027457 | *NCRNA00221* | --- |  | non-protein coding RNA 221 |
| gen2059 | 215057_at | NR_027456 | *LOC100272228* | --- |  | hypothetical LOC100272228 |
| gen2060 | 241533_at | NR_027454 | *LOC731656* | --- |  | Hypothetical LOC731656 |
| gen2061 | 1558028_x_at | NR_027451 | *LOC647979* | --- |  | hypothetical LOC647979 |
| gen2062 | 224597_at | NR_027451 | *LOC647979* | --- |  | hypothetical LOC647979 |
| gen2063 | 232420_x_at | NR_027447 | *LOC100289341* | --- |  | similar to hCG2022304 |
| gen2064 | 243784_s_at | NR_027440 | *LOC100272217* | --- |  | hypothetical LOC100272217 |
| gen2065 | 243785_at | NR_027440 | *LOC100272217* | --- |  | hypothetical LOC100272217 |
| gen2066 | 213089_at | NR_027439 | *LOC100272216* | --- |  | hypothetical LOC100272216 |
| gen2067 | 239395_at | NR_027435 | *LOC100129716* | --- |  | hypothetical LOC100129716 |
| gen2068 | 221186_at | NR_027434 | *LOC100131532* | --- |  | hypothetical LOC100131532 |
| gen2069 | 222194_at | NR_027425 | *FAM66D* | --- |  | family with sequence similarity 66, member D |
| gen2070 | 1566251_at | NR_027418 | *LOC100499466* | --- |  | hypothetical LOC100499466 |
| gen2071 | 1566987_s_at | NR_027418 | *LOC100499466* | --- |  | hypothetical LOC100499466 |
| gen2072 | 225054_x_at | NR_027418 | *LOC100499466* | --- |  | hypothetical LOC100499466 |
| gen2073 | 225055_at | NR_027418 | *LOC100499466* | --- |  | hypothetical LOC100499466 |
| gen2074 | 227567_at | NR_027418 | *LOC100499466* | --- |  | hypothetical LOC100499466 |
| gen2075 | 1570049_at | NR_027412 /// NR_027413 | *LOC100130581* | --- |  | hypothetical LOC100130581 |
| gen2076 | 225214_at | NR_027406 | *LOC100129034* | --- |  | hypothetical LOC100129034 |
| gen2077 | 244823_at | NR_027406 | *LOC100129034* | --- |  | hypothetical LOC100129034 |
| gen2078 | 244164_at | NR_027402 | *NCRNA00204B* | --- |  | Non-protein coding RNA 204B |
| gen2079 | 1552607_at | NR_027401 | *NCRNA00204* | --- |  | non-protein coding RNA 204 |
| gen2080 | 235174_s_at | NR_027387 | *LOC100128822* | --- |  | hypothetical LOC100128822 |
| gen2081 | 1558387_at | NR_027378 | *LOC643763* | --- |  | hypothetical LOC643763 |
| gen2082 | 1558388_a_at | NR_027378 | *LOC643763* | --- |  | hypothetical LOC643763 |
| gen2083 | 239656_at | NR_027374 | *LOC723809* | --- |  | hypothetical LOC723809 |
| gen2084 | 1558195_at | NR_027358 | *LOC283404* | --- |  | hypothetical LOC283404 |
| gen2085 | 233636_at | NR_027349 /// NR_027350 | *MIR17HG* | --- |  | MIR17 host gene (non-protein coding) |
| gen2086 | 1568864_at | NR_027334 | *LOC100131691* | --- |  | hypothetical LOC100131691 |
| gen2087 | 210109_at | NR_027330 | *C7orf54* | --- |  | chromosome 7 open reading frame 54 |
| gen2088 | 1562703_at | NR_027321 | *LOC157381* | --- |  | hypothetical LOC157381 |
| gen2089 | 1557550_at | NR_027318 | *LOC148145* | --- |  | hypothetical LOC148145 |
| gen2090 | 1561085_at | NR_027311 /// NR_027312 | *LOC153910* | --- |  | hypothetical LOC153910 |
| gen2091 | 232281_at | NR_027301 | *LOC148189* | --- |  | Hypothetical LOC148189 |
| gen2092 | 235191_at | NR_027301 | *LOC148189* | --- |  | Hypothetical LOC148189 |
| gen2093 | 236653_at | NR_027301 | *LOC148189* | --- |  | Hypothetical LOC148189 |
| gen2094 | 242663_at | NR_027301 | *LOC148189* | --- |  | Hypothetical LOC148189 |
| gen2095 | 237047_at | NR_027278 | *NCRNA00281* | --- |  | non-protein coding RNA 281 |
| gen2096 | 1562930_at | NR_027274 /// NR_027275 | *LOC100128788* | --- |  | hypothetical LOC100128788 |
| gen2097 | 228889_at | NR_027263 | *C14orf128* | --- |  | chromosome 14 open reading frame 128 |
| gen2098 | 1570189_at | NR_027254 | *LOC388387* | --- |  | Hypothetical LOC388387 |
| gen2099 | 1555124_at | NR_027251 | *LOC100129726* | --- |  | hypothetical LOC100129726 |
| gen2100 | 231189_at | NR_027251 | *LOC100129726* | --- |  | hypothetical LOC100129726 |
| gen2101 | 1557146_a_at | NR_027242 | *LOC146336* | --- |  | hypothetical LOC146336 |
| gen2102 | 232463_at | NR_027231 /// NR_027232 | *NCRNA00107* | --- |  | non-protein coding RNA 107 |
| gen2103 | 1552665_at | NR_027182 | *LOC84989* | --- |  | hypothetical LOC84989 |
| gen2104 | 1553385_at | NR_027181 | *LOC84931* | --- |  | hypothetical LOC84931 |
| gen2105 | 227183_at | NR_027180 | *LOC728264* | --- |  | hypothetical LOC728264 |
| gen2106 | 231987_at | NR_027180 | *LOC728264* | --- |  | hypothetical LOC728264 |
| gen2107 | 244807_at | NR_027158 /// NR_027159 /// NR_027160 /// NR_027161 /// NR_027162 /// NR_027163 /// NR_027164 /// NR_027165 /// NR_027166 /// NR_027167 /// NR_027168 /// NR_027169 /// NR_027170 /// NR_027171 /// NR_027172 /// NR_027173 /// NR_027174 /// NR_027175 /// NR_027176 /// NR_027177 /// NR_027178 /// NR_027179 /// NR_027667 | *NCRNA00188* | --- |  | non-protein coding RNA 188 |
| gen2108 | 1557548_at | NR_027151 /// NR_027152 | *C10orf108* | --- |  | chromosome 10 open reading frame 108 |
| gen2109 | 223973_at | NR_027148 | *C19orf30* | --- |  | chromosome 19 open reading frame 30 |
| gen2110 | 238641_at | NR_027136 | *C1orf126* | --- |  | chromosome 1 open reading frame 126 |
| gen2111 | 239120_at | NR_027136 | *C1orf126* | --- |  | chromosome 1 open reading frame 126 |
| gen2112 | 1558881_at | NR_027132 /// NR_027133 | *LOC145820* | --- |  | hypothetical LOC145820 |
| gen2113 | 231381_at | NR_027122 | *ESRG* | --- |  | hypothetical LOC790952 |
| gen2114 | 1557723_at | NR_027117 | *LOC285847* | --- |  | hypothetical LOC285847 |
| gen2115 | 1557724_a_at | NR_027117 | *LOC285847* | --- |  | hypothetical LOC285847 |
| gen2116 | 1570131_at | NR_027117 | *LOC285847* | --- |  | hypothetical LOC285847 |
| gen2117 | 1557618_at | NR_027115 /// NR_027116 | *LOC285768* | --- |  | hypothetical LOC285768 |
| gen2118 | 237614_at | NR_027113 /// NR_027114 | *LOC285740* | --- |  | hypothetical LOC285740 |
| gen2119 | 243688_at | NR_027107 | *MGC45800* | --- |  | hypothetical LOC90768 |
| gen2120 | 1563462_at | NR_027105 /// NR_027106 | *LOC285419* | --- |  | Hypothetical LOC285419 |
| gen2121 | 1563082_at | NR_027098 /// NR_027099 /// NR_027100 | *LOC285045* | --- |  | hypothetical LOC285045 |
| gen2122 | 1563083_s_at | NR_027098 /// NR_027099 /// NR_027100 | *LOC285045* | --- |  | hypothetical LOC285045 |
| gen2123 | 1564309_at | NR_027098 /// NR_027099 /// NR_027100 | *LOC285045* | --- |  | hypothetical LOC285045 |
| gen2124 | 1556999_at | NR_027097 | *LOC100271832* | --- |  | hypothetical LOC100271832 |
| gen2125 | 1557474_at | NR_027086 | *LOC284578* | --- |  | hypothetical LOC284578 |
| gen2126 | 1561518_at | NR_027079 /// NR_027080 | *LOC283914* | --- |  | hypothetical LOC283914 |
| gen2127 | 1556452_a_at | NR_027074 /// NR_027075 /// NR_027076 /// NR_027077 | *LOC283761* | --- |  | hypothetical LOC283761 |
| gen2128 | 235994_s_at | NR_027064 | *PLAC2* | --- |  | Placenta-specific 2 (non-protein coding) |
| gen2129 | 1554097_a_at | NR_027054 | *LOC554202* | --- |  | hypothetical LOC554202 |
| gen2130 | 230505_at | NR_027046 | *LOC145474* | --- |  | hypothetical LOC145474 |
| gen2131 | 232298_at | NR_027037 /// NR_027038 | *LOC401093* | --- |  | hypothetical LOC401093 |
| gen2132 | 232088_x_at | NR_027036 | *LOC100271722* | --- |  | hypothetical LOC100271722 |
| gen2133 | 237021_at | NR_027035 | *LOC144486* | --- |  | hypothetical LOC144486 |
| gen2134 | 1555907_at | NR_027032 | *LOC100130776* | --- |  | hypothetical LOC100130776 |
| gen2135 | 1552899_at | NR_027029 /// NR_027030 | *MGC34034* | --- |  | hypothetical protein MGC34034 |
| gen2136 | 1552900_a_at | NR_027029 /// NR_027030 | *MGC34034* | --- |  | hypothetical protein MGC34034 |
| gen2137 | 1565554_at | NR_027022 | *LOC127841* | --- |  | hypothetical LOC127841 |
| gen2138 | 1565556_at | NR_027022 | *LOC127841* | --- |  | hypothetical LOC127841 |
| gen2139 | 240682_at | NR_027012 /// NR_027013 | *NCRNA00293* | --- |  | non-protein coding RNA 293 |
| gen2140 | 1557211_a_at | NR_027004 | *C14orf86* | --- |  | chromosome 14 open reading frame 86 |
| gen2141 | 232832_at | NR_027003 | *DKFZp434J0226* | --- |  | hypothetical LOC93429 |
| gen2142 | 232346_at | NR_027002 | *LOC388692* | --- |  | hypothetical LOC388692 |
| gen2143 | 233418_at | NR_026998 | *LOC91450* | --- |  | hypothetical LOC91450 |
| gen2144 | 233321_x_at | NR_026993 | *LOC90834* | --- |  | hypothetical protein BC001742 |
| gen2145 | 228515_at | NR_026984 | *LOC90784* | --- |  | hypothetical LOC90784 |
| gen2146 | 1558790_s_at | NR_026974 | *C8orf77* | --- |  | chromosome 8 open reading frame 77 |
| gen2147 | 216608_at | NR_026964 | *LOC26102* | --- |  | hypothetical LOC26102 |
| gen2148 | 217219_at | NR_026964 | *LOC26102* | --- |  | hypothetical LOC26102 |
| gen2149 | 1557682_a_at | NR_026957 | *LOC284688* | --- |  | hypothetical LOC284688 |
| gen2150 | 1569318_at | NR_026956 | *LOC284440* | --- |  | hypothetical LOC284440 |
| gen2151 | 233830_at | NR_026954 | *LOC90246* | --- |  | hypothetical LOC90246 |
| gen2152 | 233835_at | NR_026954 | *LOC90246* | --- |  | hypothetical LOC90246 |
| gen2153 | 1564224_x_at | NR_026947 | *LOC283314* | --- |  | hypothetical LOC283314 |
| gen2154 | 1559488_at | NR_026945 | *LOC257358* | --- |  | hypothetical LOC257358 |
| gen2155 | 1559489_a_at | NR_026945 | *LOC257358* | --- |  | hypothetical LOC257358 |
| gen2156 | 1553357_at | NR_026935 | *LOC158696* | --- |  | hypothetical LOC158696 |
| gen2157 | 222307_at | NR_026932 | *LOC282997* | --- |  | hypothetical LOC282997 |
| gen2158 | 1553898_a_at | NR_026929 | *DKFZp434L192* | --- |  | hypothetical protein DKFZp434L192 |
| gen2159 | 215105_at | NR_026928 | *CG030* | --- |  | hypothetical CG030 |
| gen2160 | 233582_at | NR_026927 | *LOC115110* | --- |  | hypothetical LOC115110 |
| gen2161 | 1553658_at | NR_026919 | *LOC150197* | --- |  | hypothetical LOC150197 |
| gen2162 | 1560751_at | NR_026908 | *C18orf16* | --- |  | chromosome 18 open reading frame 16 |
| gen2163 | 1553400_a_at | NR_026905 /// NR_026906 | *C17orf69* | --- |  | chromosome 17 open reading frame 69 |
| gen2164 | 1558611_at | NR_026905 /// NR_026906 | *C17orf69* | --- |  | Chromosome 17 open reading frame 69 |
| gen2165 | 1553881_at | NR_026902 | *MGC16142* | --- |  | hypothetical protein MGC16142 |
| gen2166 | 232634_at | NR_026894 | *DKFZp566F0947* | --- |  | hypothetical LOC94023 |
| gen2167 | 1558216_at | NR_026892 | *AFAP1-AS* | --- |  | AFAP1 antisense RNA (non-protein coding) |
| gen2168 | 223779_at | NR_026892 | *AFAP1-AS* | --- |  | AFAP1 antisense RNA (non-protein coding) |
| gen2169 | 205510_s_at | NR_026891 | *FLJ10038* | --- |  | hypothetical protein FLJ10038 |
| gen2170 | 205511_at | NR_026891 | *FLJ10038* | --- |  | hypothetical protein FLJ10038 |
| gen2171 | 236164_at | NR_026891 | *FLJ10038* | --- |  | hypothetical protein FLJ10038 |
| gen2172 | 234905_at | NR_026889 | *DKFZP434H168* | --- |  | hypothetical LOC26077 |
| gen2173 | 220465_at | NR_026887 | *LOC80054* | --- |  | hypothetical LOC80054 |
| gen2174 | 229722_at | NR_026885 | *LOC100270804* | --- |  | hypothetical LOC100270804 |
| gen2175 | 234356_at | NR_026882 | *DKFZP434K028* | --- |  | hypothetical LOC26070 |
| gen2176 | 224507_s_at | NR_026880 | *MGC12916* | --- |  | hypothetical protein MGC12916 |
| gen2177 | 224508_at | NR_026880 | *MGC12916* | --- |  | hypothetical protein MGC12916 |
| gen2178 | 238664_s_at | NR_026880 | *MGC12916* | --- |  | hypothetical protein MGC12916 |
| gen2179 | 211718_at | NR_026877 | *MGC2889* | --- |  | hypothetical protein MGC2889 |
| gen2180 | 1563496_at | NR_026876 | *STL* | --- |  | six-twelve leukemia |
| gen2181 | 1553226_at | NR_026869 | *NCRNA00052* | --- |  | non-protein coding RNA 52 |
| gen2182 | 227412_at | NR_026862 | *PPP1R3E* | --- |  | protein phosphatase 1, regulatory (inhibitor) subunit 3E |
| gen2183 | 1566555_at | NR_026857 | *FLJ90757* | --- |  | hypothetical LOC440465 |
| gen2184 | 229829_at | NR_026849 | *C18orf18* | --- |  | chromosome 18 open reading frame 18 |
| gen2185 | 1563606_a_at | NR_026847 | *LOC286359* | --- |  | hypothetical LOC286359 |
| gen2186 | 1563607_x_at | NR_026847 | *LOC286359* | --- |  | hypothetical LOC286359 |
| gen2187 | 227424_x_at | NR_026845 | *C21orf119* | --- |  | chromosome 21 open reading frame 119 |
| gen2188 | 1565684_at | NR_026833 | *LOC400940* | --- |  | hypothetical LOC400940 |
| gen2189 | 1565685_at | NR_026833 | *LOC400940* | --- |  | hypothetical LOC400940 |
| gen2190 | 1553796_at | NR_026832 | *LOC150622* | --- |  | hypothetical LOC150622 |
| gen2191 | 1553797_a_at | NR_026832 | *LOC150622* | --- |  | hypothetical LOC150622 |
| gen2192 | 236739_at | NR_026832 | *LOC150622* | --- |  | hypothetical LOC150622 |
| gen2193 | 233566_at | NR_026827 | *LOC84856* | --- |  | hypothetical LOC84856 |
| gen2194 | 232430_at | NR_026817 | *LOC148696* | --- |  | hypothetical LOC148696 |
| gen2195 | 208109_s_at | NR_026813 | *C15orf5* | --- |  | chromosome 15 open reading frame 5 |
| gen2196 | 220918_at | NR_026812 | *C21orf96* | --- |  | chromosome 21 open reading frame 96 |
| gen2197 | 220575_at | NR_026809 | *FAM106A* | --- |  | family with sequence similarity 106, member A |
| gen2198 | 220710_at | NR_026808 | *C15orf28* | --- |  | chromosome 15 open reading frame 28 |
| gen2199 | 220211_at | NR_026806 | *FLJ13224* | --- |  | hypothetical LOC79857 |
| gen2200 | 1561106_at | NR_026805 | *NCRNA00271* | --- |  | non-protein coding RNA 271 |
| gen2201 | 206478_at | NR_026800 | *KIAA0125* | --- |  | KIAA0125 |
| gen2202 | 1555786_s_at | NR_026796 /// NR_026797 | *C14orf34* | --- |  | chromosome 14 open reading frame 34 |
| gen2203 | 1559950_at | NR_026788 /// NR_027425 | *FAM66C /// FAM66D* | --- |  | family with sequence similarity 66, member C /// family with sequence similarity 66, member D |
| gen2204 | 1559952_x_at | NR_026788 /// NR_027425 | *FAM66C /// FAM66D* | --- |  | family with sequence similarity 66, member C /// family with sequence similarity 66, member D |
| gen2205 | 1570395_a_at | NR_026788 | *FAM66C* | --- |  | family with sequence similarity 66, member C |
| gen2206 | 1570396_at | NR_026788 | *FAM66C* | --- |  | family with sequence similarity 66, member C |
| gen2207 | 1570397_x_at | NR_026788 | *FAM66C* | --- |  | family with sequence similarity 66, member C |
| gen2208 | 1568854_at | NR_026775 | *NCRNA00240* | --- |  | non-protein coding RNA 240 |
| gen2209 | 1569476_at | NR_026771 | *DKFZP434L187* | --- |  | hypothetical LOC26082 |
| gen2210 | 216596_at | NR_026771 | *DKFZP434L187* | --- |  | hypothetical LOC26082 |
| gen2211 | 230861_at | NR_026771 | *DKFZP434L187* | --- |  | Hypothetical LOC26082 |
| gen2212 | 221129_at | NR_026770 | *C17orf88* | --- |  | chromosome 17 open reading frame 88 |
| gen2213 | 1554715_at | NR_026764 | *C15orf50* | --- |  | chromosome 15 open reading frame 50 |
| gen2214 | 1554716_s_at | NR_026764 | *C15orf50* | --- |  | chromosome 15 open reading frame 50 |
| gen2215 | 225225_at | NR_026757 | *LOC729082* | --- |  | Hypothetical protein LOC729082 |
| gen2216 | 1559028_at | NR_026755 | *C21orf15* | --- |  | chromosome 21 open reading frame 15 |
| gen2217 | 234445_at | NR_026751 | *ZNRD1-AS* | --- |  | ZNRD1 antisense RNA (non-protein coding) |
| gen2218 | 234866_s_at | NR_026751 | *ZNRD1-AS* | --- |  | ZNRD1 antisense RNA (non-protein coding) |
| gen2219 | 242557_at | NR_026751 | *NCRNA00171* | --- |  | Non-protein coding RNA 171 |
| gen2220 | 243017_at | NR_026742 | *LOC158572* | --- |  | hypothetical LOC158572 |
| gen2221 | 243061_at | NR_026731 /// NR_026732 | *C14orf23* | --- |  | chromosome 14 open reading frame 23 |
| gen2222 | 216053_x_at | NR_026713 | *FAM182A* | --- |  | Family with sequence similarity 182, member A |
| gen2223 | 1552872_at | NR_026710 /// NR_026711 | *ASMTL-AS* | --- |  | ASMTL antisense RNA (non-protein coding) |
| gen2224 | 1552873_s_at | NR_026710 /// NR_026711 | *ASMTL-AS* | --- |  | ASMTL antisense RNA (non-protein coding) |
| gen2225 | 241354_at | NR_026710 /// NR_026711 | *ASMTL-AS* | --- |  | ASMTL antisense RNA (non-protein coding) |
| gen2226 | 244875_at | NR_026710 /// NR_026711 | *ASMTL-AS* | --- |  | ASMTL antisense RNA (non-protein coding) |
| gen2227 | 1566916_at | NR_026684 | *HPYR1* | --- |  | Helicobacter pylori responsive 1 (non-protein coding) |
| gen2228 | 1560278_at | NR_026681 | *LOC221122* | --- |  | hypothetical LOC221122 |
| gen2229 | 1560279_a_at | NR_026681 | *LOC221122* | --- |  | hypothetical LOC221122 |
| gen2230 | 1559171_at | NR_026680 /// NR_027295 | *MGC57346* | --- |  | hypothetical LOC401884 |
| gen2231 | 1559291_at | NR_026679 /// NR_026687 | *NCRNA00032* | --- |  | Non-protein coding RNA 32 |
| gen2232 | 1559292_s_at | NR_026679 /// NR_026687 | *NCRNA00032* | --- |  | Non-protein coding RNA 32 |
| gen2233 | 1559293_x_at | NR_026679 /// NR_026687 | *NCRNA00032* | --- |  | Non-protein coding RNA 32 |
| gen2234 | 1553258_at | NR_026674 | *FLJ30679* | --- |  | hypothetical protein FLJ30679 |
| gen2235 | 1553747_at | NR_026664 | *MGC16025* | --- |  | hypothetical LOC85009 |
| gen2236 | 1553811_at | NR_026661 /// NR_026662 | *MGC14436* | --- |  | hypothetical LOC84983 |
| gen2237 | 1557451_at | NR_026651 | *DGCR10* | --- |  | DiGeorge syndrome critical region gene 10 |
| gen2238 | 1568662_at | NR_026647 | *PWRN2* | --- |  | Prader-Willi region non-protein coding RNA 2 |
| gen2239 | 1568663_a_at | NR_026647 | *PWRN2* | --- |  | Prader-Willi region non-protein coding RNA 2 |
| gen2240 | 1556262_at | NR_026646 | *PWRN1* | --- |  | Prader-Willi region non-protein coding RNA 1 |
| gen2241 | 1556263_s_at | NR_026646 | *PWRN1* | --- |  | Prader-Willi region non-protein coding RNA 1 |
| gen2242 | 1562873_at | NR_026646 | *PWRN1* | --- |  | Prader-Willi region non-protein coding RNA 1 |
| gen2243 | 1569819_at | NR_026646 | *PWRN1* | --- |  | Prader-Willi region non-protein coding RNA 1 |
| gen2244 | 242292_at | NR_026594 | *NCRNA00246B* | --- |  | non-protein coding RNA 246B |
| gen2245 | 1554591_at | NR_026555 | *GDEP* | --- |  | Gene differentially expressed in prostate |
| gen2246 | 220887_at | NR_024630 | *C14orf162* | --- |  | chromosome 14 open reading frame 162 |
| gen2247 | 220852_at | NR_024620 | *PRO1768* | --- |  | PRO1768 |
| gen2248 | 229699_at | NR_024618 | *LOC100129550* | --- |  | hypothetical LOC100129550 |
| gen2249 | 215972_at | NR_024617 /// NR_028508 /// NR_028509 | *PART1* | --- |  | prostate androgen-regulated transcript 1 (non-protein coding) |
| gen2250 | 208247_at | NR_024615 | *C3orf51* | --- |  | chromosome 3 open reading frame 51 |
| gen2251 | 238422_at | NR_024606 | *LOC151534* | --- |  | hypothetical LOC151534 |
| gen2252 | 1558477_at | NR_024594 | *LOC100131496* | --- |  | hypothetical LOC100131496 |
| gen2253 | 238473_at | NR_024586 | *LOC100216545* | --- |  | hypothetical LOC100216545 |
| gen2254 | 1562598_at | NR_024585 | *LOC100128292* | --- |  | hypothetical LOC100128292 |
| gen2255 | 1554448_at | NR_024582 | *NCRNA00183* | --- |  | non-protein coding RNA 183 |
| gen2256 | 1558782_a_at | NR_024567 | *LOC100130557* | --- |  | hypothetical LOC100130557 |
| gen2257 | 1563367_at | NR_024559 | *LOC100128977* | --- |  | hypothetical LOC100128977 |
| gen2258 | 237331_s_at | NR_024559 | *LOC100128977* | --- |  | hypothetical LOC100128977 |
| gen2259 | 237536_at | NR_024559 | *LOC100128977* | --- |  | hypothetical LOC100128977 |
| gen2260 | 1554927_at | NR_024505 /// NR_024506 /// NR_024507 | *LOC646982* | --- |  | twelve-thirteen translocation leukemia gene |
| gen2261 | 1555264_a_at | NR_024505 /// NR_024506 /// NR_024507 | *LOC646982* | --- |  | twelve-thirteen translocation leukemia gene |
| gen2262 | 233432_at | NR_024505 /// NR_024506 /// NR_024507 | *LOC646982* | --- |  | twelve-thirteen translocation leukemia gene |
| gen2263 | 240230_s_at | NR_024495 | *LOC642826* | --- |  | hypothetical LOC642826 |
| gen2264 | 220352_x_at | NR_024492 | *FLJ42627* | --- |  | hypothetical LOC645644 |
| gen2265 | 226900_at | NR_024490 | *LOC100129387* | --- |  | hypothetical LOC100129387 |
| gen2266 | 235990_at | NR_024469 | *LOC100130987* | --- |  | similar to hCG1815675 |
| gen2267 | 1564670_at | NR_024461 /// NR_024462 | *LOC100190938* | --- |  | hypothetical LOC100190938 |
| gen2268 | 232731_x_at | NR_024461 /// NR_024462 | *LOC100190938* | --- |  | hypothetical LOC100190938 |
| gen2269 | 235060_at | NR_024456 | *LOC100190986* | --- |  | hypothetical LOC100190986 |
| gen2270 | 235167_at | NR_024456 | *LOC100190986* | --- |  | hypothetical LOC100190986 |
| gen2271 | 232579_at | NR_024451 | *LOC100134229* | --- |  | hypothetical LOC100134229 |
| gen2272 | 1559045_at | NR_024447 | *LOC100128288* | --- |  | hypothetical LOC100128288 |
| gen2273 | 1564280_x_at | NR_024443 | *LOC100133920* | --- |  | hypothetical LOC100133920 |
| gen2274 | 225381_at | NR_024430 | *LOC399959* | --- |  | hypothetical LOC399959 |
| gen2275 | 1560119_at | NR_024420 | *LOC389634* | --- |  | hypothetical LOC389634 |
| gen2276 | 1564426_x_at | NR_024420 | *LOC389634* | --- |  | hypothetical LOC389634 |
| gen2277 | 240002_at | NR_024420 | *FAM93B /// LOC389634* | --- |  | family with sequence similarity 93, member B /// hypothetical LOC389634 |
| gen2278 | 237450_at | NR_024418 | *LOC389332* | --- |  | hypothetical LOC389332 |
| gen2279 | 241249_at | NR_024408 | *LOC253039* | --- |  | hypothetical LOC253039 |
| gen2280 | 234925_at | NR_024406 | *LOC732275* | --- |  | similar to hCG1645603 |
| gen2281 | 213248_at | NR_024403 /// NR_024405 | *LOC730101* | --- |  | hypothetical LOC730101 |
| gen2282 | 1556154_a_at | NR_024399 /// NR_024402 | *MGC23284* | --- |  | hypothetical LOC197187 |
| gen2283 | 1568683_at | NR_024399 /// NR_024402 | *MGC23284* | --- |  | hypothetical LOC197187 |
| gen2284 | 1553780_at | NR_024396 | *MGC23270* | --- |  | hypothetical LOC196872 |
| gen2285 | 236753_at | NR_024394 | *LOC154822* | --- |  | hypothetical LOC154822 |
| gen2286 | 237471_at | NR_024394 | *LOC154822* | --- |  | hypothetical LOC154822 |
| gen2287 | 243261_at | NR_024394 | *LOC154822* | --- |  | hypothetical LOC154822 |
| gen2288 | 239509_at | NR_024389 | *FLJ16779* | --- |  | hypothetical LOC100192386 |
| gen2289 | 1561707_at | NR_024381 | *LOC150185* | --- |  | hypothetical LOC150185 |
| gen2290 | 1561590_a_at | NR_024369 | *LOC415056* | --- |  | hypothetical LOC415056 |
| gen2291 | 238678_at | NR_024368 | *FLJ45340* | --- |  | hypothetical LOC402483 |
| gen2292 | 1553586_at | NR_024348 | *NCRNA00095* | --- |  | non-protein coding RNA 95 |
| gen2293 | 229734_at | NR_024344 | *LOC283174* | --- |  | hypothetical LOC283174 |
| gen2294 | 232123_at | NR_024344 | *LOC283174* | --- |  | hypothetical LOC283174 |
| gen2295 | 1559311_at | NR_024341 | *FLJ40292* | --- |  | hypothetical LOC643210 |
| gen2296 | 236835_at | NR_024334 | *LOC645431* | --- |  | hypothetical LOC645431 |
| gen2297 | 242889_x_at | NR_024334 | *LOC645431* | --- |  | hypothetical LOC645431 |
| gen2298 | 235014_at | NR_024333 | *LOC147727* | --- |  | hypothetical LOC147727 |
| gen2299 | 227992_s_at | NR_024330 | *NCRNA00085* | --- |  | non-protein coding RNA 85 |
| gen2300 | 220399_at | NR_024321 | *NCRNA00115* | --- |  | non-protein coding RNA 115 |
| gen2301 | 229090_at | NR_024284 | *LOC220930* | --- |  | hypothetical LOC220930 |
| gen2302 | 227593_at | NR_024279 | *FLJ37453* | --- |  | hypothetical LOC729614 |
| gen2303 | 227596_at | NR_024279 | *FLJ37453* | --- |  | Hypothetical LOC729614 |
| gen2304 | 1560006_a_at | NR_024278 | *LOC646762* | --- |  | hypothetical LOC646762 |
| gen2305 | 1568597_at | NR_024278 | *LOC646762* | --- |  | hypothetical LOC646762 |
| gen2306 | 223740_at | NR_024277 | *NCRNA00241* | --- |  | non-protein coding RNA 241 |
| gen2307 | 1564697_a_at | NR_024270 | *LOC400752* | --- |  | hypothetical LOC400752 |
| gen2308 | 225493_at | NR_024266 | *LOC144438* | --- |  | hypothetical LOC144438 |
| gen2309 | 233311_at | NR_024264 | *LOC145845* | --- |  | hypothetical LOC145845 |
| gen2310 | 1559276_at | NR_024259 | *LOC728606* | --- |  | hypothetical LOC728606 |
| gen2311 | 1564060_at | NR_024246 | *LOC144742* | --- |  | hypothetical LOC144742 |
| gen2312 | 1552258_at | NR_024204 /// NR_024205 /// NR_024206 | *NCRNA00152* | --- |  | non-protein coding RNA 152 |
| gen2313 | 234816_at | NR_024182 /// NR_024183 /// NR_024184 | *C14orf48* | --- |  | chromosome 14 open reading frame 48 |
| gen2314 | 215003_at | NR_024159 | *DGCR9* | --- |  | DiGeorge syndrome critical region gene 9 |
| gen2315 | 215725_at | NR_024157 | *DGCR11* | --- |  | DiGeorge syndrome critical region gene 11 |
| gen2316 | 1562733_at | NR_024129 | *NCRNA00092* | --- |  | non-protein coding RNA 92 |
| gen2317 | 236623_at | NR_024124 /// NR_024125 /// NR_024126 /// NR_027645 /// NR_027646 | *ATP1A1OS* | --- |  | ATP1A1 opposite strand |
| gen2318 | 237088_at | NR_024119 | *NCRNA00244* | --- |  | non-protein coding RNA 244 |
| gen2319 | 239421_at | NR_024101 | *FLJ35776* | --- |  | Hypothetical LOC649446 |
| gen2320 | 1556414_at | NR_024092 | *C21orf71* | --- |  | chromosome 21 open reading frame 71 |
| gen2321 | 213486_at | NR_024086 | *COPG2IT1* | --- |  | COPG2 imprinted transcript 1 (non-protein coding) |
| gen2322 | 232975_at | NR_024052 /// NR_024053 | *HCG18* | --- |  | HLA complex group 18 |
| gen2323 | 1560834_a_at | NR_024037 | *RMST* | --- |  | rhabdomyosarcoma 2 associated transcript (non-protein coding) |
| gen2324 | 1562633_at | NR_024037 | *RMST* | --- |  | rhabdomyosarcoma 2 associated transcript (non-protein coding) |
| gen2325 | 229782_at | NR_024037 | *RMST* | --- |  | rhabdomyosarcoma 2 associated transcript (non-protein coding) |
| gen2326 | 1570345_at | NR_024032 | *C9orf27* | --- |  | Chromosome 9 open reading frame 27 |
| gen2327 | 223896_at | NR_024013 | *FKSG29* | --- |  | FKSG29 |
| gen2328 | 229015_at | NR_024011 | *LOC286367* | --- |  | FP944 |
| gen2329 | 1556661_at | NR_024009 | *CN5H6.4* | --- |  | hypothetical LOC150384 |
| gen2330 | 1553020_at | NR_024007 | *SMCR5* | --- |  | Smith-Magenis syndrome chromosome region, candidate 5 (non-protein coding) |
| gen2331 | 214344_at | NR_024006 | *LOC92973* | --- |  | hypothetical LOC92973 |
| gen2332 | 218820_at | NR_023938 | *C14orf132* | --- |  | chromosome 14 open reading frame 132 |
| gen2333 | 224181_at | NR_023925 /// NR_023926 /// NR_023927 /// NR_023928 | *C18orf2* | --- |  | chromosome 18 open reading frame 2 |
| gen2334 | 234712_at | NR_023925 /// NR_023926 /// NR_023927 /// NR_023928 | *C18orf2* | --- |  | chromosome 18 open reading frame 2 |
| gen2335 | 227446_s_at | NR_023921 /// NR_023922 /// NR_023923 /// NR_023924 | *C14orf167* | --- |  | chromosome 14 open reading frame 167 |
| gen2336 | 206954_at | NR_023920 | *WT1-AS* | --- |  | WT1 antisense RNA (non-protein coding) |
| gen2337 | 213447_at | NR_023915 | *IPW* | --- |  | imprinted in Prader-Willi syndrome (non-protein coding) |
| gen2338 | 221974_at | NR_023915 | *IPW* | --- |  | imprinted in Prader-Willi syndrome (non-protein coding) |
| gen2339 | 241834_at | NR_023915 | *IPW* | --- |  | imprinted in Prader-Willi syndrome (non-protein coding) |
| gen2340 | 228200_at | NR_023392 | *ZNF252* | --- |  | zinc finger protein 252 |
| gen2341 | 233399_x_at | NR_023392 | *ZNF252* | --- |  | Zinc finger protein 252 |
| gen2342 | 239753_at | NR_023392 | *ZNF252* | --- |  | zinc finger protein 252 |
| gen2343 | 239799_at | NR_023389 /// NR_023390 | *C9orf130* | --- |  | chromosome 9 open reading frame 130 |
| gen2344 | 1559324_at | NR_023380 | *CCDC144C* | --- |  | Coiled-coil domain containing 144C |
| gen2345 | 214834_at | NR_022008 | *PAR5* | --- |  | Prader-Willi/Angelman syndrome-5 |
| gen2346 | 1556768_at | NR_021493 | *LOC100144604* | --- |  | hypothetical LOC100144604 |
| gen2347 | 238557_at | NR_021492 | *LOC100144603* | --- |  | hypothetical transcript |
| gen2348 | 1566219_at | NR_021489 | *LOC338651* | --- |  | hypothetical LOC338651 |
| gen2349 | 226793_at | NR_015451 | *NCRNA00294* | --- |  | non-protein coding RNA 294 |
| gen2350 | 232645_at | NR_015447 | *LOC153684* | --- |  | hypothetical LOC153684 |
| gen2351 | 225934_at | NR_015434 | *LOC148413* | --- |  | hypothetical LOC148413 |
| gen2352 | 243716_at | NR_015431 /// NR_024153 | *FLJ43663* | --- |  | hypothetical LOC378805 |
| gen2353 | 1559097_at | NR_015430 | *C14orf64* | --- |  | chromosome 14 open reading frame 64 |
| gen2354 | 1560499_at | NR_015430 | *C14orf64* | --- |  | chromosome 14 open reading frame 64 |
| gen2355 | 213788_s_at | NR_015427 | *NCRNA00094* | --- |  | non-protein coding RNA 94 |
| gen2356 | 231024_at | NR_015423 | *LOC572558* | --- |  | hypothetical locus LOC572558 |
| gen2357 | 229013_at | NR_015419 | *LOC145783* | --- |  | hypothetical LOC145783 |
| gen2358 | 1560734_at | NR_015416 | *LOC727924* | --- |  | hypothetical LOC727924 |
| gen2359 | 1564855_at | NR_015416 | *LOC727924* | --- |  | hypothetical LOC727924 |
| gen2360 | 1564856_s_at | NR_015416 | *LOC727924* | --- |  | hypothetical LOC727924 |
| gen2361 | 1557063_at | NR_015415 | *FLJ45244* | --- |  | hypothetical locus FLJ45244 |
| gen2362 | 235936_at | NR_015411 | *LOC254559* | --- |  | hypothetical LOC254559 |
| gen2363 | 238603_at | NR_015411 | *LOC254559* | --- |  | hypothetical LOC254559 |
| gen2364 | 1562469_at | NR_015410 | *FLJ22536* | --- |  | hypothetical locus LOC401237 |
| gen2365 | 229280_s_at | NR_015410 | *FLJ22536* | --- |  | hypothetical locus LOC401237 |
| gen2366 | 241047_at | NR_015410 | *FLJ22536* | --- |  | hypothetical locus LOC401237 |
| gen2367 | 241671_x_at | NR_015410 | *FLJ22536* | --- |  | hypothetical locus LOC401237 |
| gen2368 | 234054_at | NR_015409 | *LOC143188* | --- |  | hypothetical LOC143188 |
| gen2369 | 219817_at | NR_015404 | *C12orf47* | --- |  | chromosome 12 open reading frame 47 |
| gen2370 | 1564207_at | NR_015401 /// NR_024416 | *FLJ35390* | --- |  | hypothetical LOC255031 |
| gen2371 | 1564208_x_at | NR_015401 /// NR_024416 | *FLJ35390* | --- |  | hypothetical LOC255031 |
| gen2372 | 1562876_s_at | NR_015395 /// NR_024373 | *LOC541471* | --- |  | Hypothetical LOC541471 |
| gen2373 | 232918_at | NR_015395 /// NR_024373 | *LOC541471* | --- |  | Hypothetical LOC541471 |
| gen2374 | 215283_at | NR_015389 | *LOC339290* | --- |  | hypothetical LOC339290 |
| gen2375 | 217506_at | NR_015389 | *LOC339290* | --- |  | Hypothetical LOC339290 |
| gen2376 | 226235_at | NR_015389 | *LOC339290* | --- |  | hypothetical LOC339290 |
| gen2377 | 228160_at | NR_015389 | *LOC339290* | --- |  | hypothetical LOC339290 |
| gen2378 | 243426_at | NR_015389 | *LOC339290* | --- |  | hypothetical LOC339290 |
| gen2379 | 236102_at | NR_015384 | *LOC100126784* | --- |  | Hypothetical LOC100126784 |
| gen2380 | 239141_at | NR_015384 | *LOC100126784* | --- |  | hypothetical LOC100126784 |
| gen2381 | 240407_at | NR_015384 | *LOC100126784* | --- |  | hypothetical LOC100126784 |
| gen2382 | 1562924_at | NR_015383 | *LOC340357* | --- |  | hypothetical LOC340357 |
| gen2383 | 232462_s_at | NR_015380 | *A1BG-AS* | --- |  | A1BG antisense RNA (non-protein coding) |
| gen2384 | 229014_at | NR_015369 /// NR_021490 /// NR_021491 | *FLJ42709* | --- |  | hypothetical LOC441094 |
| gen2385 | 227201_at | NR_015368 | *LOC643837* | --- |  | hypothetical LOC643837 |
| gen2386 | 235497_at | NR_015368 | *LOC643837* | --- |  | hypothetical LOC643837 |
| gen2387 | 215297_at | NR_015364 | *LOC441204* | --- |  | hypothetical locus LOC441204 |
| gen2388 | 240194_at | NR_015364 | *LOC441204* | --- |  | hypothetical locus LOC441204 |
| gen2389 | 1555872_a_at | NR_015363 /// XM_001128866 /// XM_001718591 | *LOC728903 /// MGC21881* | --- |  | hypothetical LOC728903 /// hypothetical locus MGC21881 |
| gen2390 | 1555874_x_at | NR_015363 | *MGC21881* | --- |  | hypothetical locus MGC21881 |
| gen2391 | 236902_at | NR_015358 | *FLJ43390* | --- |  | hypothetical LOC646113 |
| gen2392 | 212957_s_at | NR_015353 | *LOC92249* | --- |  | hypothetical LOC92249 |
| gen2393 | 216838_at | NR_015353 | *LOC92249* | --- |  | hypothetical LOC92249 |
| gen2394 | 232572_at | NR_015342 | *PCA3* | --- |  | Prostate cancer antigen 3 (non-protein coding) |
| gen2395 | 239617_at | NR_004431 /// NR_024144 /// NR_024145 /// NR_024146 | *GHRLOS* | --- |  | ghrelin opposite strand (non-protein coding) |
| gen2396 | 244695_at | NR_004431 /// NR_024144 /// NR_024145 /// NR_024146 | *GHRLOS* | --- |  | ghrelin opposite strand (non-protein coding) |
| gen2397 | 225699_at | NR_003697 | *C7orf40* | --- |  | chromosome 7 open reading frame 40 |
| gen2398 | 229899_s_at | NR_003604 /// NR_003605 /// NR_003606 /// NR_036658 /// NR_036659 | *NCRNA00275* | --- |  | non-protein coding RNA 275 |
| gen2399 | 240607_at | NR_003491 /// NR_033319 /// NR_033320 /// NR_033321 | *MIAT* | --- |  | Myocardial infarction associated transcript (non-protein coding) |
| gen2400 | 216249_at | NR_003367 | *PVT1* | --- |  | Pvt1 oncogene (non-protein coding) |
| gen2401 | 231592_at | NR_003255 | *TSIX* | --- |  | XIST antisense RNA (non-protein coding) |
| gen2402 | 1567359_at | NR_002832 /// NR_033312 /// NR_033313 /// NR_033314 /// NR_033315 | *BDNFOS* | --- |  | BDNF opposite strand (non-protein coding) |
| gen2403 | 223577_x_at | NR_002819 | *MALAT1* | --- |  | metastasis associated lung adenocarcinoma transcript 1 (non-protein coding) |
| gen2404 | 223578_x_at | NR_002819 | *MALAT1* | --- |  | metastasis associated lung adenocarcinoma transcript 1 (non-protein coding) |
| gen2405 | 223940_x_at | NR_002819 | *MALAT1* | --- |  | metastasis associated lung adenocarcinoma transcript 1 (non-protein coding) |
| gen2406 | 224559_at | NR_002819 | *MALAT1* | --- |  | metastasis associated lung adenocarcinoma transcript 1 (non-protein coding) |
| gen2407 | 224567_x_at | NR_002819 | *MALAT1* | --- |  | metastasis associated lung adenocarcinoma transcript 1 (non-protein coding) |
| gen2408 | 224568_x_at | NR_002819 | *MALAT1* | --- |  | metastasis associated lung adenocarcinoma transcript 1 (non-protein coding) |
| gen2409 | 226675_s_at | NR_002819 | *MALAT1* | --- |  | metastasis associated lung adenocarcinoma transcript 1 (non-protein coding) |
| gen2410 | 227510_x_at | NR_002819 | *MALAT1* | --- |  | metastasis associated lung adenocarcinoma transcript 1 (non-protein coding) |
| gen2411 | 228582_x_at | NR_002819 | *MALAT1* | --- |  | Metastasis associated lung adenocarcinoma transcript 1 (non-protein coding) |
| gen2412 | 231735_s_at | NR_002819 | *MALAT1* | --- |  | metastasis associated lung adenocarcinoma transcript 1 (non-protein coding) |
| gen2413 | 214186_s_at | NR_002812 | *HCG26* | --- |  | HLA complex group 26 (non-protein coding) |
| gen2414 | 226369_at | NR_002809 | *LOC338799* | --- |  | hypothetical LOC338799 |
| gen2415 | 241886_x_at | NR_002809 | *LOC338799* | --- |  | Hypothetical LOC338799 |
| gen2416 | 1563524_a_at | NR_002808 | *ITPK1-AS* | --- |  | ITPK1 antisense RNA (non-protein coding) |
| gen2417 | 234594_at | NR_002808 | *ITPK1-AS* | --- |  | ITPK1 antisense RNA (non-protein coding) |
| gen2418 | 232531_at | NR_002791 | *EMX2OS* | --- |  | EMX2 opposite strand (non-protein coding) |
| gen2419 | 1560542_at | NR_002776 | *MCM3AP-AS* | --- |  | MCM3AP antisense RNA (non-protein coding) |
| gen2420 | 1559326_at | NR_002770 | *DIO3-OS* | --- |  | DIO3 opposite strand (non-protein coding) |
| gen2421 | 223757_at | NR_002770 | *DIO3-OS* | --- |  | DIO3 opposite strand (non-protein coding) |
| gen2422 | 234515_at | NR_002769 | *PCGEM1* | --- |  | prostate-specific transcript 1 (non-protein coding) |
| gen2423 | 215513_at | NR_002768 | *HYMAI* | --- |  | hydatidiform mole associated and imprinted (non-protein coding) |
| gen2424 | 220530_at | NR_002767 | *NCRNA00120* | --- |  | non-protein coding RNA 120 |
| gen2425 | 1558144_at | NR_002766 /// NR_003530 /// NR_003531 /// NR_033358 /// NR_033359 /// NR_033360 | *MEG3* | --- |  | maternally expressed 3 (non-protein coding) |
| gen2426 | 222328_x_at | NR_002766 /// NR_003530 /// NR_003531 /// NR_033358 /// NR_033359 /// NR_033360 | *MEG3* | --- |  | Maternally expressed 3 (non-protein coding) |
| gen2427 | 229557_at | NR_002766 /// NR_003530 /// NR_003531 /// NR_033358 /// NR_033359 /// NR_033360 | *MEG3* | --- |  | maternally expressed 3 (non-protein coding) |
| gen2428 | 231529_at | NR_002766 /// NR_003530 /// NR_003531 /// NR_033358 /// NR_033359 /// NR_033360 | *MEG3* | --- |  | Maternally expressed 3 (non-protein coding) |
| gen2429 | 235077_at | NR_002766 /// NR_003530 /// NR_003531 /// NR_033358 /// NR_033359 /// NR_033360 | *MEG3* | --- |  | maternally expressed 3 (non-protein coding) |
| gen2430 | 220694_at | NR_002765 | *ASAP1-IT* | --- |  | ASAP1 intronic transcript (non-protein coding) |
| gen2431 | 220479_at | NR_002763 | *CPS1-IT* | --- |  | CPS1 intronic transcript (non-protein coding) |
| gen2432 | 237249_at | NR_002728 | *KCNQ1OT1* | --- |  | KCNQ1 overlapping transcript 1 (non-protein coding) |
| gen2433 | 243428_at | NR_002728 | *KCNQ1OT1* | --- |  | KCNQ1 overlapping transcript 1 (non-protein coding) |
| gen2434 | 243435_at | NR_002728 | *KCNQ1OT1* | --- |  | KCNQ1 overlapping transcript 1 (non-protein coding) |
| gen2435 | 244727_at | NR_002728 | *KCNQ1OT1* | --- |  | KCNQ1 overlapping transcript 1 (non-protein coding) |
| gen2436 | 216404_at | NR_002717 | *ATXN8OS* | --- |  | ATXN8 opposite strand (non-protein coding) |
| gen2437 | 1556820_a_at | NR_002612 | *DLEU2* | --- |  | deleted in lymphocytic leukemia 2 (non-protein coding) |
| gen2438 | 1556821_x_at | NR_002612 | *DLEU2* | --- |  | deleted in lymphocytic leukemia 2 (non-protein coding) |
| gen2439 | 1564443_at | NR_002612 | *DLEU2* | --- |  | deleted in lymphocytic leukemia 2 (non-protein coding) |
| gen2440 | 242854_x_at | NR_002612 | *DLEU2* | --- |  | deleted in lymphocytic leukemia 2 (non-protein coding) |
| gen2441 | 1560402_at | NR_002578 | *GAS5* | --- |  | growth arrest-specific 5 (non-protein coding) |
| gen2442 | 228397_at | NR_002323 | *TUG1* | --- |  | taurine upregulated 1 (non-protein coding) |
| gen2443 | 229301_at | NR_002323 | *TUG1* | --- |  | taurine upregulated 1 (non-protein coding) |
| gen2444 | 230635_at | NR_002323 | *TUG1* | --- |  | taurine upregulated 1 (non-protein coding) |
| gen2445 | 235942_at | NR_002160 /// NR_002161 | *NCRNA00230A /// NCRNA00230B* | --- |  | non-protein coding RNA 230A /// non-protein coding RNA 230B |
| gen2446 | 206685_at | NR_002139 | *HCG4* | --- |  | HLA complex group 4 |
| gen2447 | 235446_at | NR_001564 | *XIST* | --- |  | X (inactive)-specific transcript (non-protein coding) |
| gen2448 | 1561185_at | NR_001534 | *TTTY7* | --- |  | testis-specific transcript, Y-linked 7 (non-protein coding) |
