# Supplementary material for: Circulating long non-coding RNA GAS5 (growth arrest-specific transcript 5) as a complement marker for the detection of malignant mesothelioma using liquid biopsies
Source: Biomark Res. 2020 May 13;8:15. doi: 10.1186/s40364-020-00194-4 (PMC7222324; doi:10.1186/s40364-020-00194-4)
Supplement: Supplementary file 5 — Additional file 5. Raw Ct values of long non-coding RNAs (lncRNAs) in plasma samples. [file 40364_2020_194_MOESM5_ESM.docx]

**Additional file 5.** Raw Ct values of long non-coding RNAs (lncRNAs) in plasma samples.

| **Sample** | **Group** | **Gene** | | | | | | | | | | |
| --- | --- | --- | --- | --- | --- | --- | --- | --- | --- | --- | --- | --- |
|  |  | ***AFAP1-AS*** | ***GAS5*** | ***LOC84856*** | ***PVT1*** | ***NCRNA00201*** | ***AGAP2-AS1*** | ***CRNDE*** | ***HCG18*** | ***LOC100506710*** | ***LOC642852*** | ***SNHG17*** |
| 54 | Asbestos-exposed controls | 40,00 | 30,00 | 40,00 | 40,00 | 40,00 | 40,00 | 40,00 | 40,00 | 40,00 | 40,00 | 40,00 |
| 55 | Asbestos-exposed controls | 40,00 | 29,09 | 40,00 | 40,00 | 40,00 | 40,00 | 40,00 | 40,00 | 40,00 | 40,00 | 40,00 |
| 56 | Asbestos-exposed controls | 40,00 | 28,53 | 40,00 | 40,00 | 40,00 | 40,00 | 40,00 | 40,00 | 40,00 | 40,00 | 40,00 |
| 57 | Asbestos-exposed controls | 40,00 | 28,93 | 40,00 | 40,00 | 40,00 | 40,00 | 40,00 | 40,00 | 40,00 | 40,00 | 40,00 |
| 58 | Asbestos-exposed controls | 40,00 | 28,07 | 40,00 | 31,45 | 40,00 | 40,00 | 40,00 | 40,00 | 40,00 | 40,00 | 40,00 |
| 59 | Mesothelioma patients | 40,00 | 40,00 | 40,00 | 40,00 | 40,00 | 40,00 | 40,00 | 40,00 | 40,00 | 40,00 | 40,00 |
| 60 | Mesothelioma patients | 40,00 | 27,82 | 40,00 | 40,00 | 40,00 | 40,00 | 40,00 | 40,00 | 40,00 | 40,00 | 40,00 |
| 61 | Mesothelioma patients | 40,00 | 28,94 | 40,00 | 40,00 | 40,00 | 40,00 | 40,00 | 40,00 | 30,82 | 40,00 | 40,00 |
| 62 | Mesothelioma patients | 40,00 | 28,81 | 40,00 | 40,00 | 32,93 | 40,00 | 40,00 | 40,00 | 40,00 | 40,00 | 31,55 |
